# Improving the Estimation of Prediction Increment Measures in Logistic and Survival Analysis

**DOI:** 10.3390/cancers17081259

**Published:** 2025-04-08

**Authors:** Danielle M. Enserro, Austin Miller

**Affiliations:** Department of Biostatistics and Bioinformatics, Roswell Park Comprehensive Cancer Center, Buffalo, NY 14263, USA; austin.miller@roswellpark.org

**Keywords:** risk prediction, validation, discrimination, confidence interval estimation, bootstrap

## Abstract

**Background/Objectives:** Proper confidence interval estimation of the area under the receiver operating characteristic curve (AUC), the net reclassification index (NRI), and the integrated discrimination improvement (IDI) is an area of ongoing research. The most common confidence interval estimation methods employ asymptotic theory. However, developments demonstrate that degeneration of the normal distribution assumption under the null hypothesis exists for measures such as the change in AUC (ΔAUC) and IDI, and confidence intervals estimated under the normal distribution assumption may be invalid. We aim to study the performance of confidence intervals derived assuming asymptotic theory and those derived with non-parametric bootstrapping methods. **Methods:** We examine the performance of ΔAUC, NRI, and IDI in both the logistic and survival regression context. We explore empirical distributions and compare coverage probabilities of asymptotic confidence intervals with those produced from bootstrapping methods through simulation. **Results:** The primary finding in both the logistic framework and the survival analysis framework is that the percentile CIs performed well regarding coverage, without compromise to their width; this finding was robust in most scenarios. **Conclusions:** Our results suggest that the asymptotic intervals are only appropriate when a strong effect size of the added parameter exists, and that the percentile bootstrap interval exhibits at least a reasonable coverage while maintaining the shortest width in nearly all simulated scenarios, making this interval the most reliable choice. The intent is that these recommendations improve the accuracy in the estimation and the overall assessment of discrimination improvement.

## Simple Summary

Confidence interval estimation of discrimination improvement measures, including the area under the receiver operating characteristic curve, the net reclassification index, and the integrated discrimination improvement, is an area of ongoing research. The most common confidence interval estimation methods employ normal theory. Literature suggests that degeneration of the normal assumption under the null hypothesis exists, and normal theory confidence intervals estimated may be invalid. Bootstrapped confidence intervals do not rely on normal theory assumptions. We examine the performance of discrimination improvement measures in both the logistic and survival regression context through simulation. Normal theory intervals are only appropriate with a strong effect size of the added parameter, and the percentile bootstrap interval exhibits reasonable coverage while maintaining the shortest width in nearly all simulated scenarios, making this interval the most reliable choice. The intent is that these recommendations improve the accuracy in the estimation and the overall assessment of discrimination improvement.

## 1. Introduction

In the pursuit of eradicating cancer, a key area of research is cancer prevention and control. Both observational and treatment trials may assess collected patient risk factors for their associations with cancer survival outcomes, such as objective tumor response, progression-free survival, and overall survival. Cancer risk prediction models can be developed and utilized by health care providers to assess a patient’s risk of developing cancer given specific patient characteristics. An example of a model used for breast cancer risk assessment is the National Cancer Institute’s Breast Cancer Risk Assessment Tool (BCRAT), which was originally developed by Mitchell Gail and known as the Gail Model [1].

It is important that established risk prediction models are accurate, reliable, and robust for their application in the population at-risk for cancer development or recurrence. The common practice is to develop the risk prediction model on a discovery data set, followed by assessing the model performance through internal or external validation, or some combination [2,3]. The BCRAT model and its performance were later assessed with external validation in a range of diverse populations [4,5,6,7]. Common statistical modeling techniques for model development and validation include logistic regression for binary outcomes and Cox proportional hazards (PH) regression for time-to-event outcomes [8].

Risk prediction model validation first requires establishing clinical and statistical significance of the marker of interest [9]. Additionally, model calibration and discrimination should be assessed: calibration quantifies how close the predictions are to the observed outcomes while discrimination quantifies the model’s ability to correctly distinguish between events and nonevents [8]. A common area of ongoing research is taking previously developed and validated models and updating them with the addition of new markers to improve both model calibration and discrimination. This paper focuses on the improvement in discrimination by adding a new marker to a standard risk model.

### 1.1. ΔAUC for Evaluating Discrimination Improvement

There are multiple measures for assessing discrimination improvement. The most common metric used in logistic regression is the difference in area under the receiver operating characteristic curve (AUC), where ΔAUC = AUC(expanded model) − AUC(standard model) [10]. The overall C is an extension of the AUC for survival analysis, and ΔC can be calculated as C(expanded model) − C(standard model) [11]. For comparing nested models, a strong added marker should theoretically increase the AUC or the overall C. Pepe et al. demonstrated that if the model is correct under normality, then the significance of the new marker is equivalent to significant improvement in AUC [9]. However, the magnitude of ΔAUC often fails to recognize some promising new markers, the paradox being that a statistically significant marker will fail to produce a large increase in AUC, especially if the standard model’s baseline AUC is large [12]. This phenomenon motivated a search for new methods for assessing a predictor’s incremental value [13,14].

### 1.2. NRI, NRI > 0, and IDI: Definitions and Controversaries

Pencina et al. proposed new measures for assessing discrimination in logistic regression including a risk-category-based measure known as the net reclassification index (NRI), the continuous NRI (NRI > 0), and the integrated discrimination improvement (IDI) [12,15]. Extensions of these measures were later derived for survival analysis [16]. The NRI attempts to quantify whether a new variable provides clinically relevant improvement in risk prediction, assuming that a valuable marker will tend to increase predicted risks for events and decrease predicted risks for nonevents [12]. Calculation is based on the categorization of model-based predicted probabilities from models being compared into ordinal categories of absolute risk by event status, which are cross-tabulated in a reclassification table. NRI typically assumes either two or three risk categories, but it can also assume many. In the case that risk categories are not clinically assignable, the definition was extended so that model-based predicted probabilities from the standard prediction model and the updated prediction model can be compared directly with NRI > 0 [15]. Finally, IDI accounts for the size of movements up and down in risk categories, jointly quantifying the overall improvement in sensitivity and specificity over all possible cutoffs on the (0, 1) interval [12].

NRI and NRI > 0 have been subject to criticism. Pepe argued that NRI as a single summary measure is less clinically relevant than the event NRI (eNRI) and nonevent NRI (neNRI) as separate components, and Kerr et al. demonstrate scenarios where the sum of eNRI and neNRI mask detriment by the new model within either the events or nonevents [17,18]. NRI’s interpretation is commonly mistaken as a proportion, and Pepe argues it is better to interpret eNRI and neNRI separately, since eNRI is the net proportion of events assigned a higher risk and neNRI is the net proportion of nonevents assigned a lower risk [17]. Kerr et al. show that NRI > 0 can be dramatically large in magnitude, even for models with an uninformative added marker, thus alluding that null new markers are predictive and falsely leading investigators to incorrectly assign them roles in risk prediction [18]. Hilden and Gerds argue that the estimation of NRI > 0 and IDI may not be valid with poorly calibrated risk models and that model re-calibration may not correct all issues [19,20]. Leening et al. counter-argue that mean calibration of risk prediction models is automatically achieved by any maximum likelihood estimation (MLE) based algorithm and therefore Hilden and Gerds’ example is not applicable [21]. They also reiterate the importance of ensuring properly calibrated models before evaluating the inherent value of an added marker [21]. Additionally, McKeague and Qian show that the empirical distribution of NRI > 0 can be bimodal in nature [22]. With the amount of conflicting literature, investigators should take great care in the estimation and interpretation of NRI, NRI > 0, and IDI, as these measures can be estimated in conjunction with other measures of discrimination improvement such as ΔAUC [17,21].

### 1.3. Challenges in Estimation of Discrimination Improvement Measures

Proper confidence interval (CI) estimation of these metrics is important to consider. The most common confidence interval estimation methods employ asymptotic theory. However, developments demonstrate that degeneration of the normal distribution assumption under the null hypothesis exists for discrimination improvement metrics, and confidence intervals estimated under the normal distribution assumption may be invalid. Demler et al. demonstrated misuse of the DeLong test comparing AUCs for two nested models, observing that the empirical distribution of ΔAUC was highly right-skewed with the median near zero, suggesting that the distribution of the ΔAUC estimator under the null is dramatically different from the normal distribution assumed by the DeLong test [23]. Additionally, Kerr et al. demonstrated via simulation that the standard error formula of IDI underestimates the standard error, and that the normal theory test testing the null hypothesis of IDI = 0 is not valid due to violation of the normal assumption [24]. We hypothesize that this phenomenon also exists for NRI and NRI > 0. The asymptotic CI formulas rely heavily on the assumption that the distribution of these measures is always normal in nature, which may not be the case, especially for weak added factors. They also rely on proper estimation of standard errors; the developed formulas for the standard errors of NRI and IDI may not be correctly estimating the true variance.

Alternative methods for CI estimation employ bootstrapping techniques and do not assume normality under the null. However, with the potential for large data, deriving CIs from bootstrapping can be computationally intensive, so we must ensure that what we gain from bootstrap CIs is worth the extra time and computer power. In this paper, we examine the performance of ΔAUC, NRI, NRI > 0, and IDI in the binary outcome framework and their extensions in survival analysis. We explore empirical distributions and compare the coverage probabilities for CIs derived from various methods. We consider two event rates, several effect sizes, two sample size options, and two types of censoring for survival analysis. Finally, we make recommendations for CI methods to utilize based on their performance.

## 2. Methods

### 2.1. General Framework: Logistic Regression

We let Y be a binary outcome, where Y = 1 for an event of interest and Y = 0 for a nonevent. We define a column vector X of *p + q* predictor variables conditional on Y that follows a multivariate normal distribution for nonevents X|Y = 0~*N*(µ_0_, Σ) and for events X|Y = 1~*N*(µ_1_, Σ). While we assume equal variance for events and non-events, we clarify that this is not a necessary assumption. We assume that Y and X are available for all N patients. We also assume that the *p + q* predictor variables of X are uncorrelated. We use X to predict Y; a projection based on the full set of *p + q* predictors compared with a projection based on a reduced set of predictors, *p*, where the reduced model is nested within the full model. In logistic regression, the respective models produce linear coefficient estimates a’ = (a_1_,…, a_p+q_), based on the full model, and b’ = (b_1_,…, b_p_), based on the reduced model, with risk scores functions of a’X_p+q_ and b’X_p_. We assume here that higher values of risk scores correspond to a higher probability of the event of interest. We wish to test whether the last *q* coefficients are equal to 0 and to then determine whether the full model discriminates as well as or better between the two subgroups of Y as compared to the reduced model.

### 2.2. General Framework: Time-to-Event Regression

Logistic regression modeling can be employed in scenarios with full follow-up. While collecting complete follow-up on all participants is ideal, situations without complete follow-up time are more realistic. Survival analysis methods, such as Kaplan–Meier and Cox proportional hazards regressions, have become some of the most used tools in risk prediction modeling [3,8,25]. We assume there is a time-to-event variable T for all individuals, such that T_i_ represents either when an event has occurred or the time of censoring, where censoring can occur as either loss to follow-up or at the end of the study. All individuals also have an event indicator Y, where Y = 1 for those with the event observed or Y = 0 for those censored. We also have collected data on *p + q* fixed covariates at baseline in the vector X, made up of x_1_, …, x_p+q_. We assume that the censoring is non-informative, meaning that the censoring mechanism is unrelated to the outcome of interest or the covariates, and we also assume right-censoring for the event times. Thus, if the proportionality of hazards assumption is true, we can use the Cox proportional hazards (PH) regression model [26]. The fitted risk model can then be used to estimate the predicted probability of survival within the specified time frame (0, *t*). Measuring discrimination improvement in survival analysis is complicated by the fact that the event of interest involves a time-to-event component, and the model thus makes predictions about these survival times.

### 2.3. Measures of Discrimination Improvement

#### 2.3.1. AUC and Overall C

The area under the receiver operating characteristic (ROC) curve (AUC) is defined as the probability that the risk of event for a randomly selected nonevent is less than the risk of event for a randomly selected event. Given a risk score threshold *t*, sensitivity (or true positive fraction, TPF) is the probability that an event is classified correctly as an event:

Sensitivity = TPF = P(risk score > *t* | Y = 1)

Specificity is defined as the probability of correctly classifying a nonevent:

Specificity = P(risk score < *t* | Y = 0)

Each risk score is compared to *t* for classification of the subject with that risk score to a predicted group, i.e., predicted event vs. predicted nonevent. Subtracting specificity from 1 yields the false positive fraction (FPF):

1 − Specificity = FPF = P(risk score > *t*) | Y = 0)

Depending on the choice of threshold *t*, some events and nonevents will be correctly classified while the remainder will be misclassified. Varying *t* yields different pairs of values of TPF and FPF. Plotting these pairs (FPF, TPF) for all possible choices of *t* results in the ROC curve. The AUC is the area under the curve, which ranges from 0.5 (no discriminatory ability) to 1.0 (perfect discrimination).

There is no explicit formula for the AUC, so typically it is estimated by the Mann–Whitney statistic [10]. This is a non-parametric, unbiased estimator, referred to as npAUC and known as the C-statistic [25]., e.g., for the full model discussed with risk scores a’X_p+q_, the formula is

$$C\;\equiv \;\mathrm{npAUC}=\frac{1}{n_{0}n_{1}}\sum_{x_{i}\in Y_{0}}\sum_{x_{j}\in Y_{1}}I\left[a^{\prime }x_{i},\;a\prime x_{j}\right],$$

where Y_1_ and Y_0_ are the sets of subjects with and without events, respectively, n_1_ and n_0_ are the sizes of the sets, and *I* is given as follows:

$$I\left[a^{\prime }x_{i},\;a\prime x_{j}\right]=\left\{\begin{array}{c} 1,\;if\;a^{\prime }x_{i}<a^{\prime }x_{j}\;\\ 0.5,\;if\;a^{\prime }x_{i}=a^{\prime }x_{j}\\ 0,\;otherwise\; \end{array}\right.$$

DeLong et al. developed an approximate test for testing if two AUCs from different models on the same group of subjects are equal and produced a CI for ∆AUC. The approximate standard error used in both calculations is

$$SE\left(∆AUC\right)={\left[\left(1,-1\right)S(1,-1)\right]}^{1/2}$$

where ***S*** is the estimated variance-covariance matrix of (npAUC_p+q_, npAUC_p_) [10]. We use the C-statistic as the AUC estimator in this study. To assess improvement in discrimination, we estimate the difference in area under of the receiver operating characteristic curve (AUC) for two models: ∆AUC = AUC(new or updated model) − AUC(standard model) [10].

We now extend the AUC to the survival analysis context. Using the survival regression method described, we denote the predicted survival time for everyone at baseline, T_1_, T_2_, …, T_n_. We have two categories of subjects at a given time point T ≤ T_final_: those who developed the event during the study (Y = 1) and those who were censored (Y = 0). We denote these actual survival times by U_1_, U_2_, …, U_n_. Harrell states that predicted probabilities of survival until any fixed time point (which we denote V_1_, V_2_, …, V_n_) can be used in place of the corresponding T_1_, T_2_, …, T_n_ if those estimates remain in one-to-one correspondence [25]. M. Pencina et al. show that this point holds true in the most common models in survival analysis, specifically accelerated failure time models and proportional hazards models [11].

Now we consider all pairs of subjects, (*i*, *j*), such that *i* < *j*, to ensure no repetitions. Thus, for a given pair, we have a concordant pair if U_i_ < U_j_ and V_i_ < V_j_ or U_i_ > U_j_ and V_i_ > V_j_, and we have a discordant pair if U_i_ < U_j_ and V_i_ > V_j_ or U_i_ > U_j_ and V_i_ < V_j_. Since not all pairs will be concordant or discordant due to ties, we can only use the usable pairs of subjects in which at least one had an event in the construction of the C index, resulting in either event vs. event or event vs. nonevent comparisons. The overall C is defined as the proportion of all usable concordant pairs:

$$C=\frac{\pi_{c}}{\pi_{c}+\pi_{d}}$$

where

$$\pi_{c}=P\left(U_{i}<U_{j}\;and\;V_{i}<V_{j}\;or\;U_{i}>U_{j}\;and\;V_{i}>V_{j}\right)\;=P\left(U_{i}<U_{j}\;and\;V_{i}<V_{j}\right)+P(U_{i}>U_{j}\;and\;V_{i}>V_{j})$$

$$\pi_{d}=P\left(U_{i}<U_{j}\;and\;V_{i}>V_{j}\;or\;U_{i}>U_{j}\;and\;V_{i}<V_{j}\right)\;=P\left(U_{i}<U_{j}\;and\;V_{i}>V_{j}\right)+P(U_{i}>U_{j}\;and\;V_{i}<V_{j})$$

M. Pencina et al. [11] demonstrate that since *i* and *j* are interchangeable in the definitions of π_c_ and π_d_, and since the V’s have a continuous distribution (assuming at least one predictor is continuous), the overall C index reduces to

$$C=\frac{P(U_{i}<U_{j}\;and\;V_{i}<V_{j})}{P(U_{i}<U_{j})}=P(V_{i}<V_{j}/U_{i}<U_{j}).$$

A natural estimate for the overall C is

$$\hat{C}=\frac{1}{Q}\sum_{(i,j)\in O}c_{ij}$$

where O is the set of all usable pairs of subjects (*i*, *j*), Q is the total number of usable pairs, and c_ij_ takes on the value of 1 for concordant pairs and 0 otherwise [27]. To assess improvement in discrimination, we estimate the difference in overall Cs for two models: ∆C = C(expanded model) − C(standard model).

#### 2.3.2. NRI, NRI > 0, and NRI(t)

Pencina et al. introduced the net reclassification improvement (NRI) to quantify whether a new variable provides clinically relevant improvement in risk prediction, assuming a valuable new marker will tend to increase predicted risks for events and decrease predicted risks for nonevents [12]. Calculation is based on the categorization of model-based predicted probabilities from the standard risk prediction model and the updated prediction model into clinically meaningful ordinal categories of absolute risk, which are cross-tabulated in a reclassification table. The table tabulates events and nonevents separately. Observations are categorized into the table cells based on assigned risk categories by the standard (old) risk model and the updated (new) risk model. Upward movement is defined as moving into a higher category based on the updated model, while downward movement is defined as moving into a lower category. For those where Y = 1, upward movement is preferable while downward movement is problematic. For those where Y = 0, the opposite preferences hold. Thus, the NRI is defined as:

NRI = [P(up|Y = 1) − P(down|Y = 1)] + [P(down|Y = 0) − P(up|Y = 0)].

The quantity in the first bracket is referred to as the event NRI (eNRI), or the net proportion of events assigned a higher risk category. The quantity in the second bracket is referred to as the nonevent NRI (neNRI), or the net proportion of non-events assigned a lower risk category. Each component contains a penalty for any wrong movement in categories. The probabilities can be estimated using sample data obtained from the reclassification table:

$$\hat{P}(\mathrm{up}|Y=1)={\hat{p}}_{up,Y=1}=\frac{\#\;events\;moving\;up}{\#\;events}$$

$$\hat{P}(down|Y=1)={\hat{p}}_{down,Y=1}=\frac{\#\;events\;moving\;down}{\#\;events}$$

$$\hat{P}(\mathrm{up}|Y=0)={\hat{p}}_{up,Y=0}=\frac{\#\;nonevents\;moving\;up}{\#\;nonevents}$$

$$\hat{P}(down|Y=0)={\hat{p}}_{down,Y=0}=\frac{\#\;nonevents\;moving\;down}{\#\;nonevents}.$$

Thus, NRI can be estimated as

$$\hat{NRI}=\hat{eNRI}+\hat{neNRI}$$

where

$$\hat{eNRI}=({\hat{p}}_{up,Y=1}-{\hat{p}}_{down,Y=1})$$

$$\hat{neNRI}=({\hat{p}}_{down,Y=0}-{\hat{p}}_{up,Y=0}$$

The asymptotic standard errors for eNRI and neNRI can be estimated as

$$SE\left(\hat{eNRI}\right)=\sqrt{\frac{{\hat{p}}_{up,Y=1}+{\hat{p}}_{down,Y=1}}{n_{1}}-\frac{{\left({\hat{p}}_{up,Y=1}-{\hat{p}}_{down,Y=1}\right)}^{2}}{n_{1}}}$$

$$SE\left(\hat{neNRI}\right)=\sqrt{\frac{{\hat{p}}_{down,Y=0}+{\hat{p}}_{up,Y=0}}{n_{0}}-\frac{{\left({\hat{p}}_{down,Y=0}-{\hat{p}}_{up,Y=0}\right)}^{2}}{n_{0}}}$$

and

$$SE\left(\hat{NRI}\right)=\sqrt{{SE(\hat{eNRI})}^{2}+{SE(\hat{neNRI})}^{2}}$$

where *n_1_* is the number of events and *n_0_* is the number of nonevents [12].

NRI with categories typically assumes either two or three risk categories but can assume up to *k* risk categories. The choice for risk category cutoffs is of much debate. Pencina et al. recommended that clinically useful category cutoffs should be used, but the choice of category cutoff may not be easy to make [12]. Pencina et al. later recommended that under circumstances that the choice in category cutoff is not obvious, investigators should consider using the event rate as the category cutoff, which yields an NRI measure with relationships with metrics in decision analytics and possesses meaningful interpretations [28].

In the scenario where clinically meaningful categories are not assigned, Pencina et al. extended the definition of NRI so that model-based predicted probabilities from the standard prediction model and the updated prediction model can be compared directly [15]. Instead of tabulating upward and downward movement in categories, increases and decreases in predicted probabilities are tabulated instead. This method of calculating NRI is often referred to as continuous NRI (NRI > 0), which can be defined as follows:

NRI > 0 = [P(p_new_ > p_old_|Y = 1) − P(p_new_ < p_old_|Y = 1)]

– [P(p_new_ > p_old_|Y = 0) − P(p_new_ < p_old_|Y = 0)],

where p_new_ are the predicted probabilities from the new updated risk prediction model and p_old_ are the predicted probabilities from the standard risk prediction model. Similar calculations as those shown above in the definition for NRI with categories can be used for estimating the components of NRI > 0; instead of counting the number of upward and downward movements, the number of observations that satisfy the above risk comparisons are assessed, for both events and nonevents.

The asymptotic standard errors can be estimated as

$$SE\left(\hat{eNRI>0}\right)=\sqrt{\frac{{\hat{p}}_{risk1>risk0,Y=1}+{\hat{p}}_{risk1<risk0,Y=1}}{n_{1}}-\frac{{\left({\hat{p}}_{risk1>risk0,Y=1}-{\hat{p}}_{risk1<risk0,Y=1}\right)}^{2}}{n_{1}}}$$

$$SE\left(\hat{neNRI>0}\right)=\sqrt{\frac{{\hat{p}}_{risk1<risk0,Y=0}+{\hat{p}}_{risk1>risk0,Y=0}}{n_{0}}-\frac{{\left({\hat{p}}_{risk1<risk0,Y=0}-{\hat{p}}_{risk1>risk0,Y=0}\right)}^{2}}{n_{0}}}$$

$$SE\left(\hat{NRI>0}\right)=\sqrt{{SE(\hat{eNRI>0})}^{2}+{SE(\hat{neNRI>0})}^{2}}$$

where each $\hat{p}$ represents the empirical proportions of individuals satisfying their respective criteria and the subscripts *risk1* and *risk0* represent the predicted probability of event in the updated model and the standard model, respectively [15].

NRI was later extended to survival analysis. Chambless et al. express event NRI (NRI_Y=1_) and nonevent NRI (NRI_Y=0_) as:

$${NRI}_{Y=1}=P\left(Z_{up}|Y=1\right)-P(Z_{down}|Y=1)$$

$${NRI}_{Y=0}=P\left(Z_{down}|Y=0\right)-P(Z_{up}|Y=0)$$

where overall NRI is estimated as the weighted average of the two quantities:

$${NRI}_{w}=w\cdot {NRI}_{Y=1}+\left(1-w\right)\cdot {NRI}_{Y=0},$$

with *w* between 0 and 1 [16]. A reminder that Z = X’β, and Z_up_ represents upward movement in risk category or increased risk by the new model while Z_down_ represents downward movement in risk category or decreased risk by the new model. The authors point out that using this notation, the NRI considered by Pencina et al. was 2∙NRI_0.5_, and that the choice of *w* can be used to reflect relative costs and benefits of improvement in risk prediction [12,16].

Chambless et al. made some modifications to NRI for use in survival analysis, conditioning on time being less than some *t* [16]:

$${NRI}_{Y=1}(t)=P\left(Z_{up}|Y\left(t\right)=1\right)-P(Z_{down}|Y(t)=1),$$

$${NRI}_{Y=0}(t)=P\left(Z_{down}|Y\left(t\right)=0\right)-P(Z_{up}|Y(t)=0),$$

$${NRI(t)}_{w}=w\cdot {NRI}_{Y=1}(t)+\left(1-w\right)\cdot {NRI}_{Y=0}(t).$$

A count estimator for NRI(t) uses the following proportions for event NRI(t) and nonevent NRI(t):

$${\hat{NRI}}_{Y=1}{\left(t\right)}_{count}=\frac{\sum I(Z_{up},Y\left(t\right)=1)-\sum I(Z_{down},Y\left(t\right)=1)}{\sum I(Y\left(t\right)=1)},$$

$${\hat{NRI}}_{Y=0}{\left(t\right)}_{count}=\frac{\sum I(Z_{down},Y\left(t\right)=0)-\sum I(Z_{up},Y\left(t\right)=0)}{\sum I(Y\left(t\right)=0)}.$$

We obtain the components of NRI(t) by applying Bayes’ theorem:

$${NRI}_{Y=1}\left(t\right)=\left(\frac{P(Y\left(t\right)=1|Z_{up})\cdot P(Z_{up})}{P(Y\left(t\right)=1)}-\frac{P(Y\left(t\right)=1|Z_{down})\cdot P(Z_{down})}{P(Y\left(t\right)=1)}\right),$$

$${NRI}_{Y=0}\left(t\right)=\left(\frac{P(Y\left(t\right)=0|Z_{down})\cdot P(Z_{down})}{P(Y\left(t\right)=0)}-\frac{P(Y\left(t\right)=0|Z_{up})\cdot P(Z_{up})}{P(Y\left(t\right)=0)}\right).$$

Assuming *w* = 0.5 and *p = P(Y = 1)*, we obtain

$$NRI=2\cdot {NRI}_{0.5}\left(t\right)=\frac{\left(P\left(Y\left(t\right)=1|Z_{up}\right)-p\right)\cdot P\left(Z_{up}\right)-\left(P\left(Y\left(t\right)=1|Z_{down}\right)-p\right)\cdot P\left(Z_{down}\right)}{p\cdot (1-p)}.$$

Kaplan–Meier estimates are used to estimate $P\left(Y\left(t\right)=1|Z_{up}\right)$, $P\left(Y\left(t\right)=1|Z_{down}\right)$, and p = P(Y(t) = 1) to account for censoring, thus giving us our estimate for NRI_w_(t) [16]. This expression can be used for both NRI with categories (with upward and downward movement in categories of predicted risks) or for continuous NRI (where predicted risks between new and old models are compared directly), which corresponds nicely to the original derivation of NRI [12,15].

#### 2.3.3. IDI and IDI(t)

Pencina et al. also introduced the integrated discrimination improvement (IDI), which does not require categories, and instead accounts for the size of movements up and down [12]. Using the differences between integrals of sensitivities (IS) and integrals of “one minus specificities” (IP) for models with and without the new variable (i.e., new model vs. standard model), the IDI quantifies jointly the overall improvement in sensitivity and specificity over all possible cutoffs on the (0,1) interval. The IDI is defined as

IDI = (IS_new_ − IS_old_) − (IP_new_ − IP_old_),

where IS_new_ is the integrated sensitivity over all possible cutoffs for the new model (standard plus a new added variable) and IP_new_ is the integrated “one minus specificity” over all possible cutoffs for the new model. IS_old_ and IP_old_ are the same quantities for the standard model. Thus, the IDI is the difference between improvement in average sensitivity and any potential increase in average “one minus specificity”.

It has been shown that to estimate IDI using sample data we can use the following equation:

$$\hat{IDI}=({\bar{\hat{p}}}_{new,events}-{\bar{\hat{p}}}_{old,events})-({\bar{\hat{p}}}_{new,nonevents}-{\bar{\hat{p}}}_{old,nonevents}),$$

where

$${\bar{\hat{p}}}_{events}=\frac{\sum_{i\;in\;events}{\hat{p}}_{i}}{\#\;events}$$

is the mean of the model-based predicted probabilities of the event for those who develop the event and

$${\bar{\hat{p}}}_{nonevents}=\frac{\sum_{j\;in\;nonevents}{\hat{p}}_{j}}{\#\;nonevents}$$

is the mean of the model-based predicted probabilities of the event for those who do not have the event. These quantities are estimated for both the standard (old) model and the added variable (new) model.

The standard error can be estimated as

$$SE\left(\hat{IDI}\right)=\sqrt{{\left({\hat{SE}}_{events}\right)}^{2}+{\left({\hat{SE}}_{nonevents}\right)}^{2}}$$

where ${\hat{SE}}_{events}$ is the standard error of paired differences in new and old model-based predicted probabilities among those with the event and ${\hat{SE}}_{nonevents}$ is the corresponding standard error among those without the event [12].

In the extension of IDI for survival analysis, IS(t) is the average sensitivity at time t and IP(t) is the average (1-specificity) at time t. Chambless et al. show that

$$IS\left(t\right)-IP\left(t\right)=R^{2}(t)=\frac{Var[S\left(t|Z\right)]}{S\left(t\right)\cdot [1-S\left(t\right)]}$$

where S(t|Z) is the survival function, S(t) = E[S(t|Z)], and S(t)[1-S(t)] is the variance of the binomial variable ‘event have time t’ [16]. This ratio is interpreted as the proportion of variance explained by the model and the authors also show that R^2^(t) is bounded on the interval [0, 1]. This quantity can be estimated from the fitted survival function, such that

$${\hat{R}}^{2}(t)=\frac{\hat{Var}[S\left(t|Z\right)]}{\hat{S}\left(t\right)\cdot [1-\hat{S}\left(t\right)]}$$

where $\hat{S}\left(t\right)$ is obtained through Kaplan–Meier estimates. By rearranging the definition of IDI presented by M. Pencina et al. such that

IDI = (IS_new_ − IP_new_) − (IS_old_ − IP_old_),

we can see the following holds:

IDI(t) = R^2^(t)_new_ − R^2^(t)_old_,

or the difference in the proportion of variance explained by the new and old models [12,16]. We can estimate IDI(t) by taking the difference between the estimators for R^2^(t)_new_ and R^2^(t)_old_.

### 2.4. Confidence Interval Estimation

Various methods for confidence interval (CI) estimation exist. The most common estimation methods are intervals derived under normal theory assumptions, or asymptotic CIs [29]. We assume there is a one-sample situation with sample data obtained by random sampling from an unknown distribution *F*, where *F →* **x** = (x_1_, x_2_, …, x_n_). Suppose we have a parameter of interest *θ* such that *θ = t(F)*. Let $\hat{\theta }$ = *t(*$\hat{F}$*)* be the plug-in estimate of *θ* and let $\hat{se}$ be a reasonable estimate of the standard error for $\hat{\theta }$. In most circumstances, as the sample size *n* increases and grows large, the distribution of $\hat{\theta }$ converges to the normal distribution:

$$Z=\frac{\hat{\theta }-\theta }{\hat{se}}∼N\left(0,1\right)$$

Let $z^{\left(\alpha \right)}$ indicate the 100*α* quantile and $z^{\left(1-\alpha \right)}$ indicate the 100(1 − α) quantile of a N(0,1) distribution. The asymptotic CI with coverage probability equal to 1 − 2*α* (where *α* is equal to the significance level) for a parameter *θ* is given by:

$$\left[\hat{\theta }-z^{\left(1-\alpha \right)}\cdot \hat{se},\;\hat{\theta }+z^{\left(1-\alpha \right)}\cdot \hat{se}\;\right].$$

*SE* represents the standard error and *z* is the critical value from the standard normal distribution [29].

Alternative methods for estimating CIs that do not employ asymptotic theory are CIs estimated with bootstrap techniques. The bootstrap is a data-based simulation method used for statistical inference [29]. The bootstrap distribution of a parameter estimator can be used to calculate a variety of 100(1 − α)% CIs without having to make normal theory assumptions [29]. We explore several types of CIs based on the bootstrap distribution: bootstrap-t, percentile, and bias-corrected percentile (BC), as well as hybrid and bias-corrected and accelerated percentile (BCa) in limited scenarios [29,30,31].

## 3. Simulation Study Results

For simulations utilizing logistic regression, we defined a vector X of five predictor variables conditional on Y following a multivariate normal distribution such that for nonevents X|Y = 0~N(µ_0_, Σ) and for events X|Y = 1~N(µ_1_, Σ), µ_0_ = (0.0, 0.0, 0.0, 0.0, 0.0) and µ_1_ = (0.7, 0.0, 0.2, 0.5, 0.8) respectfully. Thus, both x_1_ and x_5_ are strong predictors of the risk of Y, x_2_ is a null predictor, x_3_ is a weak predictor, and x_4_ is a moderate predictor. We assumed that x_1_ is the standard predictor and the four other predictor variables to be new candidate markers. Without loss of generality, we assumed that **Σ** is the identity matrix with order 5 × 5, which ensures independence among the predictors conditional on event status. We considered event rates of 10% and 50%. We assumed no missing data among N observations and that all N observations were independent. We employed logistic regression to estimate model-based predicted probabilities of the event Y using a function of linear combinations of the predictors described above. We defined the standard model as predicting the risk of event Y from x_1_ only, resulting in a baseline AUC close to 0.7. We further defined four updated models, each predicting the risk of event Y from x_1_ plus an additional predictor. For each model, we estimated predicted probabilities of event Y for each observation to estimate the measures of prediction increment quantifying the improvement of the new risk prediction models: ∆AUC, NRI, NRI > 0, and IDI. We considered NRI versions with two and three categories. For 2-category NRI (2catNRI), we chose the event rate as the category cutoff. For 3-category NRI (3catNRI), we used the categories [0.00, 0.05), [0.05, 0.20), and [0.20+) for 10% event rate and [0.00, 0.40), [0.40, 0.60), and [0.60+) for 50% event rate [28].

For the survival analysis, we considered two incidence rates, 10% and 50% (with 90% and 50% expected survival, respectively), over a 10-year follow-up period. We used two different censoring distributions: (1) Type I censoring (time-terminated) and (2) Type II (random censoring; failure-terminated) [8]. We simulated random censoring with the uniform distribution. Failure time assumed a Weibull distribution with shape parameter = 2. The scale parameter varied depending on the target incidence rate and effect size for each model. We defined a vector of five continuous, uncorrelated predictor variables following a multivariate normal distribution, with effect sizes of strong to null. For predictor variables x_1_, x_2_, x_3_, x_4_, and x_5_, hazard ratio (HR) values were 2.0, 1.0, 1.2, 1.5, and 2.0, respectively. We assumed x_1_ to be the established standard predictor, while the four remaining predictors were the candidate variables for model inclusion consideration. We considered the following four comparisons of the standard model (HR = 2.0): (1) standard (HR = 2.0) + null (HR = 1.0), (2) standard (HR = 2.0) + weak (HR = 1.2), (3) standard (HR = 2.0) + moderate (HR = 1.5), (4) standard (HR = 2.0) + strong (HR = 2.0). We assumed that the event status, failure time (or censoring time), and predictor variables were available for all N observations, as well as independence among observations. After assessing validity of the PH assumption, we employed Cox PH regression to estimate model-based predicted probabilities based on the models described above; for each comparison, we estimated the predicted probability according to the standard model and the predicted probability according to the added variable model. Using these pairs of predicted probabilities, we estimated five measures of prediction increment quantifying the improvement of the new risk prediction models: ∆C, 2catNRI, 3catNRI, NRI > 0, and IDI. We applied the same categories for 2catNRI and 3catNRI that were used in the logistic regression simulations [28].

For both logistic regression and survival analysis, we used n = 100,000 (with 1000 iterations) to construct empirical distributions and samples sizes of n = 2000 and n = 300 to construct bootstrapped 95% CIs with 2000 replications. We repeated the sampling 1000 times to assess coverage probabilities. Since derivations for standard error estimates were not previously provided in the survival analysis extensions, we omitted estimation of CIs assuming asymptotic theory in the survival analysis framework. Data simulation and analyses were completed using SAS software, version 9.4 (Copyright 2002–2012 SAS Institute Inc., Cary, NC, USA). We performed bootstrap analysis with the SAS macro jackboot.sas at http://support.sas.com/kb/24982.html (accessed on 15 October 2016).

**Figure 1:**
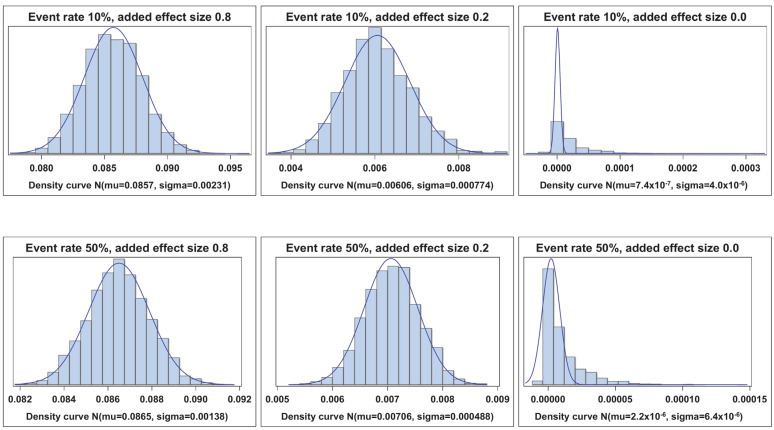
Empirical distribution of ∆AUC. Visual representation of the empirical distribution as the added marker effect moves closer to the null.

**Figure 2:**
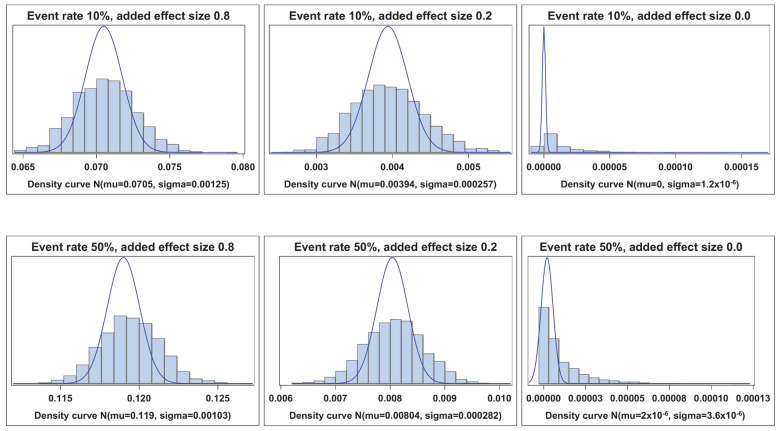
Empirical distribution of IDI. Visual representation of the empirical distribution as the added marker effect moves closer to the null.

**Figure 3:**
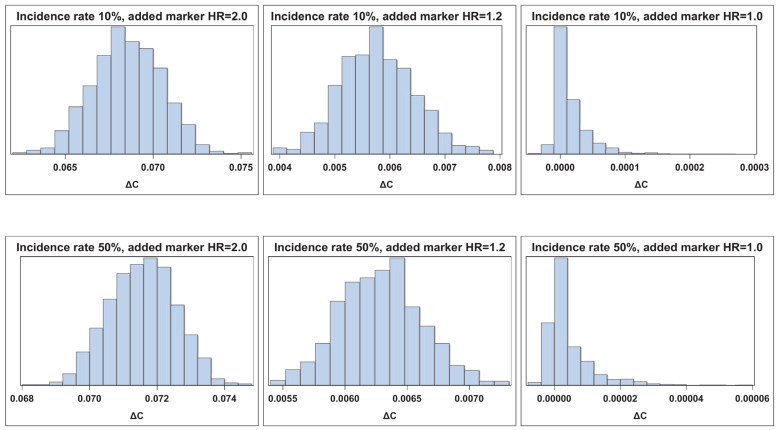
Empirical distribution of ∆C assuming Type I censoring. Visual representation of the empirical distribution as the added marker effect moves closer to the null.

**Figure 4:**
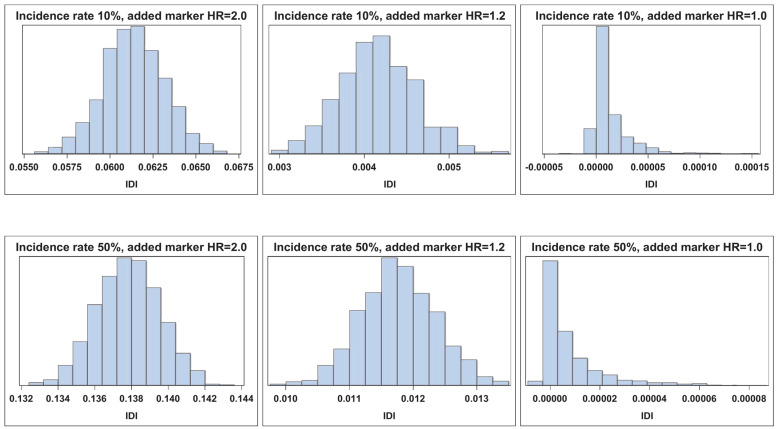
Empirical distribution of IDI assuming Type I censoring. Visual representation of the empirical distribution as the added marker effect moves closer to the null.

Figure 1, Figure 2, Figure 3 and Figure 4 illustrate the empirical distributions of ΔAUC, IDI, ∆C, and IDI(t) for three of the added-variable regression models. The empirical distributions of 3catNRI, 2catNRI, and NRI > 0 are in Supplemental Figures S1–S11. Each figure illustrates the empirical distribution as the added marker moves closer to the null. The added marker with moderate effect size is omitted, as there is very little change in the shape of distribution until the null added marker. The empirical distributions under the null hypothesis are right-skewed. The histograms for IDI under the logistic framework demonstrate that the standard error appears to be underestimated. To assess where the issues with degeneration began, we also looked at empirical distributions for added markers with effect sizes of 0.1 and 0.05. We observed no change in the shape of the empirical distributions as compared with those for the weak effect size.

Table 1 and Table 2 summarize coverage probabilities for considered scenarios in logistic regression. Defining acceptable coverage as 94–96%, acceptable coverage occurs for 26% of asymptotic intervals, 36% for BC intervals, 23% for percentile intervals, and 18% for bootstrap-t intervals. Asymptotic, BC, and bootstrap-t interval methods exhibit low coverage (percentage low: 66%, 57%, and 68%, respectively) whereas the percentile method provides excess coverage (73% high). Likewise, none of the prediction increment measures had at least 50% of scenarios with acceptable coverage: ∆AUC with 47%, 3catNRI and 2catNRI each with 9%, NRI > 0 with 44%, and IDI with 34%. Considering combinations of CI type and prediction measures, the BC method has the best performance for NRI > 0 (69% with acceptable coverage), percentile-based for IDI and ∆AUC (63% and 56%, respectively), and BC for IDI and ∆AUC (56% and 50%, respectively). Coverage was generally too low when adding a null marker, except for percentile CIs, and all CI types for ∆AUC, for which it was high. When adding a moderate or strong marker, most CI methods have appropriate coverage for ∆AUC. There is consistently low coverage by the asymptotic CIs for IDI and 3catNRI, exemplifying the potential underestimation of their standard errors. We also note that the bootstrap-t intervals perform consistently poorly for all versions of NRI, except for 3catNRI with the strongest added marker in the largest sample size. Finally, while the percentile intervals appear to exhibit ≥95% coverage in nearly all scenarios, we do note that this method does not seem to perform well for IDI with a null added marker in the 50% event rate situation; the bootstrap-t interval seems to be the better choice. We also considered hybrid bootstrap intervals and BCa intervals in limited scenarios, and the results are summarized in Supplemental Table S1. We did not observe adequate improvements in coverage with either interval type.

Table 3 and Table 4 summarize coverage probabilities of four bootstrapped CI types assuming Type I censoring. Acceptable coverage occurs for 31% of BC intervals, 30% of percentile intervals, 24% of bootstrap-t intervals, and 13% of hybrid intervals. BC, bootstrap-t, and hybrid intervals tend to exhibit low coverage (60%, 63%, and 78% of intervals, respectively) while 68% of percentile intervals tend to have excess coverage. ∆C and IDI have the best chance of acceptable coverage (41%) while both categorical NRIs have the lowest percentages (8% for 3catNRI and 5% for 2catNRI). The BC method has the best performance for NRI > 0 (38% with acceptable coverage) and the percentile method and BC method are best for IDI and ∆C (57% and 50%, respectively, for both measures). In general, coverage is better as the adjusted HR of the added marker increases, with instances of excess or too low of coverage in the weak and null added marker scenarios. Similar results are observed assuming random censoring (Supplemental Tables S2 and S3). The hybrid intervals exhibit some unexpected behavior. The coverage of the hybrid intervals decreases as the adjusted HR of the added marker decreases, with a large increase in coverage under the null. The hybrid interval method adjusts for both bias and skewness, and this interval type has been shown to suffer from low coverage in unexpected situations [29]. In simulation, the expected coverage probability is not achieved under the alternative, and the method appears to over-correct for bias and skewness under the null. For Type I censoring, BC intervals, percentile intervals, and bootstrap-t intervals perform well for ∆C, NRI > 0, and IDI in scenarios with a moderate or strong added marker. For the 50% incidence rate, the hybrid interval also works well for these measures, but there is decreased coverage for the smaller sample size. Overall, issues with acceptable coverage exist for both 3catNRI and 2catNRI. For the null added marker, there are instances of acceptable coverage—most notably hybrid intervals for 3catNRI (assuming large sample size), BC intervals for NRI > 0 (assuming large sample size), and bootstrap-t for IDI (assuming small sample size). For random censoring, there are more noticeable issues with coverage. The BC intervals, percentile intervals, and bootstrap-t intervals still perform well in the moderate to strong added marker scenarios, but there are gaps in good performance. The hybrid interval appears to be a good option for categorical NRI in the null added marker case. Finally, the percentile interval had ≥95% coverage in most scenarios.

Due to the results of the empirical distributions and CI coverage probability analysis, we evaluated bias. Some of the bootstrap methods correct for bias, while others may amplify bias in situations where bias exists and is not properly accounted for. For example, the percentile method performs well for unbiased statistics, but may not perform well when bias is present or for asymmetric sampling distributions. In contrast, the BC method corrects for median bias, not mean bias. Bias of a point estimator $\hat{\theta }$ for a parameter *θ* is the difference between the expected value of $\hat{\theta }$ and *θ*; that is [32]:

$${Bias}_{\theta }\hat{\theta }=E_{\theta }\hat{\theta }-\theta$$

Thus, positive bias exists when the sample estimate is larger than the population estimate and negative bias exists when the opposite holds. Supplementary Figures S12–S23 show boxplots of bias estimates for all simulated scenarios under both the logistic and survival frameworks. The reference line represents zero bias. In general, the bias distributions are skewed to the right. In the logistic framework, variability in bias increases as the effect size increases for all five measures, with NRI > 0 having the largest variability overall. We also see positive bias with larger magnitudes for all measures in the smaller sample size scenarios. In the survival framework, we observe mean bias of zero for the strongest added markers, with positive bias as the adjusted hazard ratio of the added marker decreases towards the null.

Regarding the CI coverage probabilities, the percentile CIs exhibit coverage of more than 95%, while the other methods yield a coverage too low in more than half of scenarios. Potential issues arise for both coverage extremes. Coverage probabilities below 95% indicate that CIs are too narrow and that Type-I error may be inflated. Coverage probabilities substantially above 95% indicate conservative inference and may be wider than expected. We explored the relationship between the observed coverage probabilities and the widths of the CIs. We constructed boxplots demonstrating the distributions of CI widths. The data for the sample size of 300 are presented in Supplemental Figures S24–S38. Overall, the excess coverage of percentile CIs has not been achieved at the expense of their width. Their width is very similar to the width of the bootstrap BC intervals, yet with better coverage. The asymptotic CIs have the lowest width, but this is also reflected in their low coverage probabilities, further evidence of the potential underestimation of the standard error for some metrics. The bootstrap-t intervals not only demonstrate the most variability in interval width, but they also have consistently poor coverage except with strong added markers in large sample sizes. Additionally, we see an increasing trend in the width of the CIs as the effect size of the added marker increases, with this trend appearing to be most apparent for ∆AUC and IDI. This trend is not present for NRI > 0. It appears that, in general, while the percentile intervals frequently had more than 95% coverage, the widths were shorter when compared with the other CIs we examined. If investigators are looking for a “one size fits all” interval method, the percentile bootstrap interval may be an appropriate choice, as this interval performed the strongest in simulation without exhibiting excess width as compared with the other CI methods. This recommendation requires bootstrapping computations, which can easily be performed in SAS or R. The percentile interval method takes some of the least amount of extra time as compared with the other bootstrap methods. We also observed that the BC method works well for ∆AUC, 3catNRI, jsNRI, NRI > 0, and IDI in situations with a strong added marker and some scenarios with a moderate added marker, depending on event rate and sample size. For CI and standard error estimation from bootstrapping, we recommend that a sufficient number of bootstrap resamples is used; we used B = 2000 for 95% CIs. The number of bootstrap resamples required increases as the desired confidence level increases, which also increases computation time if an interval with more that 95% confidence is desired. However, if the investigator has limited time and wishes to use the asymptotic CI formulas, these formulas would best be used in cases with at least moderate added effect size and with using bootstrap-estimated standard errors, most importantly for IDI and NRI as the asymptotic standard error formulas for these metrics produce CIs with low coverage.

## 4. Conclusions

Discrimination improvement is an important metric to estimate when developing prediction models for assessing cancer risk and establishing new risk factors in oncology research. We summarized several measures used for estimating discrimination improvement: ΔAUC, NRI, NRI > 0, and IDI. We also discussed motivations for their development, definitions, and potential issues with their estimation and use. The motivation for this study was to improve the estimation of these measures, which was studied through simulations targeting confidence interval estimation. The simulation studies performed demonstrate that the assumed normal empirical distributions of these measures break down under the null hypothesis with varying levels of degeneration. This is problematic in estimation of confidence intervals assuming normal theory, which all of these measures assume. Additionally, the asymptotic standard errors for NRI and IDI under the logistic framework appear to be consistently underestimated, decreasing the coverage probability for the asymptotic confidence intervals in nearly all studied scenarios. The primary finding in both the logistic framework and the survival analysis framework is that the percentile CIs performed well regarding coverage, without compromise to their width; this finding was robust in most scenarios. For the simplest recommendation, we suggest the use of the percentile bootstrap interval with enough bootstrap resamples B, with increased B for larger confidence levels of (1 − α)%. The percentile interval method takes the least amount of extra computing time as compared with the other bootstrap methods, and it can be easily estimated with use of SAS, R, or other statistical computing software. However, if investigators wish to apply other bootstrap CI methods, there are other options depending on the desired performance metric, marker strength, incidence rate, and sample size.

There are limitations to this work that should be addressed in future research. While we currently recommend the percentile CIs as the method to use over intervals assuming normal therapy, we acknowledge that further methodological refinements should be considered to possibly enhance the performance of the bootstrapped methods, including a more comprehensive study of bias. We also plan to study the Jackknife technique, acknowledging that it may perform better for small data samples. Additionally, one strength of this work is that we assessed a wide range of possible scenarios to study CI performance in extreme cases. However, we acknowledge that additional simulations should be considered in future work. These should include additional sample size options, other incidence rates, risk models with more predictor variables included, correlated predictors, other censoring distributions, and models accounting for competing risks. We also acknowledge that these recommendations should be tested and validated in real data; at the time writing, we have applied for the use of specific clinical trial data to address this.

## Figures and Tables

**Table 1:** Coverage probabilities for 95% confidence intervals with 10% event rate in logistic framework.

	n = 2000									
	Strong New Marker (μ_Y=1_ = 0.8)					Moderate New Marker (μ_Y=1_ = 0.5)				
	∆AUC	3catNRI	2catNRI	NRI > 0	IDI	∆AUC	3catNRI	2catNRI	NRI > 0	IDI
**Asymptotic**	93.7	84.4	93.4	94.4	76.1	94.3	81.2	95.1	94.5	75.2
**Bias Corrected**	94.6	94.5	91.0	94.6	95.5	94.8	92.8	92.4	94.7	94.2
**Percentile**	94.7	97.0	97.5	95.5	95.4	94.7	97.4	99.1	96.2	94.0
**Bootstrap-t**	95.1	94.4	89.3	93.7	95.5	95.3	90.7	89.8	94.1	96.1
	**Weak New Marker (μ_Y=1_ = 0.2)**					**Null New Marker (μ_Y=1_ = 0.0)**				
	∆AUC	3catNRI	2catNRI	NRI > 0	IDI	∆AUC	3catNRI	2catNRI	NRI > 0	IDI
**Asymptotic**	90.6	86.4	93.5	95.4	71.0	100.0	94.6	94.1	95.8	94.0
**Bias Corrected**	93.3	88.0	87.6	93.8	92.7	98.4	90.5	89.7	96.0	95.5
**Percentile**	95.5	99.5	99.4	97.7	94.8	98.9	100.0	100.0	98.4	95.9
**Bootstrap-t**	87.4	84.8	83.6	88.7	87.9	98.6	88.1	86.0	90.0	98.4
	**n = 300**									
	**Strong New Marker (μ_Y=1_ = 0.8)**					**Moderate New Marker (μ_Y=1_ = 0.5)**				
	∆AUC	3catNRI	2catNRI	NRI > 0	IDI	∆AUC	3catNRI	2catNRI	NRI > 0	IDI
**Asymptotic**	92.6	80.9	93.3	94.2	76.4	88.7	78.6	92.2	93.5	71.8
**Bias Corrected**	94.3	91.2	91.8	95.3	95.4	92.3	87.1	87.0	92.3	92.9
**Percentile**	94.4	97.6	99.2	97.2	94.9	95.9	99.1	99.7	98.4	94.8
**Bootstrap-t**	96.0	88.8	87.5	93.6	97.3	86.4	82.8	83.0	87.3	90.5
	**Weak New Marker (μ_Y=1_ = 0.2)**					**Null New Marker (μ_Y=1_ = 0.0)**				
	∆AUC	3catNRI	2catNRI	NRI > 0	IDI	∆AUC	3catNRI	2catNRI	NRI > 0	IDI
**Asymptotic**	88.4	79.1	91.8	96.4	75.3	99.5	89.9	93.2	93.9	96.8
**Bias Corrected**	91.0	83.1	85.5	89.7	85.6	98.4	88.0	87.4	94.6	94.5
**Percentile**	97.1	99.9	100.0	97.7	97.3	98.8	100.0	100.0	98.4	95.2
**Bootstrap-t**	86.1	85.0	82.8	82.2	73.3	96.9	89.7	86.7	89.0	97.8
3catNRI categories: [0.00, 0.05), [0.05, 0.20), and [0.20+).										
2catNRI categories: [0.00, 0.10) and [0.10+).										

**Table 2:** Coverage probabilities for 95% confidence intervals with 50% event rate in logistic framework.

	n = 2000									
	Strong New Marker (μ_Y=1_ = 0.8)					Moderate New Marker (μ_Y=1_ = 0.5)				
	∆AUC	3catNRI	2catNRI	NRI > 0	IDI	∆AUC	3catNRI	2catNRI	NRI > 0	IDI
**Asymptotic**	95.5	89.7	93.8	95.7	70.4	94.5	90.4	93.9	93.6	69.9
**Bias Corrected**	95.5	94.2	94.2	96.3	96.0	94.2	93.6	93.0	94.4	94.4
**Percentile**	95.5	96.3	96.9	96.4	96.1	93.9	97.1	96.8	95.7	94.3
**Bootstrap-t**	95.5	93.4	93.4	94.5	96.6	94.6	91.7	91.2	94.5	95.2
	**Weak New Marker (μ_Y=1_ = 0.2)**					**Null New Marker (μ_Y=1_ = 0.0)**				
	∆AUC	3catNRI	2catNRI	NRI > 0	IDI	∆AUC	3catNRI	2catNRI	NRI > 0	IDI
**Asymptotic**	95.2	92.0	95.3	94.5	70.1	99.9	95.5	92.9	93.8	91.8
**Bias Corrected**	96.0	91.8	91.4	94.1	96.1	97.6	90.5	91.2	95.8	79.4
**Percentile**	95.4	99.6	99.3	96.1	95.8	97.8	100.0	100.0	98.0	78.4
**Bootstrap-t**	95.4	88.8	88.4	94.0	96.2	97.8	88.3	89.1	90.6	96.9
	**n = 300**									
	**Strong New Marker (μ_Y=1_ = 0.8)**					**Moderate New Marker (μ_Y=1_ = 0.5)**				
	∆AUC	3catNRI	2catNRI	NRI > 0	IDI	∆AUC	3catNRI	2catNRI	NRI > 0	IDI
**Asymptotic**	94.4	89.8	96.1	93.8	71.7	93.1	89.8	95.0	94.3	71.4
**Bias Corrected**	94.9	93.5	94.0	94.0	94.3	94.9	90.6	91.5	94.9	94.5
**Percentile**	95.0	98.4	98.7	95.9	93.8	95.2	98.2	99.1	96.5	94.8
**Bootstrap-t**	95.9	91.0	90.7	93.7	96.1	95.6	88.6	88.0	94.4	96.4
	**Weak New Marker (μ_Y=1_ = 0.2)**					**Null New Marker (μ_Y=1_ = 0.0)**				
	∆AUC	3catNRI	2catNRI	NRI > 0	IDI	∆AUC	3catNRI	2catNRI	NRI > 0	IDI
**Asymptotic**	86.6	90.0	93.3	96.3	68.4	99.9	94.8	92.2	94.1	93.6
**Bias Corrected**	89.9	86.8	89.8	90.8	87.0	98.2	90.2	92.1	94.3	76.9
**Percentile**	98.8	100.0	100.0	99.1	98.1	98.4	100.0	100.0	97.3	72.1
**Bootstrap-t**	78.7	82.1	85.0	84.4	77.3	98.6	86.9	89.0	89.0	97.9
3catNRI categories: [0.00, 0.40), [0.40, 0.60), and [0.60+).										
2catNRI categories: [0.00, 0.50) and [0.50+).										

**Table 3:** Coverage probabilities for 95% confidence intervals with 10% incidence rate and type I censoring in survival framework.

	n = 2000									
	Strong New Marker (Adjusted HR = 2.0)					Moderate New Marker (Adjusted HR = 1.5)				
	∆C	3catNRI	2catNRI	NRI > 0	IDI	∆C	3catNRI	2catNRI	NRI > 0	IDI
**Bias Corrected**	95.1	94.3	93.1	93.9	95.7	95.6	92.1	90.5	95.1	94.8
**Percentile**	95.2	96.9	98.4	95.5	95.8	95.3	97.5	98.4	96.5	94.8
**Bootstrap-t**	95.5	93.2	92.4	93.5	96.2	96.2	90.8	90.2	94.1	96.2
**Hybrid**	93.6	91.8	92.5	93.3	92.8	89.9	89.6	90.5	93.7	89.1
	**Weak New Marker (Adjusted HR = 1.2)**					**Null New Marker (Adjusted HR = 1.0)**				
	∆C	3catNRI	2catNRI	NRI > 0	IDI	∆C	3catNRI	2catNRI	NRI > 0	IDI
**Bias Corrected**	93.2	86.0	89.2	92.5	93.2	98.3	89.1	90.1	95.0	91.2
**Percentile**	96.9	99.6	99.9	97.6	95.2	98.8	100.0	100.0	98.2	92.0
**Bootstrap-t**	91.7	85.2	86.9	91.7	95.0	97.9	84.9	85.1	91.9	98.7
**Hybrid**	75.7	86.8	90.0	91.1	76.6	100.0	94.2	90.9	90.7	100.0
	**n = 300**									
	**Strong New Marker (Adjusted HR = 2.0)**					**Moderate New Marker (Adjusted HR = 1.5)**				
	∆C	3catNRI	2catNRI	NRI > 0	IDI	∆C	3catNRI	2catNRI	NRI > 0	IDI
**Bias Corrected**	94.3	90.4	91.0	92.6	94.3	87.9	85.4	90.0	91.7	91.6
**Percentile**	94.2	97.8	99.2	97.1	94.5	95.6	99.7	99.8	98.9	96.7
**Bootstrap-t**	95.3	90.9	89.1	93.1	95.2	87.3	80.5	85.5	90.0	92.1
**Hybrid**	83.6	84.7	88.2	90.4	81.3	77.8	81.5	88.9	88.3	73.1
	**Weak New Marker (Adjusted HR = 1.2)**					**Null New Marker (Adjusted HR = 1.0)**				
	∆C	3catNRI	2catNRI	NRI > 0	IDI	∆C	3catNRI	2catNRI	NRI > 0	IDI
**Bias Corrected**	92.6	82.5	89.1	89.2	84.5	98.5	85.7	84.3	93.4	84.8
**Percentile**	98.3	100.0	100.0	98.1	97.5	99.0	99.8	100.0	97.7	83.2
**Bootstrap-t**	82.1	73.3	79.0	87.3	82.2	96.9	78.1	77.9	90.8	95.2
**Hybrid**	92.4	91.0	87.9	86.5	66.3	99.7	93.3	84.1	89.6	99.7
3catNRI categories: [0.00, 0.05), [0.05, 0.20), and [0.20+).										
2catNRI categories: [0.00, 0.10) and [0.10+).										

**Table 4:** Coverage probabilities for 95% confidence intervals with 50% incidence rate and type I censoring in survival framework.

	n = 2000									
	Strong New Marker (Adjusted HR = 2.0)					Moderate New Marker (Adjusted HR = 1.5)				
	∆C	3catNRI	2catNRI	NRI > 0	IDI	∆C	3catNRI	2catNRI	NRI > 0	IDI
**Bias Corrected**	95.6	94.1	95.2	94.5	94.5	95.0	93.4	92.9	94.5	95.7
**Percentile**	95.9	96.0	97.0	94.9	94.6	94.6	96.9	97.3	96.5	95.3
**Bootstrap-t**	95.7	93.4	95.3	94.2	94.7	94.9	93.0	92.6	94.8	95.4
**Hybrid**	95.9	93.8	95.0	94.3	94.2	94.0	93.2	92.7	94.9	95.0
	**Weak New Marker (Adjusted HR = 1.2)**					**Null New Marker (Adjusted HR = 1.0)**				
	∆C	3catNRI	2catNRI	NRI > 0	IDI	∆C	3catNRI	2catNRI	NRI > 0	IDI
**Bias Corrected**	94.8	91.8	92.7	93.1	94.3	99.3	92.8	86.1	96.2	97.1
**Percentile**	94.8	98.2	99.1	94.9	94.4	99.4	100.0	100.0	98.5	97.9
**Bootstrap-t**	95.7	91.7	90.5	93.4	95.3	97.9	85.8	79.4	91.9	98.6
**Hybrid**	90.9	90.5	92.1	93.2	90.1	100.0	95.4	87.0	91.9	100.0
	**n = 300**									
	**Strong New Marker (Adjusted HR = 2.0)**					**Moderate New Marker (Adjusted HR = 1.5)**				
	∆C	3catNRI	2catNRI	NRI > 0	IDI	∆C	3catNRI	2catNRI	NRI > 0	IDI
**Bias Corrected**	95.2	92.7	92.8	94.9	95.0	95.4	92.5	93.4	93.9	94.8
**Percentile**	94.8	97.6	99.1	96.0	95.4	95.3	98.8	99.4	95.7	94.9
**Bootstrap-t**	95.1	92.9	91.6	94.5	95.2	95.8	92.5	91.9	93.9	96.3
**Hybrid**	93.3	92.0	91.8	94.1	92.6	88.3	91.3	92.3	93.6	88.5
	**Weak New Marker (Adjusted HR = 1.2)**					**Null New Marker (Adjusted HR = 1.0)**				
	∆C	3catNRI	2catNRI	NRI > 0	IDI	∆C	3catNRI	2catNRI	NRI > 0	IDI
**Bias Corrected**	90.4	87.0	91.4	92.8	90.4	99.4	88.7	76.2	95.0	96.9
**Percentile**	98.0	99.8	99.9	98.9	96.8	99.5	100.0	100.0	98.6	97.6
**Bootstrap-t**	86.8	85.3	86.2	91.7	90.3	97.9	77.0	67.7	91.5	98.2
**Hybrid**	80.2	89.6	89.3	91.4	73.1	99.9	93.6	74.2	91.2	100.0
3catNRI categories: [0.00, 0.40), [0.40, 0.60), and [0.60+).										
2catNRI categories: [0.00, 0.50) and [0.50+).										

## Data Availability

The original contributions presented in this study are included in the article/Supplementary Material. Further inquiries can be directed to the corresponding author.

## Supplementary Materials

Supporting information can be downloaded at: https://www.mdpi.com/article/10.3390/cancers17081259/s1.
