# Supplementary material for: Improving the Estimation of Prediction Increment Measures in Logistic and Survival Analysis
Source: Cancers (Basel). 2025 Apr 8;17(8):1259. doi: 10.3390/cancers17081259 (PMC12025450; doi:10.3390/cancers17081259)

Supplementary Materials

Supplementary Figure S1. Empirical distributions of 3catNRI

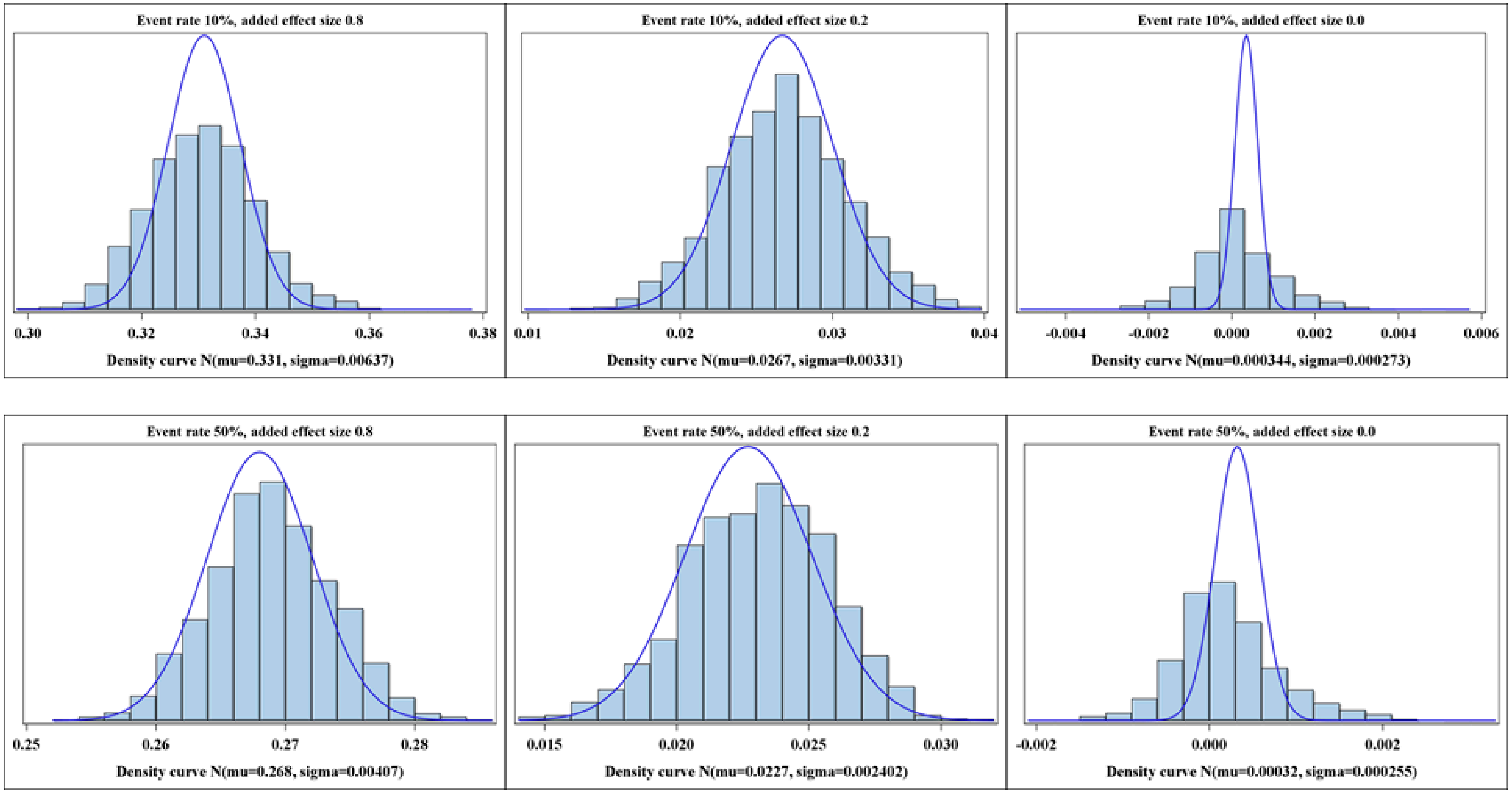

Supplementary Figure S2. Empirical distributions of 2catNRI

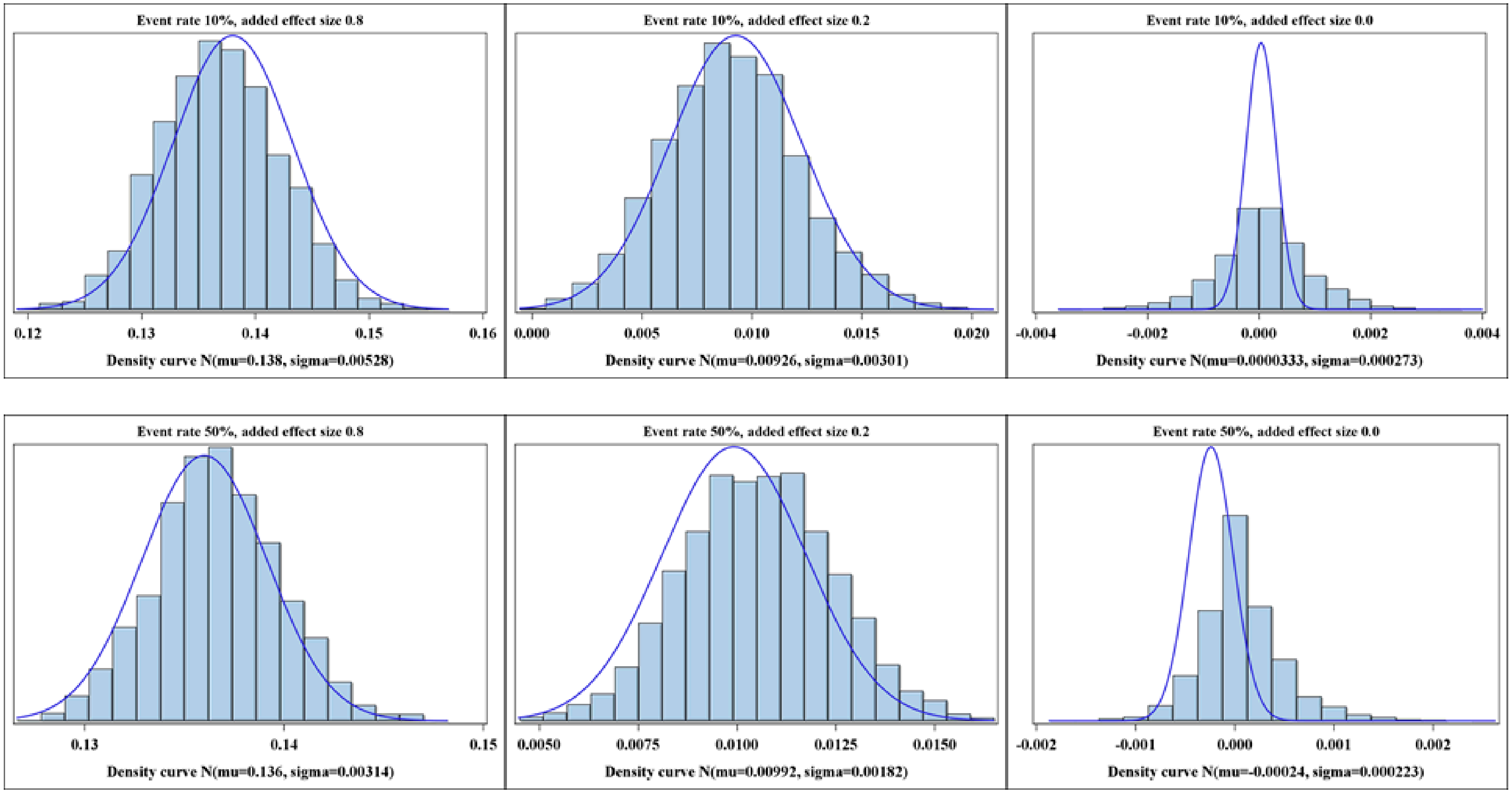

Supplementary Figure S3. Empirical distributions of NRI>0

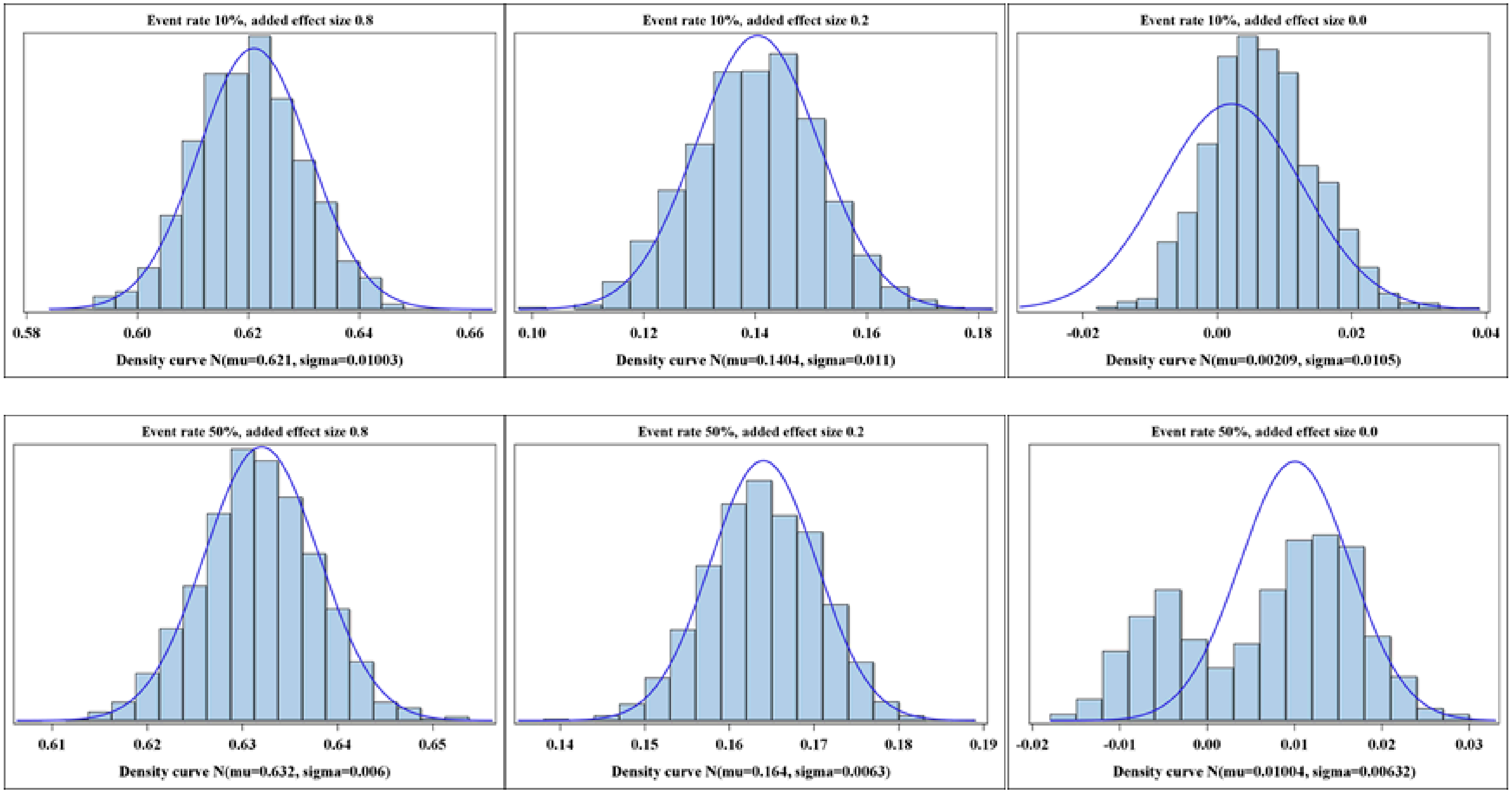

Supplementary Figure S4. Empirical distributions of 3catNRI assuming Type I censoring

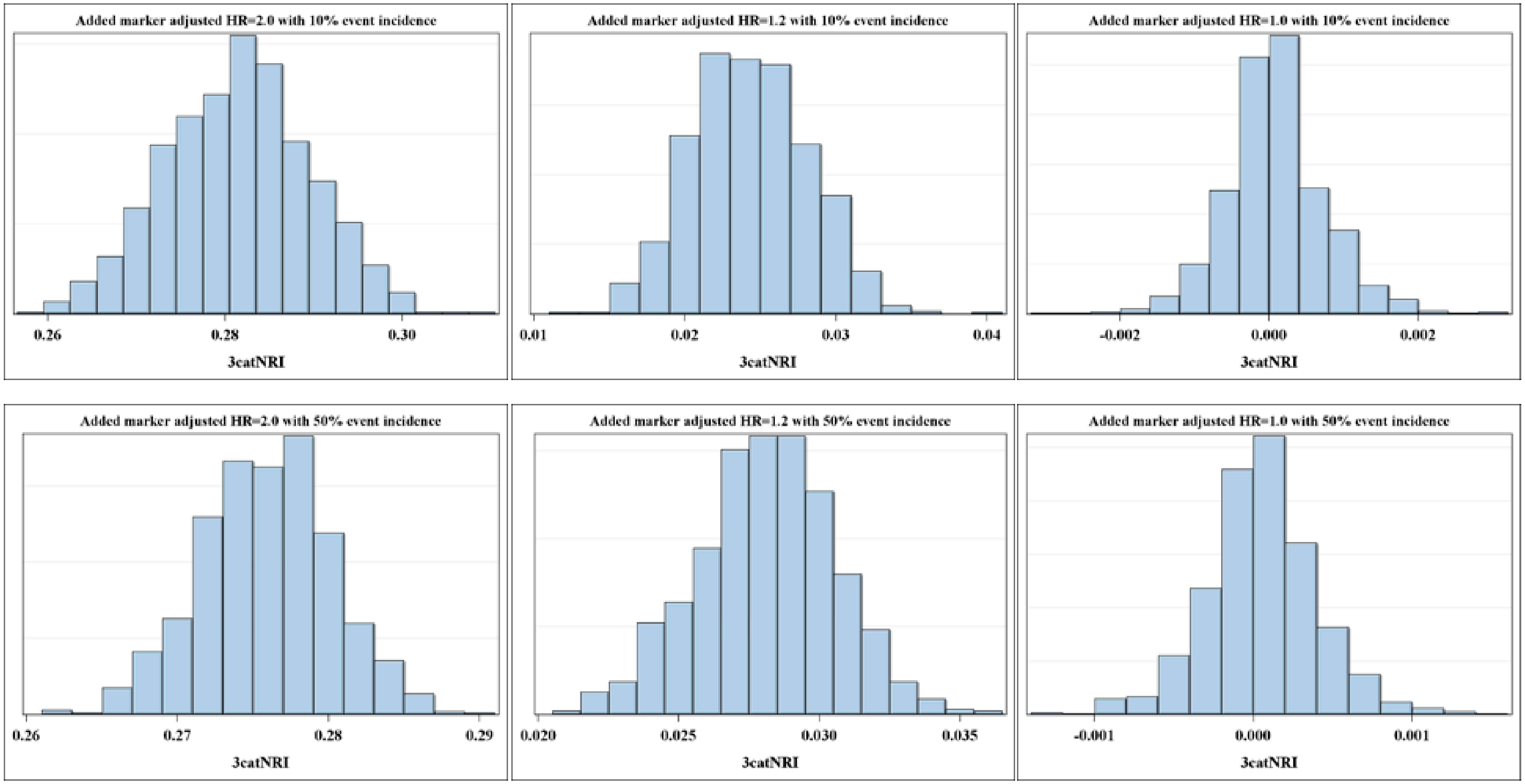

Supplementary Figure S5. Empirical distributions of 2catNRI assuming Type I censoring

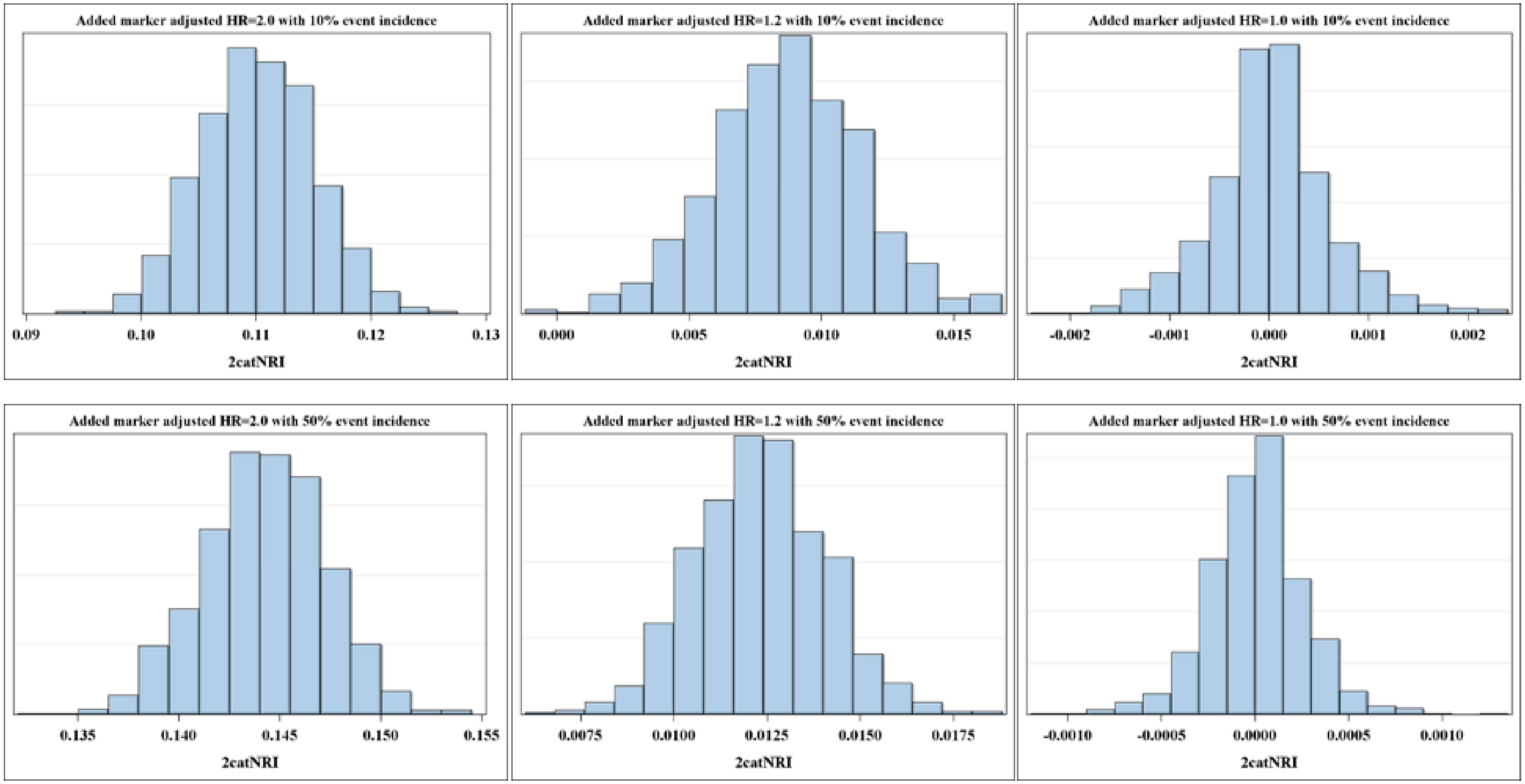

Supplementary Figure S6. Empirical distributions of NRI>0 assuming Type I censoring

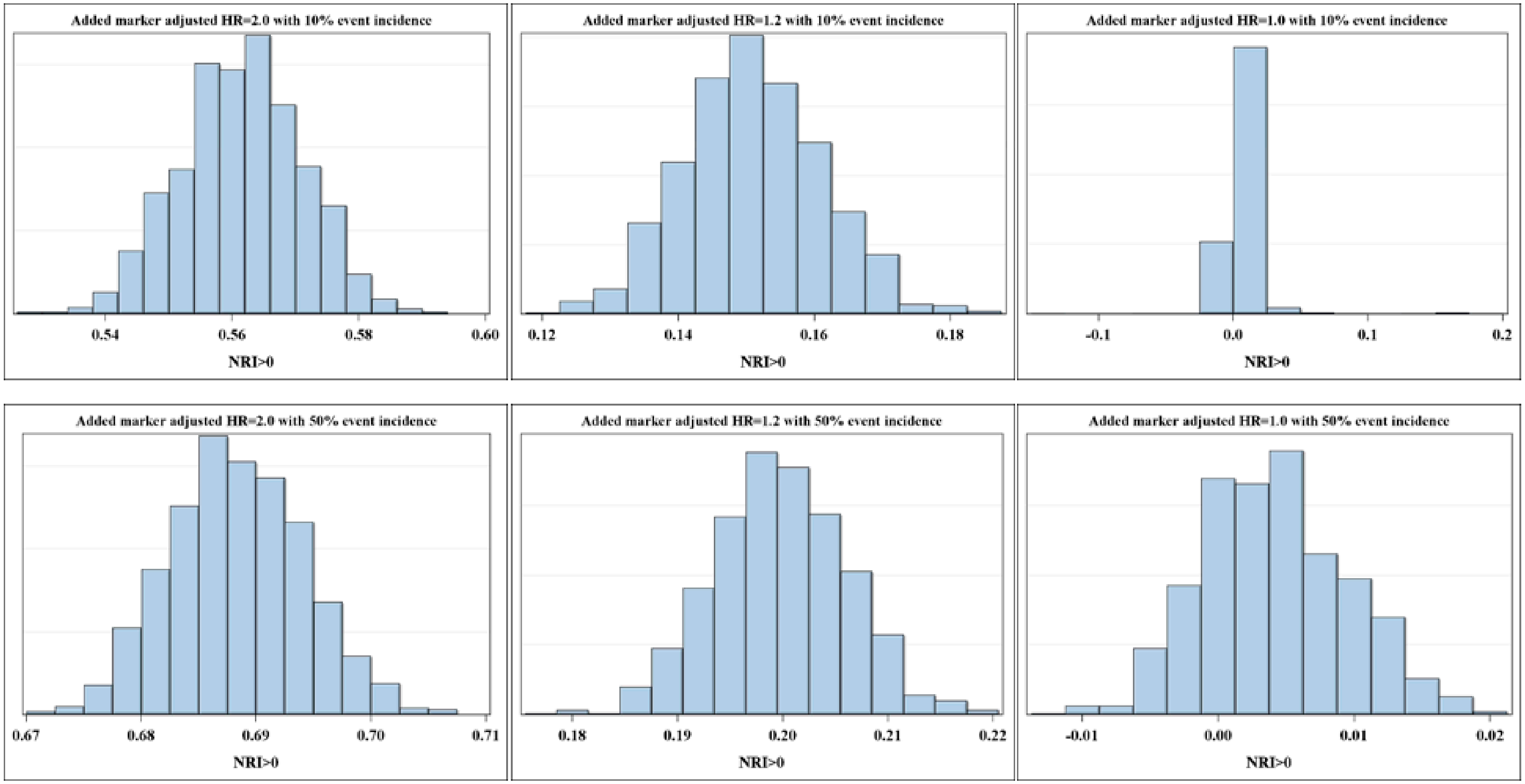

Supplementary Figure S7. Empirical distributions of ΔC assuming random censoring

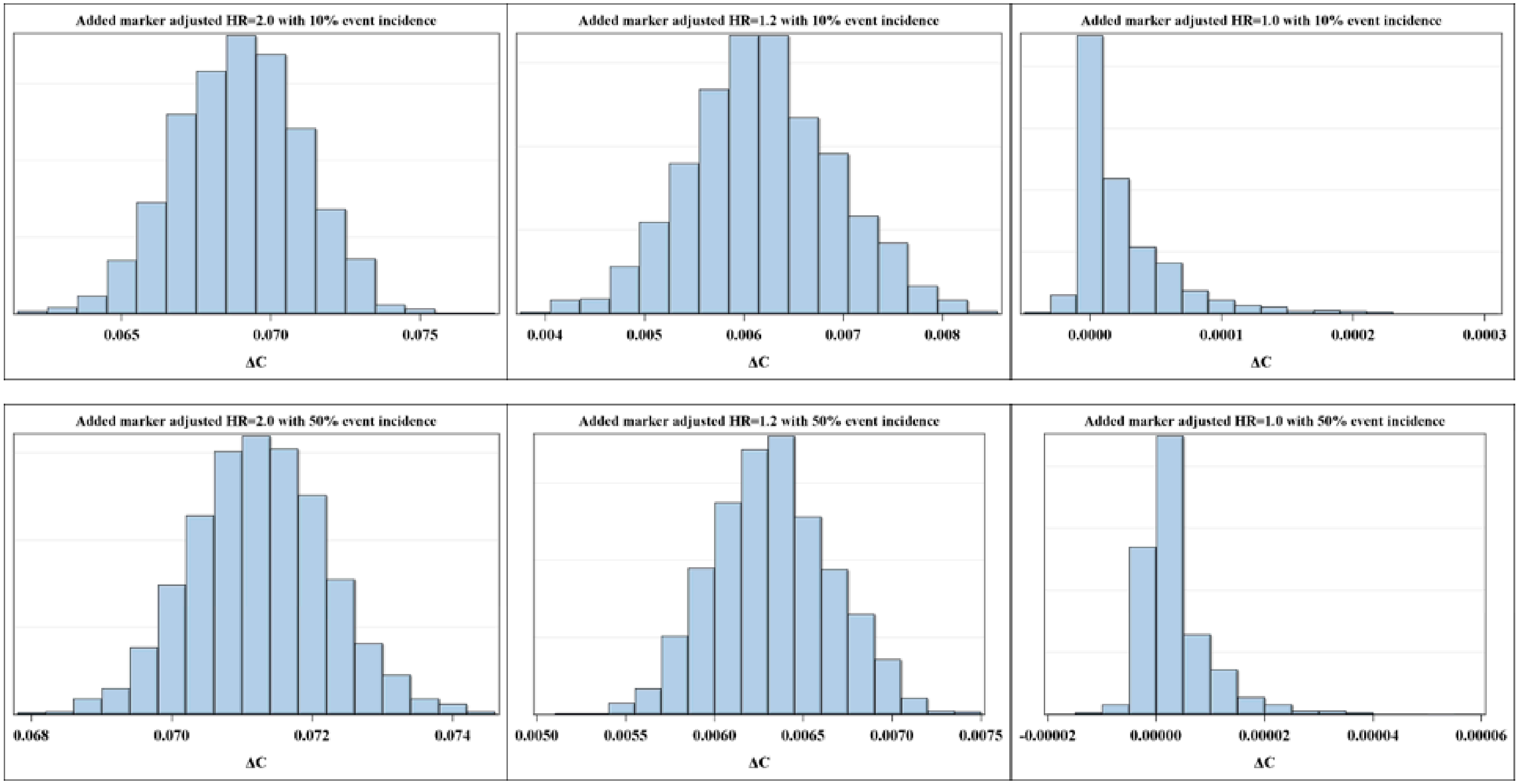

Supplementary Figure S8. Empirical distributions of 3catNRI assuming random censoring

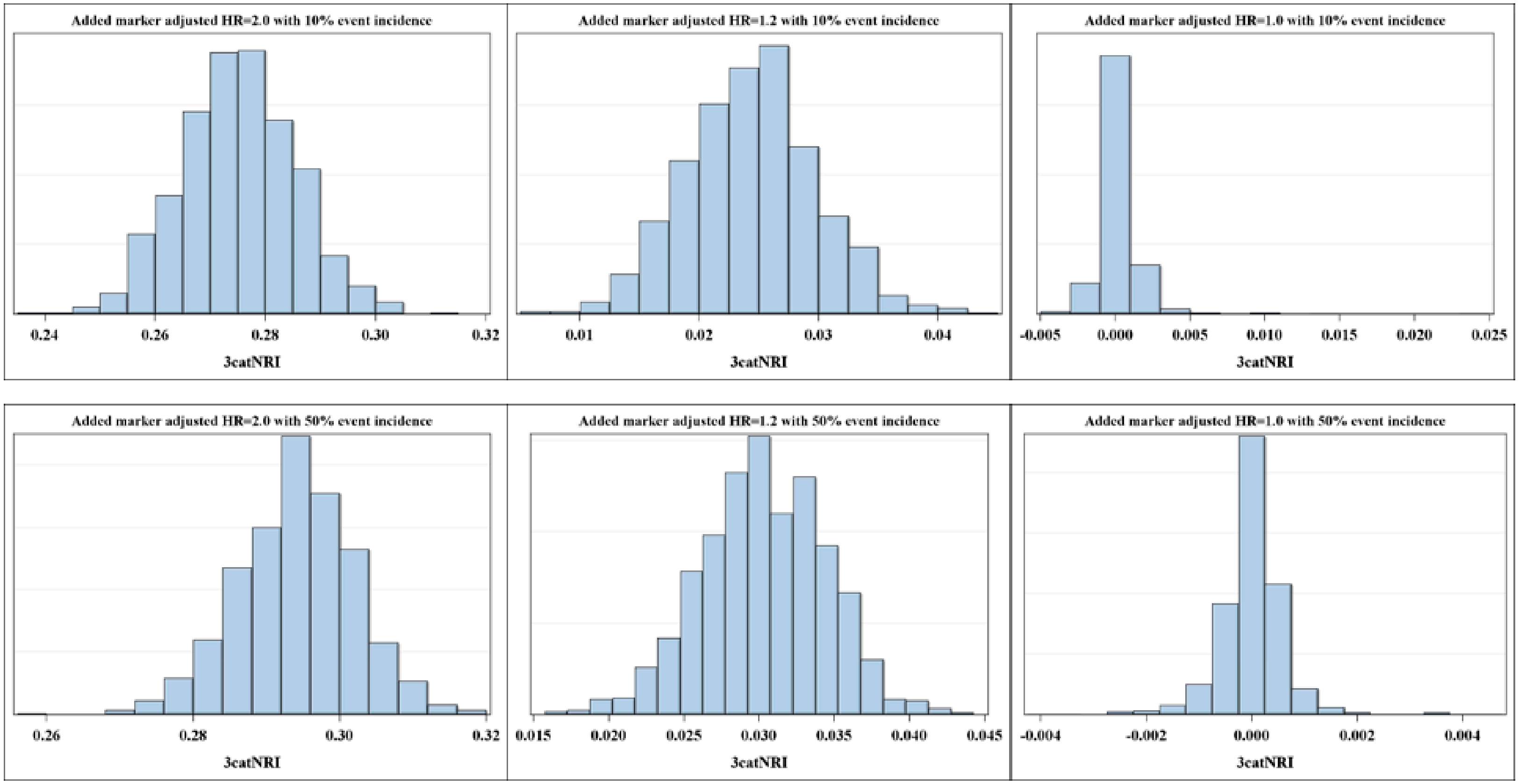

Supplementary Figure S9. Empirical distributions of 2catNRI assuming random censoring

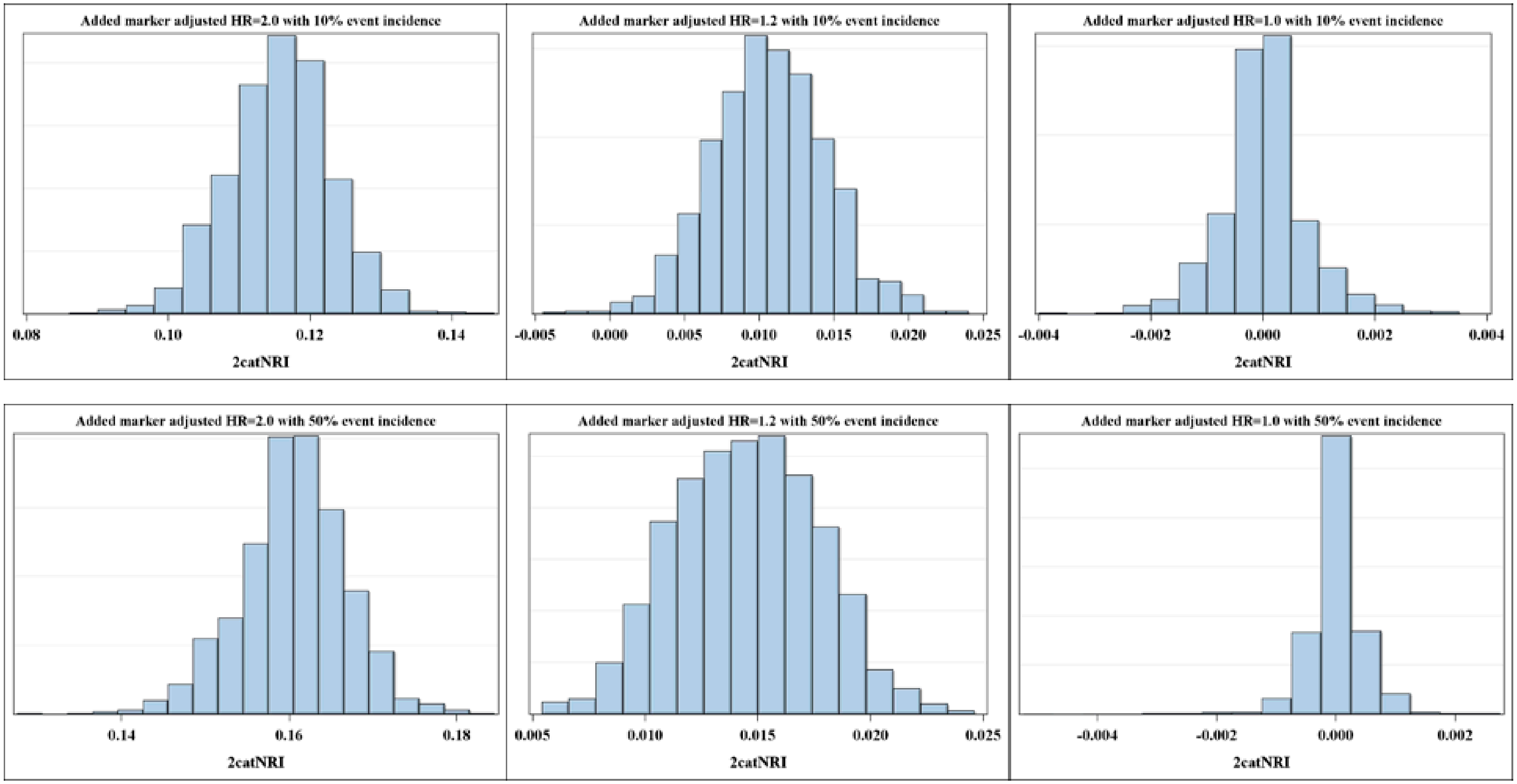

Supplementary Figure S10. Empirical distributions of NRI>0 assuming random censoring

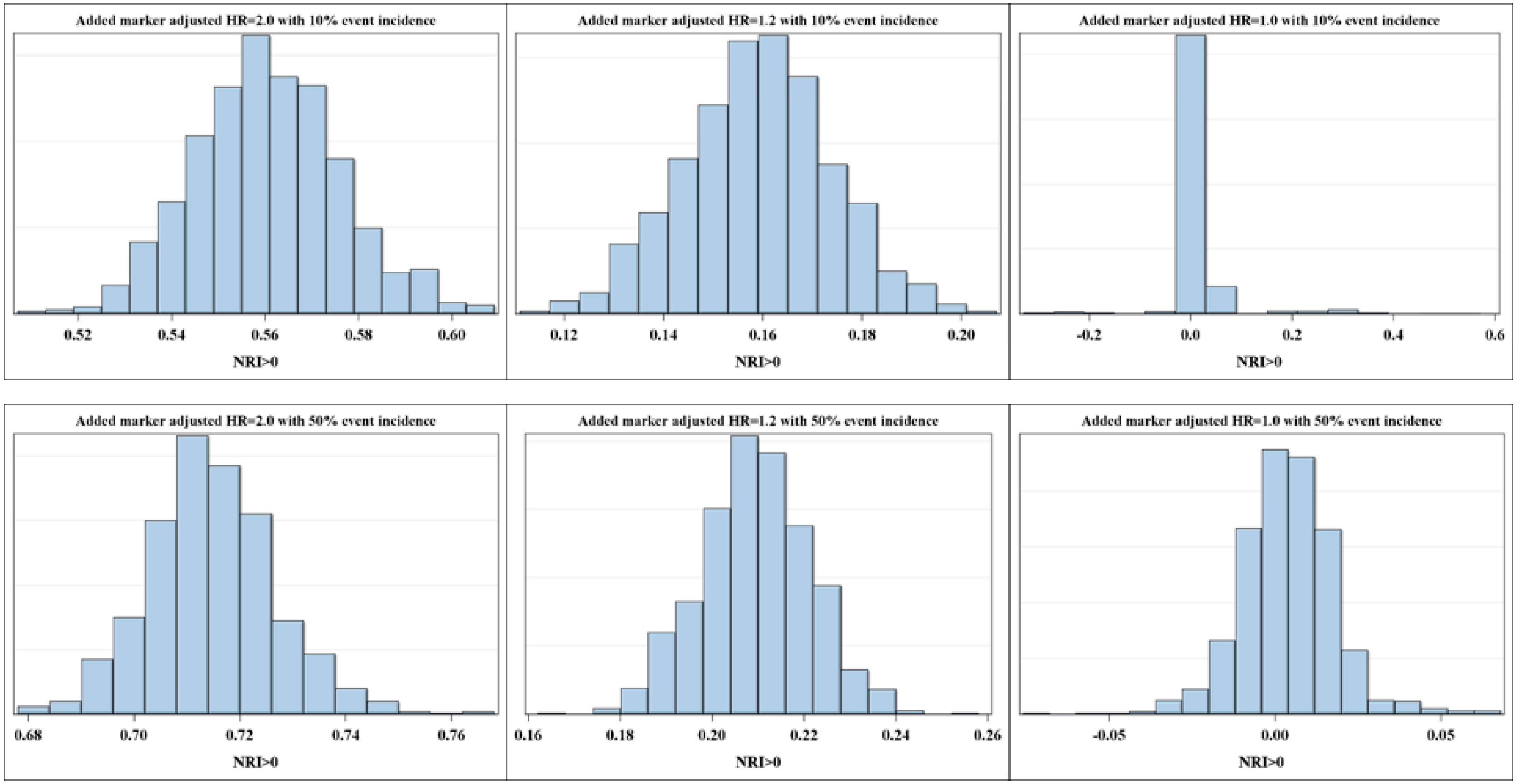

Supplementary Figure S11. Empirical distributions of IDI assuming random censoring

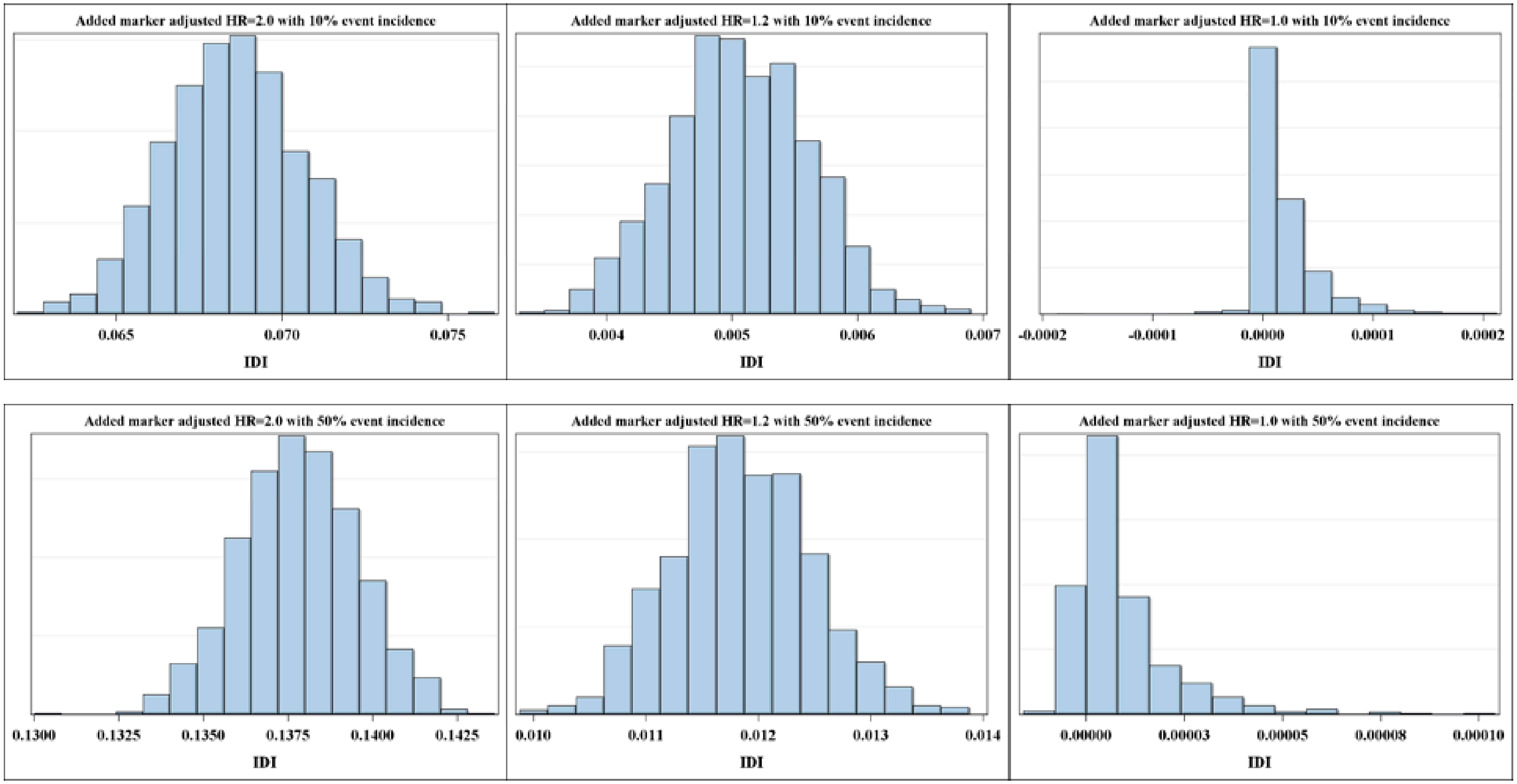

Supplementary Table S1. Coverage probabilities for hybrid intervals and bias-corrected and accelerated intervals in logistic framework

Event rate, sample size	Hybrid intervals									
	**Strong new marker ($\mu_{Y=1}$ = 0.8)**					**Moderate new marker ($\mu_{Y=1}$ = 0.5)**				
	ΔAUC	3catNRI	2catNRI	NRI>0	IDI	ΔAUC	3catNRI	2catNRI	NRI>0	IDI
**10%, n=2000**	93.0	93.5	89.8	93.8	93.5	90.4	89.9	91.8	94.3	90.0
**10%, n=300**	88.2	86.5	89.8	93.7	86.3	78.1	82.1	88.4	87.9	74.4
**50%, n=2000**	94.9	94.1	93.2	96.2	94.8	93.4	92.9	91.8	94.1	93.6
**50%, n=300**	91.1	92.0	92.3	93.7	92.0	86.6	88.4	90.4	94.0	87.6
	**Weak new marker ($\mu_{Y=1}$ = 0.2)**					**Null new marker ($\mu_{Y=1}$ = 0.0)**				
	ΔAUC	3catNRI	2catNRI	NRI>0	IDI	ΔAUC	3catNRI	2catNRI	NRI>0	IDI
**10%, n=2000**	79.0	88.4	90.4	91.9	76.7	100.0	94.9	94.1	92.3	100.0
**10%, n=300**	89.0	91.7	92.0	86.3	68.7	99.6	95.5	92.7	90.1	100.0
**50%, n=2000**	88.0	91.2	91	94.2	87.2	100.0	93.8	94	92.1	98.7
**50%, n=300**	75.7	88.5	92.2	88.3	68.6	99.7	92.7	94.8	90.2	99.0
	**BCa intervals**									
	**Strong new marker ($\mu_{Y=1}$ = 0.8)**					**Moderate new marker ($\mu_{Y=1}$ = 0.5)**				
	ΔAUC	3catNRI	2catNRI	NRI>0	IDI	ΔAUC	3catNRI	2catNRI	NRI>0	IDI
**10%, n=300**	93.5	91.2	91.4	95.3	95.3	91.1	86.5	86.2	92.3	92.8
**50%, n=300**	95.0	93.3	93.8	94.1	94.4	94.7	90.5	91.5	95.0	94.5
	**Weak new marker ($\mu_{Y=1}$ = 0.2)**					**Null new marker ($\mu_{Y=1}$ = 0.0)**				
	ΔAUC	3catNRI	2catNRI	NRI>0	IDI	ΔAUC	3catNRI	2catNRI	NRI>0	IDI
**10%, n=300**	90.1	82.7	84.9	89.0	84.7	97.7	87.2	86.7	94.1	94.6
**50%, n=300**	89.0	86.7	89.9	91.0	86.9	98.0	90.2	92.1	94.1	77.7

For 10% event rate, 3catNRI assumes categories [0.00, 0.05), [0.05, 0.20), and [0.20+).
For 10% event rate, 2catNRI assumes categories [0.00, 0.10) and [0.10+).
For 50% event rate, 3catNRI assumes categories [0.00, 0.40), [0.40, 0.60), and [0.60+).
For 50% event rate, 2catNRI assumes categories [0.00, 0.50) and [0.50+).

Supplementary Table S2. Coverage probabilities for 95% confidence intervals with 10% incidence rate and random censoring

	**n = 2,000**									
	**Strong new marker (adjusted HR=2.0)**					**Moderate new marker (adjusted HR=1.5)**				
	ΔC	3catNRI	2catNRI	NRI>0	IDI	ΔC	3catNRI	2catNRI	NRI>0	IDI
**Bias Corrected**	94.9	91.7	92.4	93.9	95.8	93.9	89.2	90.6	93.5	93.5
**Percentile**	94.7	97.0	97.8	94.8	95.9	93.9	97.5	99.5	95.7	93.6
**Bootstrap-t**	94.6	91.4	90.6	92.3	95.7	94.9	86.6	89.1	92.6	96.3
**Hybrid**	92.6	91.3	92.8	93.5	92.7	89.9	87.7	91.6	93.1	89.5
	**Weak new marker (adjusted HR=1.2)**					**Null new marker (adjusted HR=1.0)**				
	ΔC	3catNRI	2catNRI	NRI>0	IDI	ΔC	3catNRI	2catNRI	NRI>0	IDI
**Bias Corrected**	93.5	85.6	92.9	91.6	91.4	98.3	88.9	88.6	96.2	93.1
**Percentile**	95.7	99.9	99.5	97.2	93.7	98.8	99.9	100.0	99.2	94.1
**Bootstrap-t**	92.6	84.2	87.3	90.9	93.4	96.8	85.0	86.5	89.4	97.2
**Hybrid**	78.8	89.8	92.3	90.5	74.2	100.0	96.0	94.6	90.2	100.0
	**n = 300**									
	**Strong new marker (adjusted HR=2.0)**					**Moderate new marker (adjusted HR=1.5)**				
	ΔC	3catNRI	2catNRI	NRI>0	IDI	ΔC	3catNRI	2catNRI	NRI>0	IDI
**Bias Corrected**	94.3	90.2	92.3	92.8	94.4	90.9	82.9	90.5	92.5	91.4
**Percentile**	95.0	98.5	99.4	96.6	95.5	95.8	99.5	99.8	97.9	96.3
**Bootstrap-t**	95.3	89.6	86.8	92.5	96.0	89.3	79.4	79.5	90.3	91.5
**Hybrid**	85.1	86.3	88.5	90.5	82.1	78.3	83.4	88.2	88.7	70.1
	**Weak new marker (adjusted HR=1.2)**					**Null new marker (adjusted HR=1.0)**				
	ΔC	3catNRI	2catNRI	NRI>0	IDI	ΔC	3catNRI	2catNRI	NRI>0	IDI
**Bias Corrected**	93.1	81.2	85.1	91.6	84.8	98.4	85.9	81.9	94.0	89.0
**Percentile**	98.7	100.0	100.0	98.8	97.6	99.3	99.9	100.0	99.1	91.8
**Bootstrap-t**	82.2	73.5	86.2	87.4	80.9	97.1	80.6	87.0	89.0	95.9
**Hybrid**	94.0	91.1	95.3	85.7	71.5	99.8	95.2	94.5	87.6	99.9

3catNRI assumes categories [0.00, 0.05), [0.05, 0.20), and [0.20+).
2catNRI assumes categories [0.00, 0.10) and [0.10+).

Supplementary Table S3. Coverage probabilities for 95% confidence intervals with 50% incidence rate and random censoring

	n = 2,000									
	**Strong new marker (adjusted HR=2.0)**					**Moderate new marker (adjusted HR=1.5)**				
	ΔC	3catNRI	2catNRI	NRI>0	IDI	ΔC	3catNRI	2catNRI	NRI>0	IDI
**Bias Corrected**	95.6	92.7	94.7	93.9	94.6	96.1	91.1	91.7	94.6	94.0
**Percentile**	95.7	96.6	97.1	95.2	95.0	95.8	98.0	98.6	95.5	94.2
**Bootstrap-t**	95.5	92.1	93.3	92.7	94.4	96.1	91.1	91.5	92.8	93.7
**Hybrid**	95.2	93.8	95.1	96.3	94.3	94.8	92.3	92.6	96.1	92.9
	**Weak new marker (adjusted HR=1.2)**					**Null new marker (adjusted HR=1.0)**				
	ΔC	3catNRI	2catNRI	NRI>0	IDI	ΔC	3catNRI	2catNRI	NRI>0	IDI
**Bias Corrected**	96.0	88.6	89.5	92.6	95.7	99.0	88.2	86.2	98.0	97.6
**Percentile**	96.3	99.5	99.7	94.7	95.8	99.2	100.0	100.0	99.2	98.2
**Bootstrap-t**	96.1	88.8	89.2	92.2	95.6	98.0	82.8	86.6	90.0	97.9
**Hybrid**	89.5	90.7	90.7	95.1	88.7	100.0	93.7	93.8	93.7	100.0
	**n = 300**									
	**Strong new marker (adjusted HR=2.0)**					**Moderate new marker (adjusted HR=1.5)**				
	ΔC	3catNRI	2catNRI	NRI>0	IDI	ΔC	3catNRI	2catNRI	NRI>0	IDI
**Bias Corrected**	94.2	91.6	92.8	94.1	93.6	95.2	88.1	91.1	94.3	94.5
**Percentile**	94.4	98.5	98.8	97.9	94.1	95.6	99.6	99.6	97.4	94.9
**Bootstrap-t**	94.1	90.6	92.1	93.0	93.6	94.7	87.5	87.6	92.9	95.2
**Hybrid**	92.2	91.5	91.8	95.4	91.3	88.0	89.1	89.4	96.1	88.7
	**Weak new marker (adjusted HR=1.2)**					**Null new marker (adjusted HR=1.0)**				
	ΔC	3catNRI	2catNRI	NRI>0	IDI	ΔC	3catNRI	2catNRI	NRI>0	IDI
**Bias Corrected**	91.7	81.9	87.6	91.5	91.1	98.8	87.6	82.3	96.0	96.4
**Percentile**	97.7	100.0	100.0	98.1	97.7	98.9	100.0	100.0	98.5	97.2
**Bootstrap-t**	88.5	80.6	82.6	89.3	89.6	96.6	79.0	86.5	89.3	98.8
**Hybrid**	82.8	86.5	88.2	91.4	73.0	100.0	93.3	93.5	93.1	100.0

3catNRI assumes categories [0.00, 0.40), [0.40, 0.60), and [0.60+).

2catNRI assumes categories [0.00, 0.50) and [0.50+).

Supplementary Figure S12. Bias for 10% event rate and n=2000

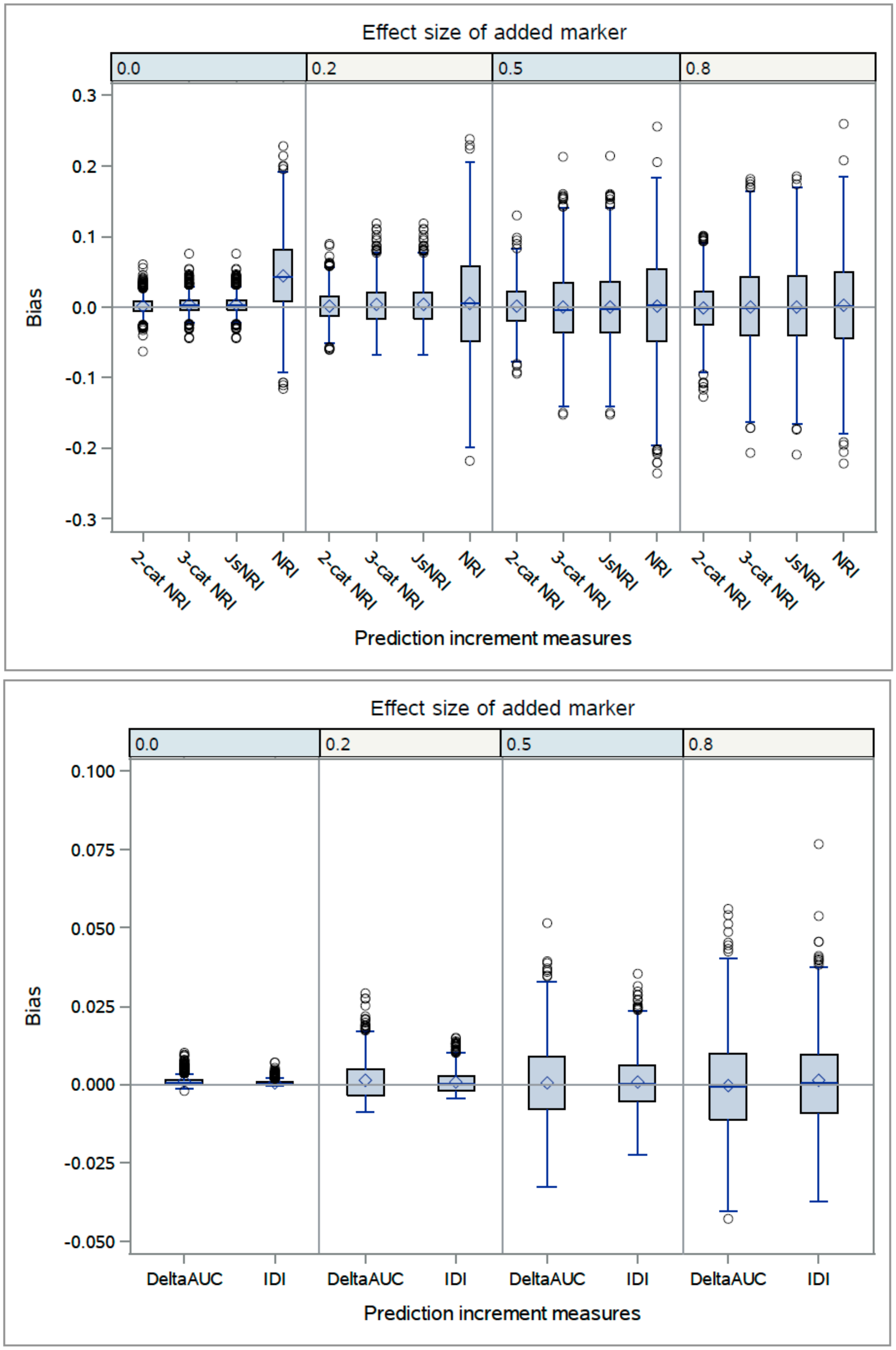

Supplementary Figure S13. Bias for 10% event rate and n=300

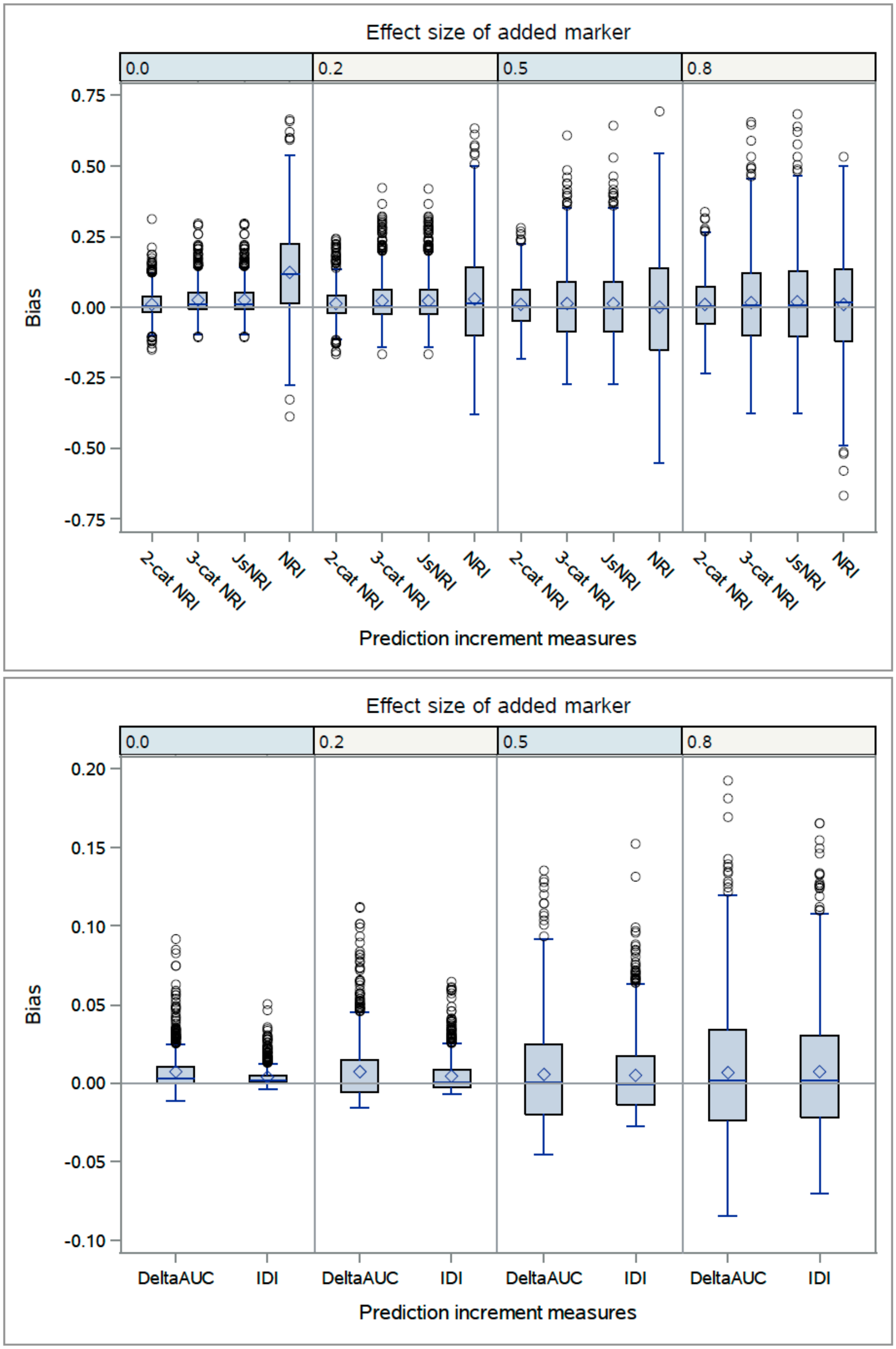

Supplementary Figure S14. Bias for 50% event rate and n=2000

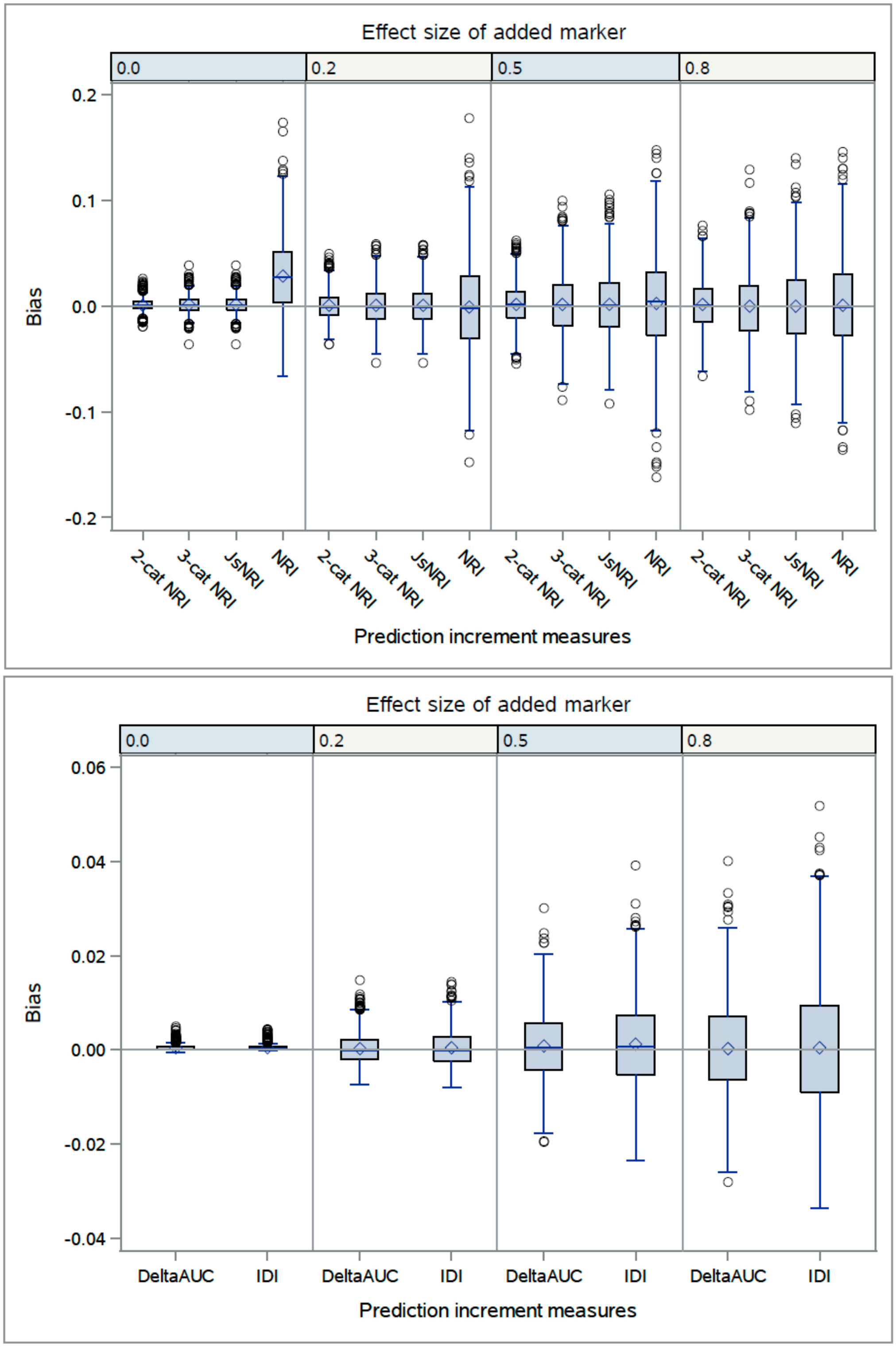

Supplementary Figure S15. Bias for 50% event rate and n=300

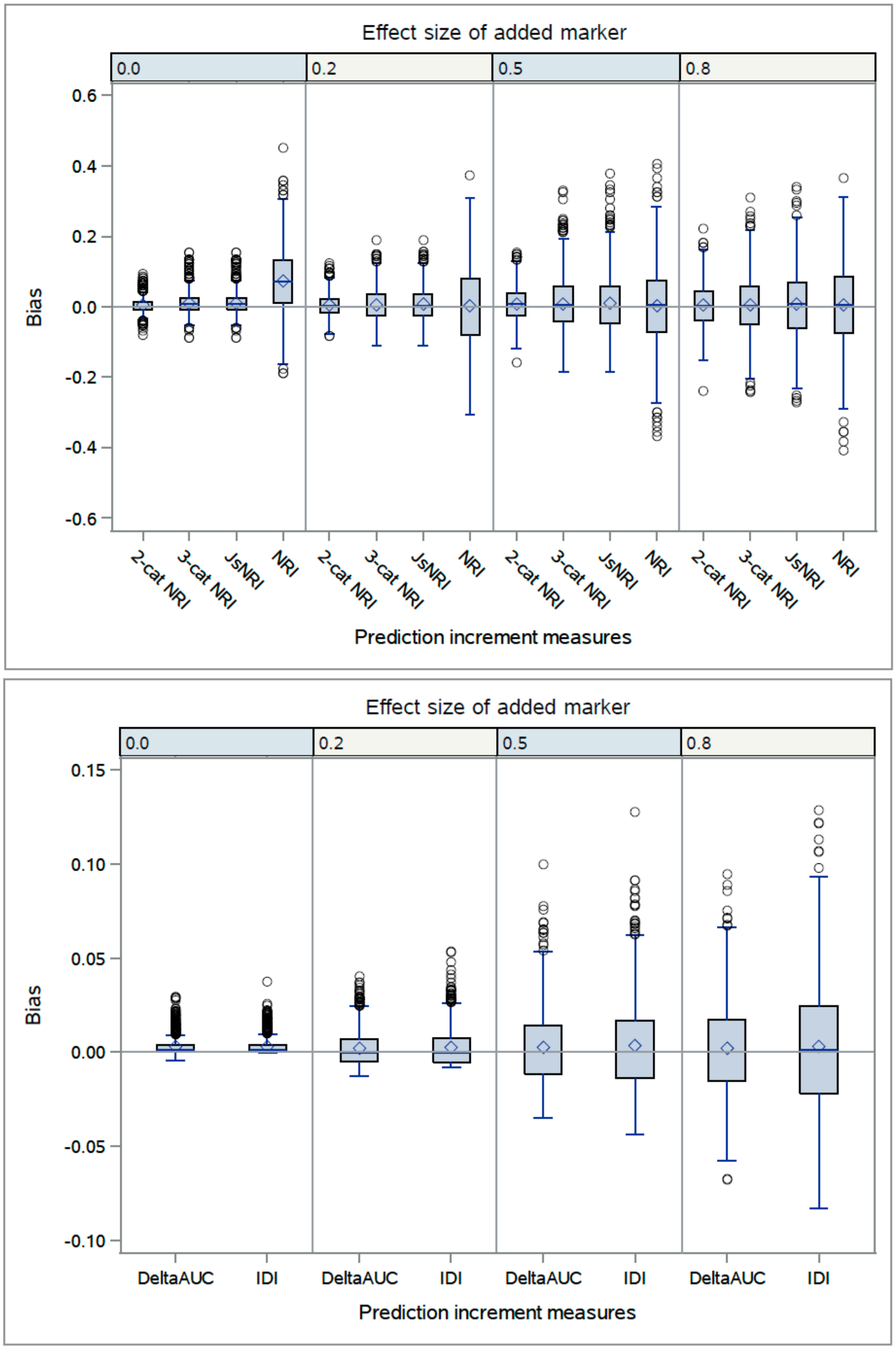

Supplementary Figure S16. Bias for 10% incidence rate, Type I censoring, and n=2000

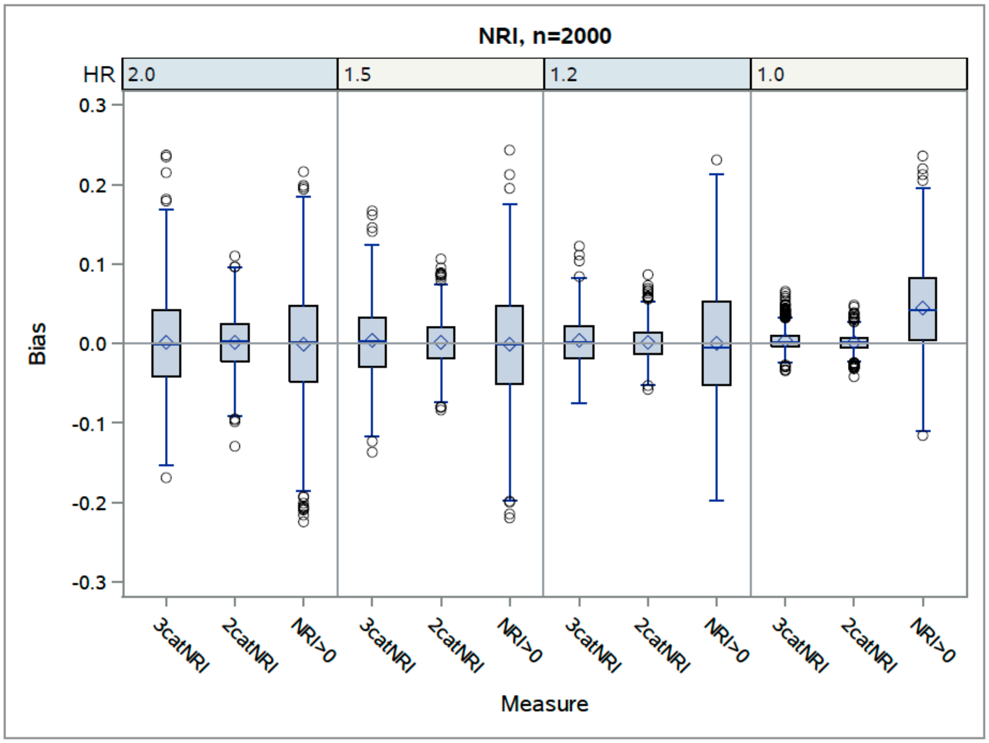

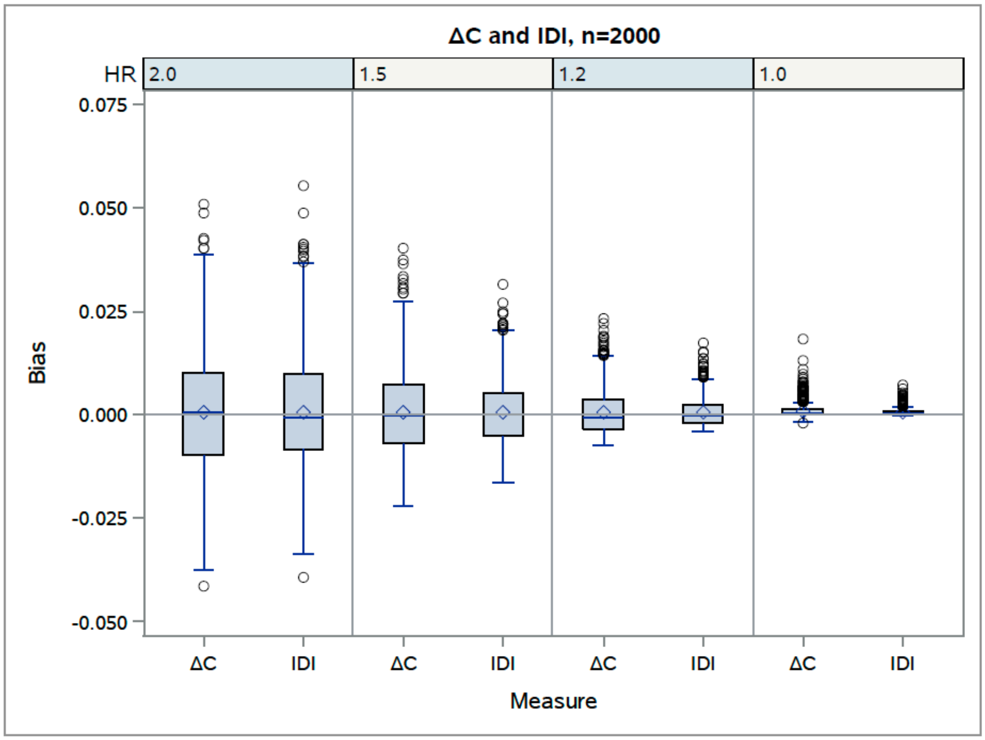

Supplementary Figure S17. Bias for 10% incidence rate, Type I censoring, and n=300

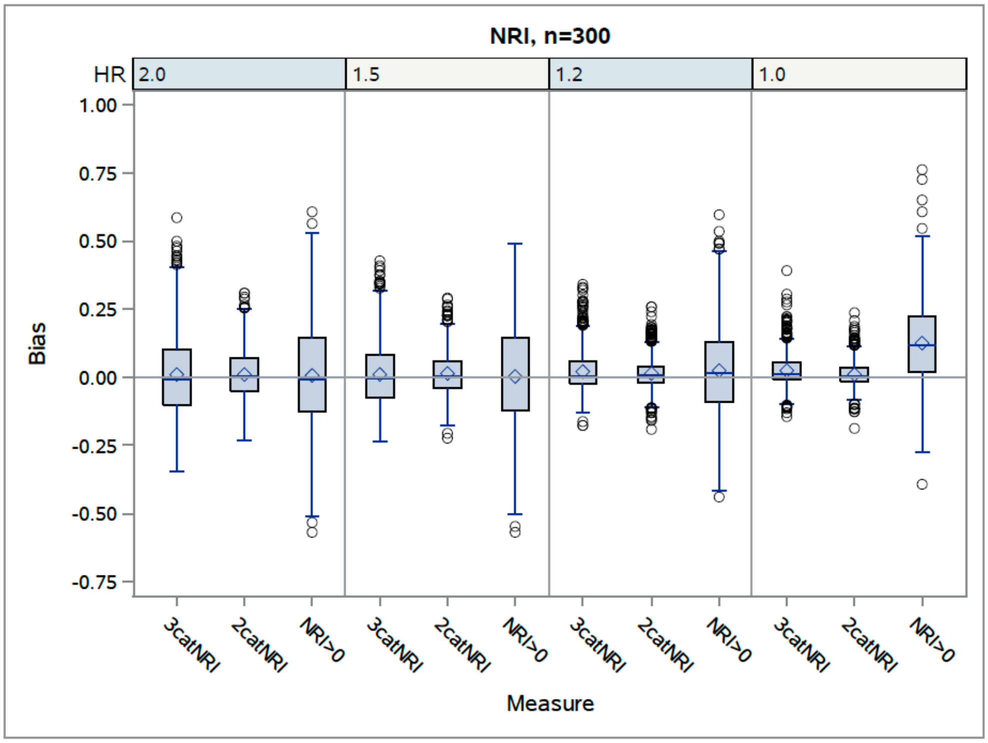

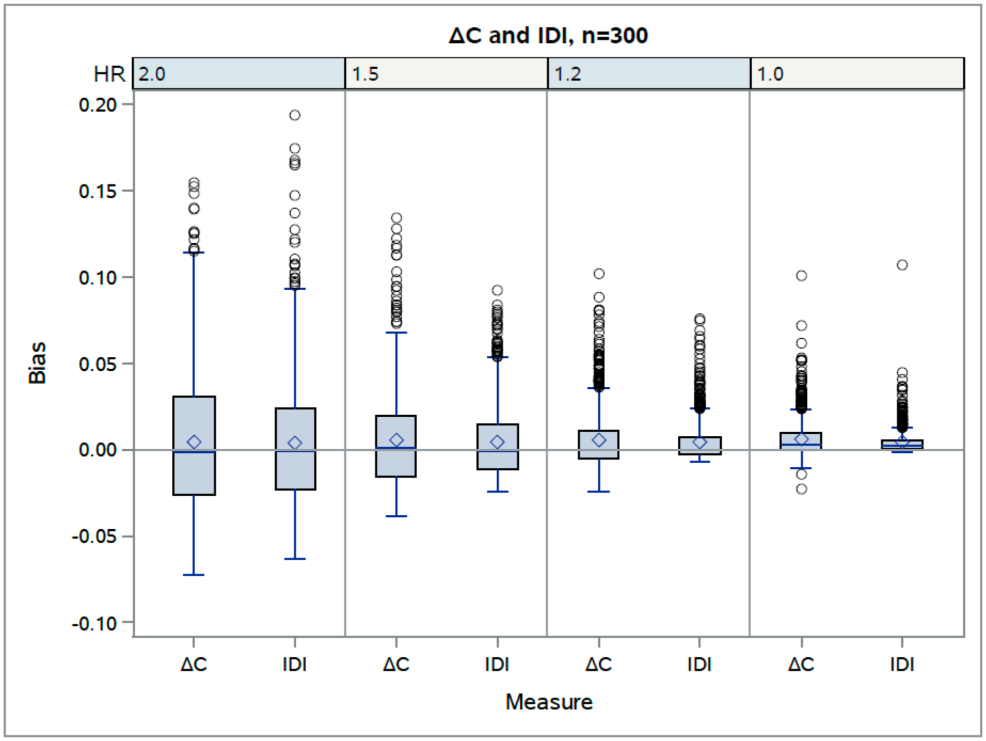

Supplementary Figure S18. Bias for 50% incidence rate, Type I censoring, and n=2000

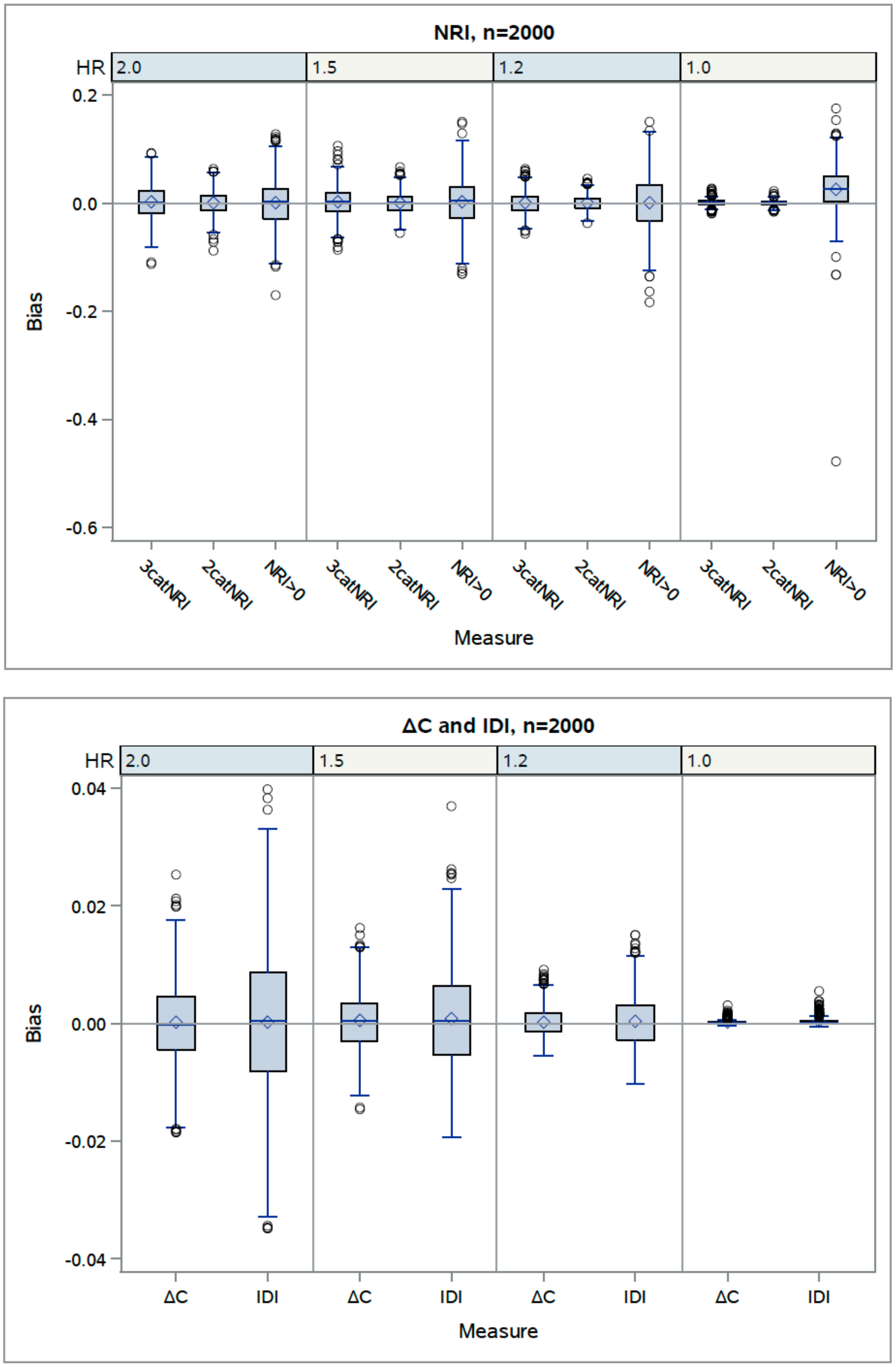

Supplementary Figure S19. Bias for 50% incidence rate, Type I censoring, and n=300

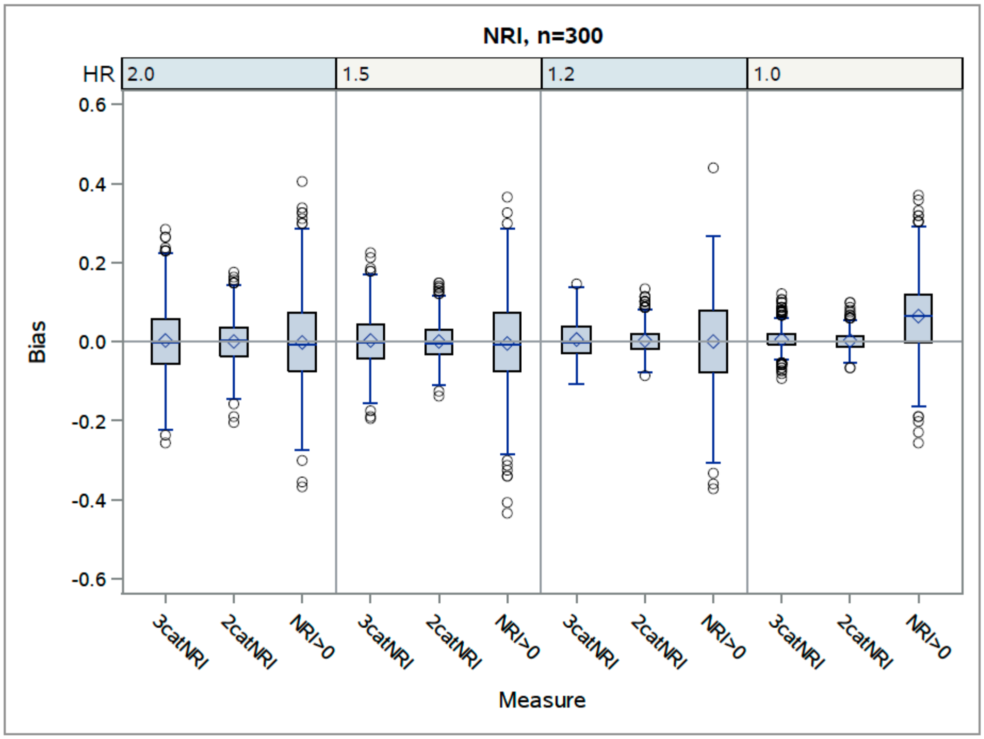

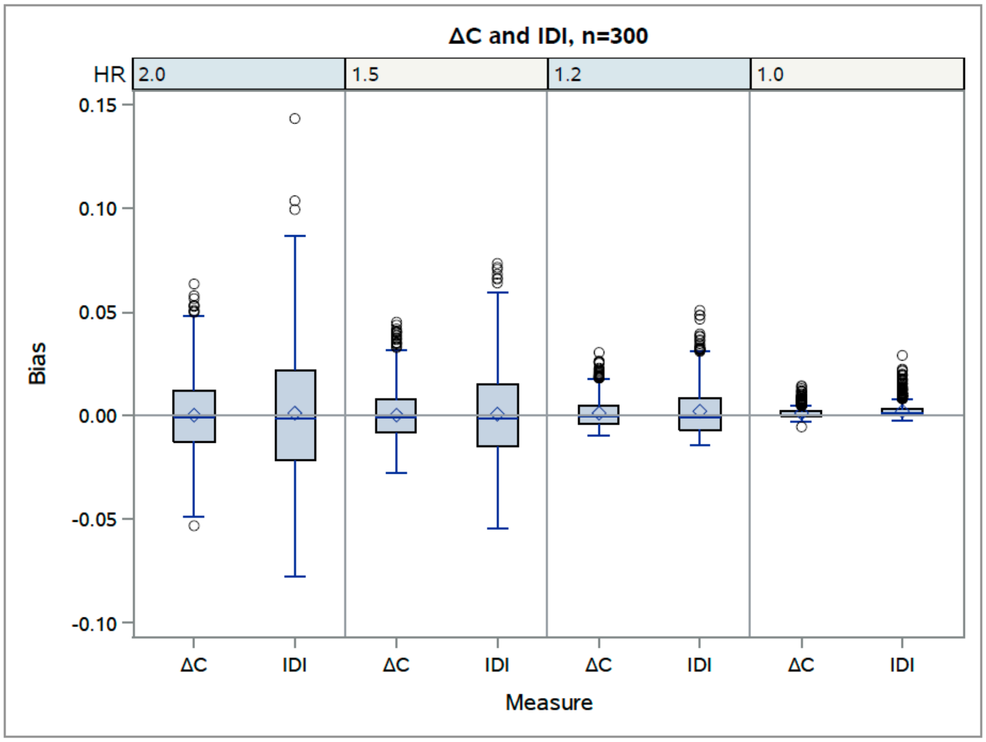

Supplementary Figure S20. Bias for 10% incidence rate, random censoring, and n=2000

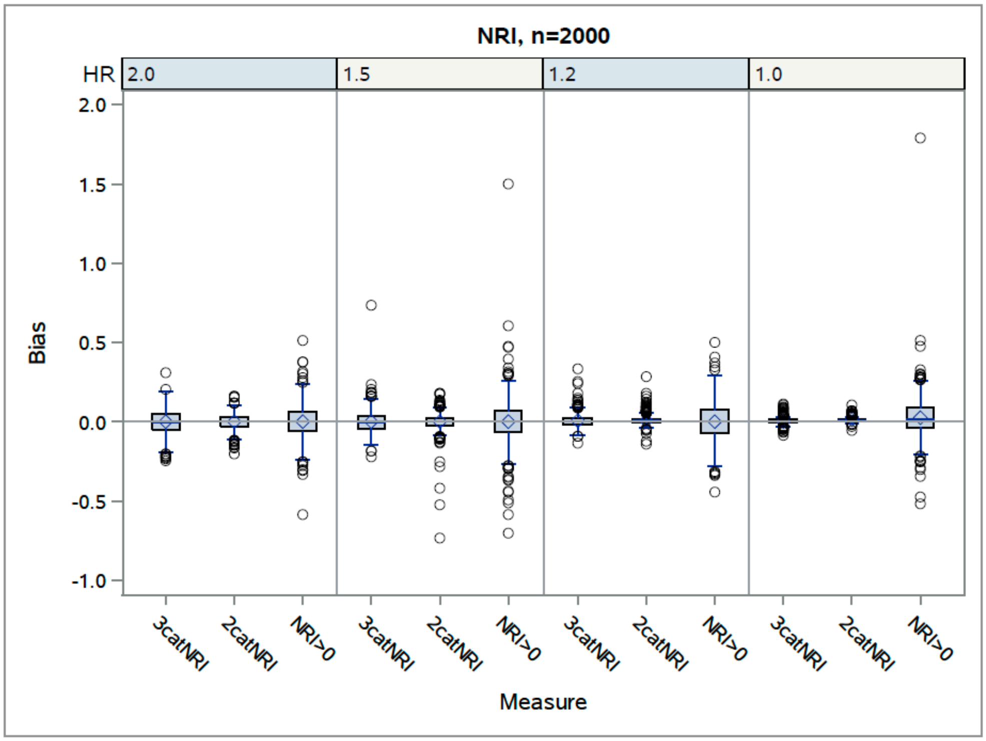

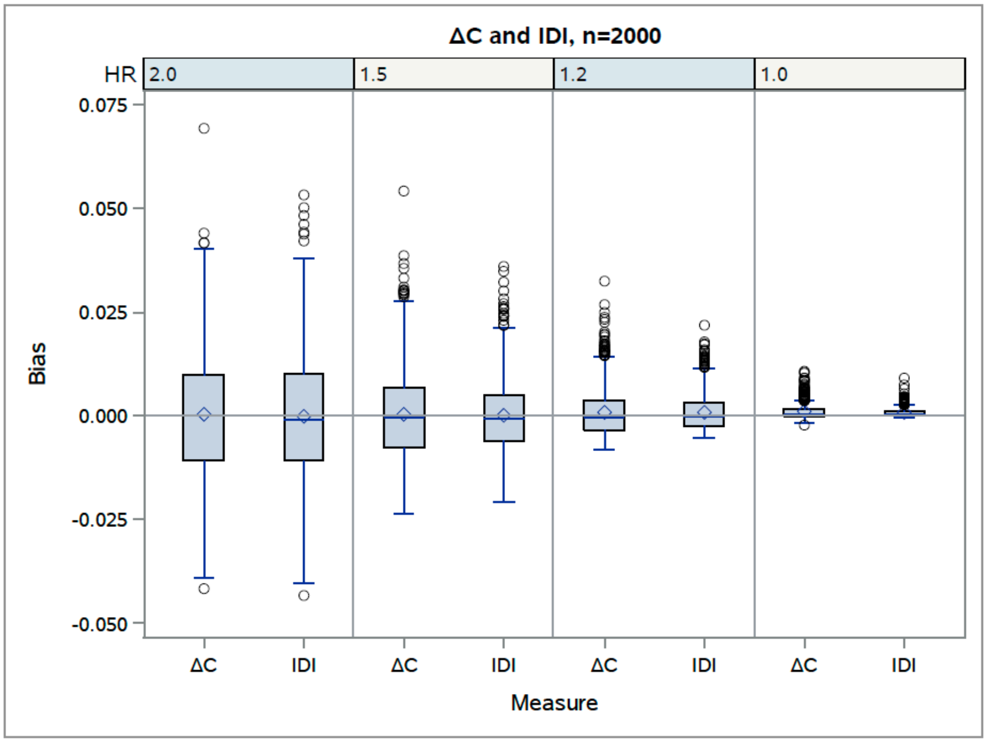

Supplementary Figure S21. Bias for 10% incidence rate, random censoring, and n=300

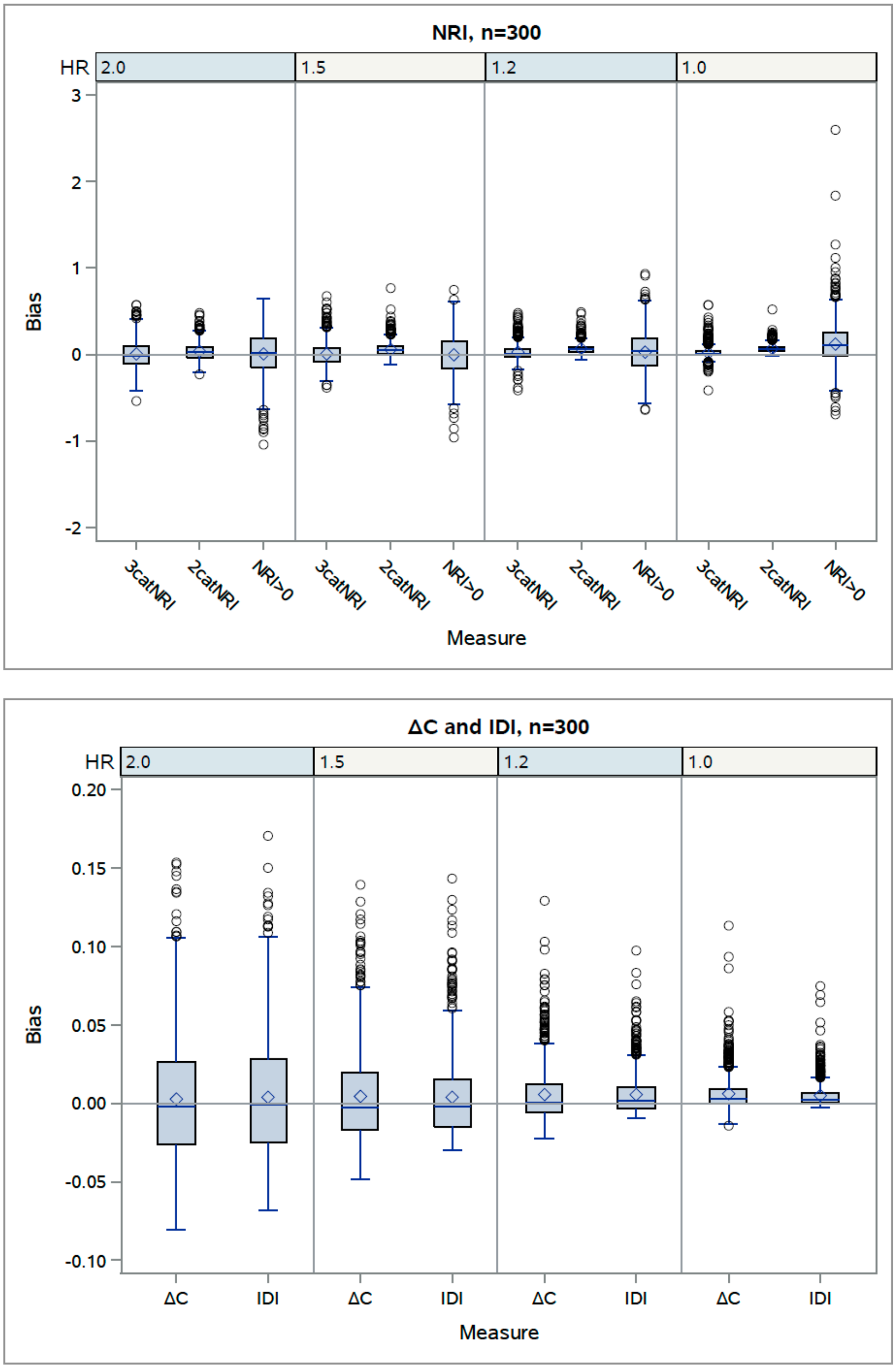

Supplementary Figure S22. Bias for 50% incidence rate, random censoring, and n=2000

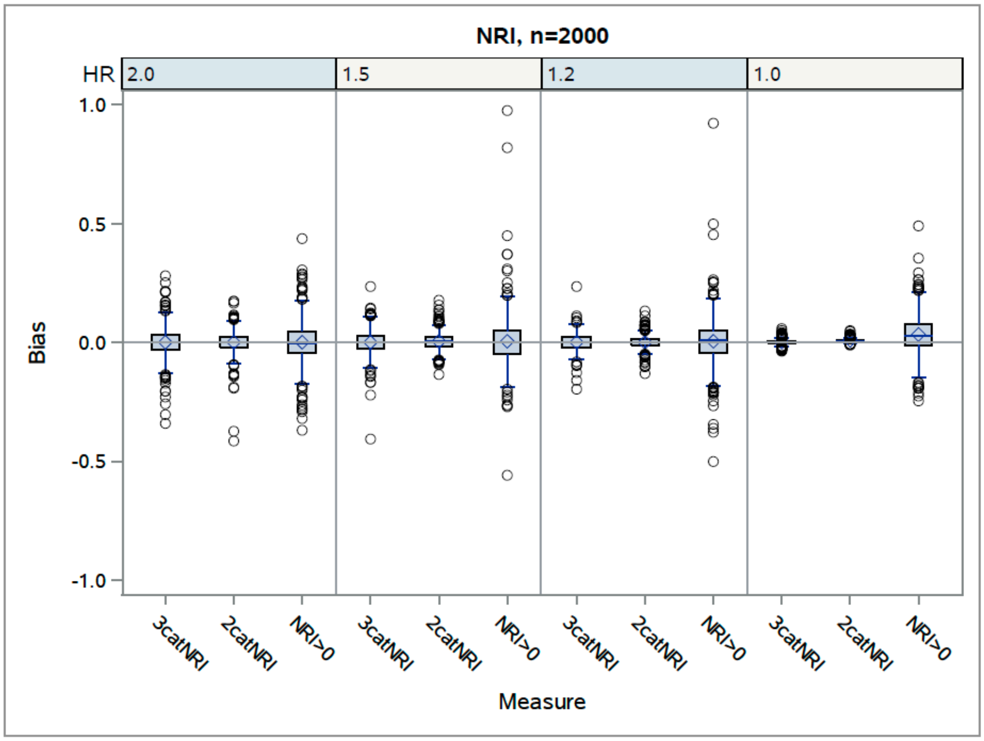

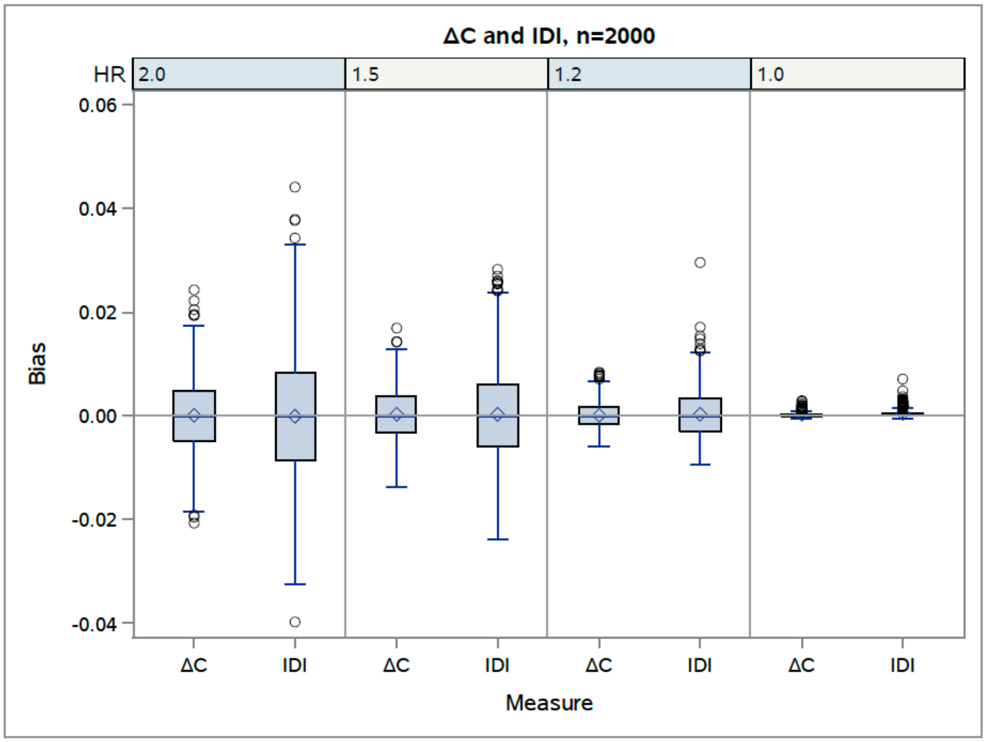

Supplementary Figure S23. Bias for 50% incidence rate, random censoring, and n=300

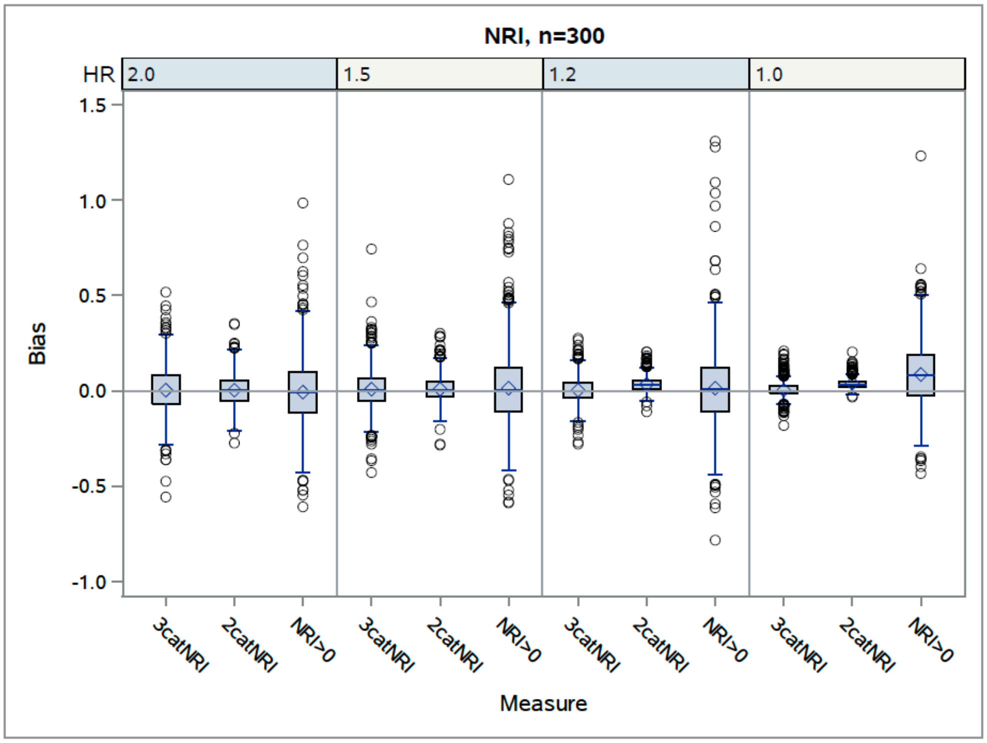

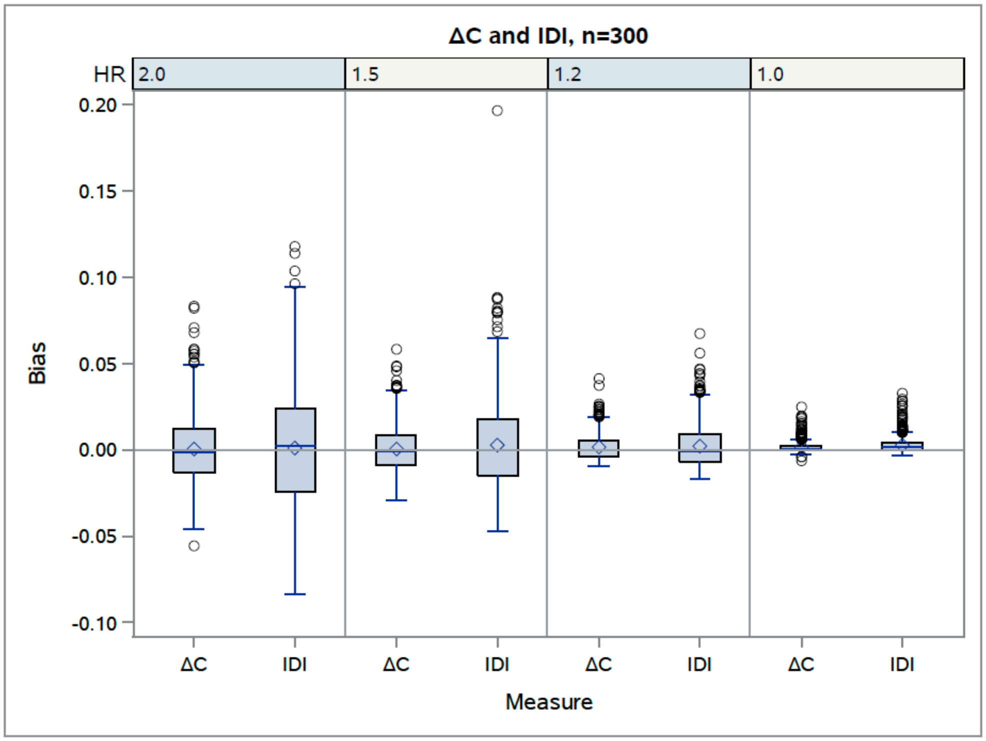

Supplementary Figure S24. CI widths for ΔAUC with n=300

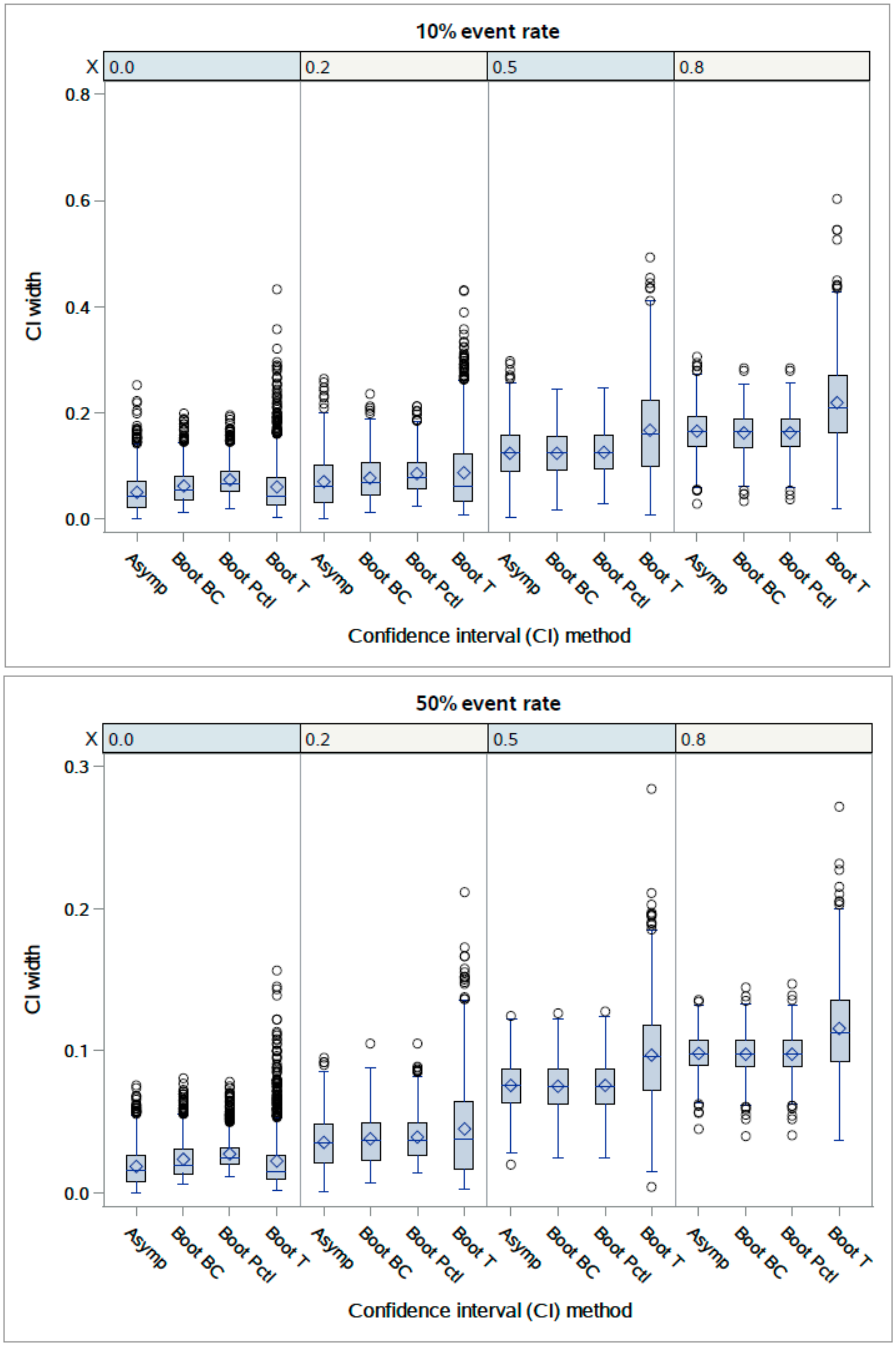

Supplementary Figure S25. CI widths for 3catNRI with n=300

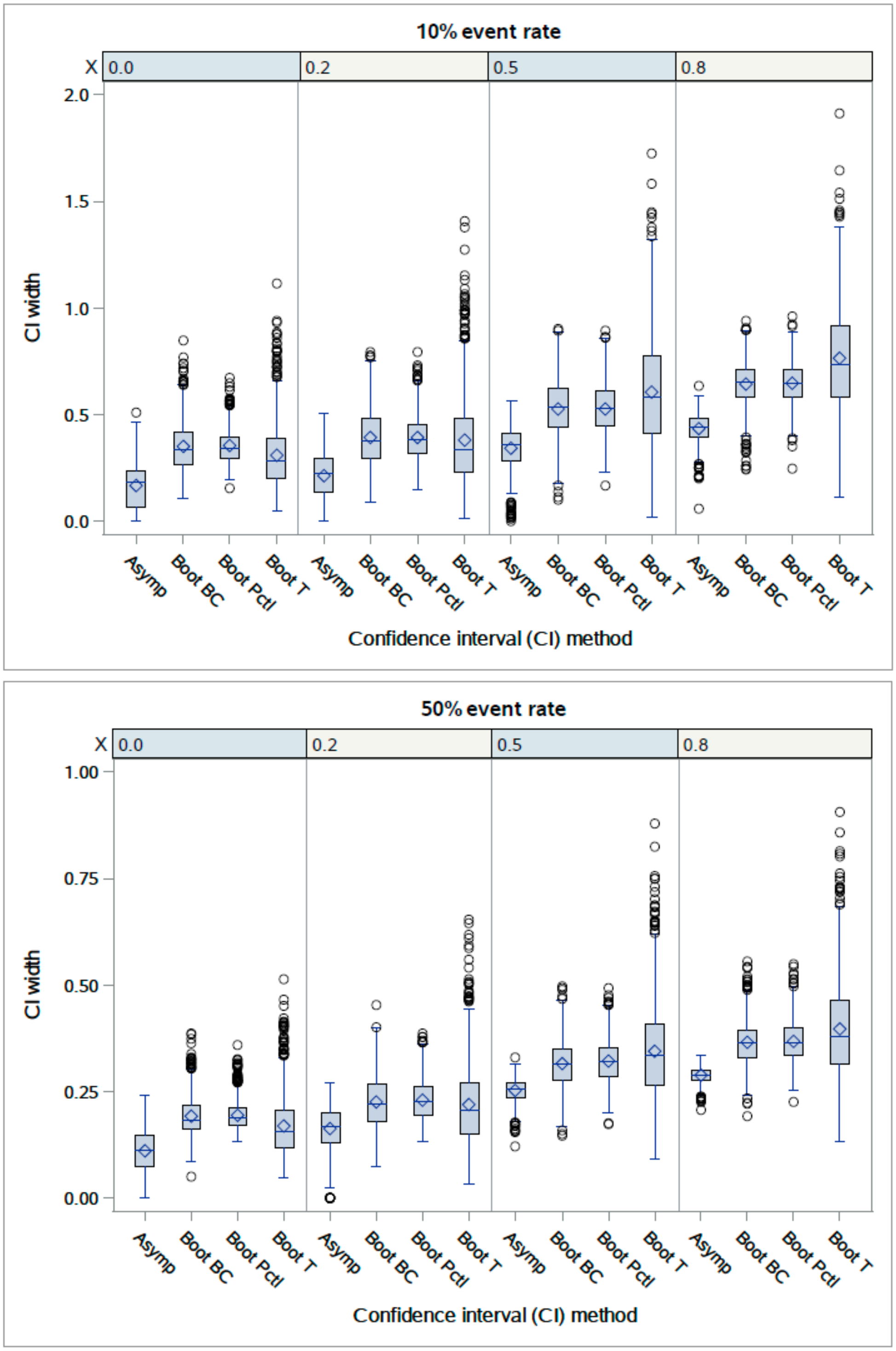

Supplementary Figure S26. CI widths for 2catNRI with n=300

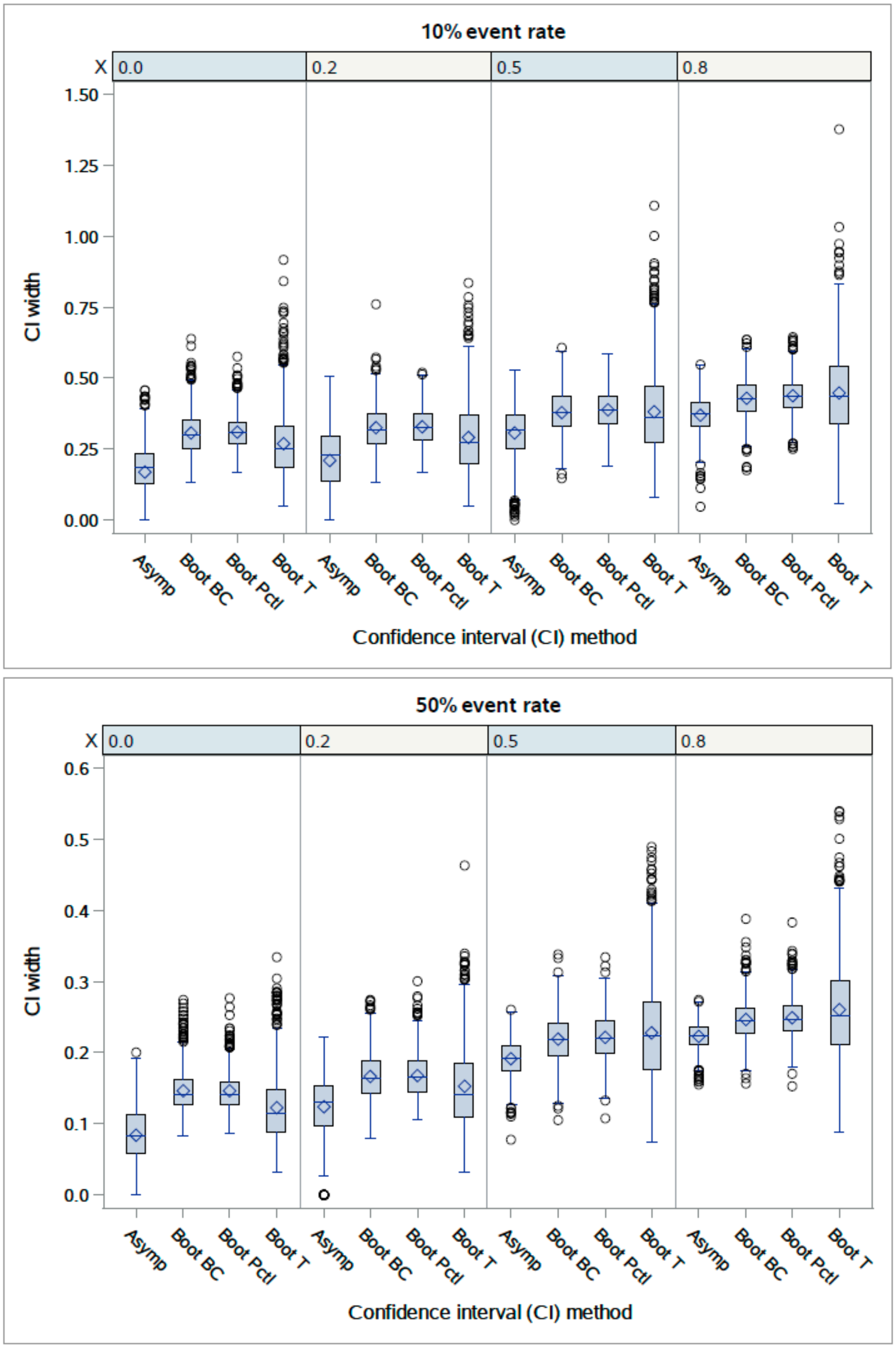

Supplementary Figure S27. CI widths for NRI>0 with n=300

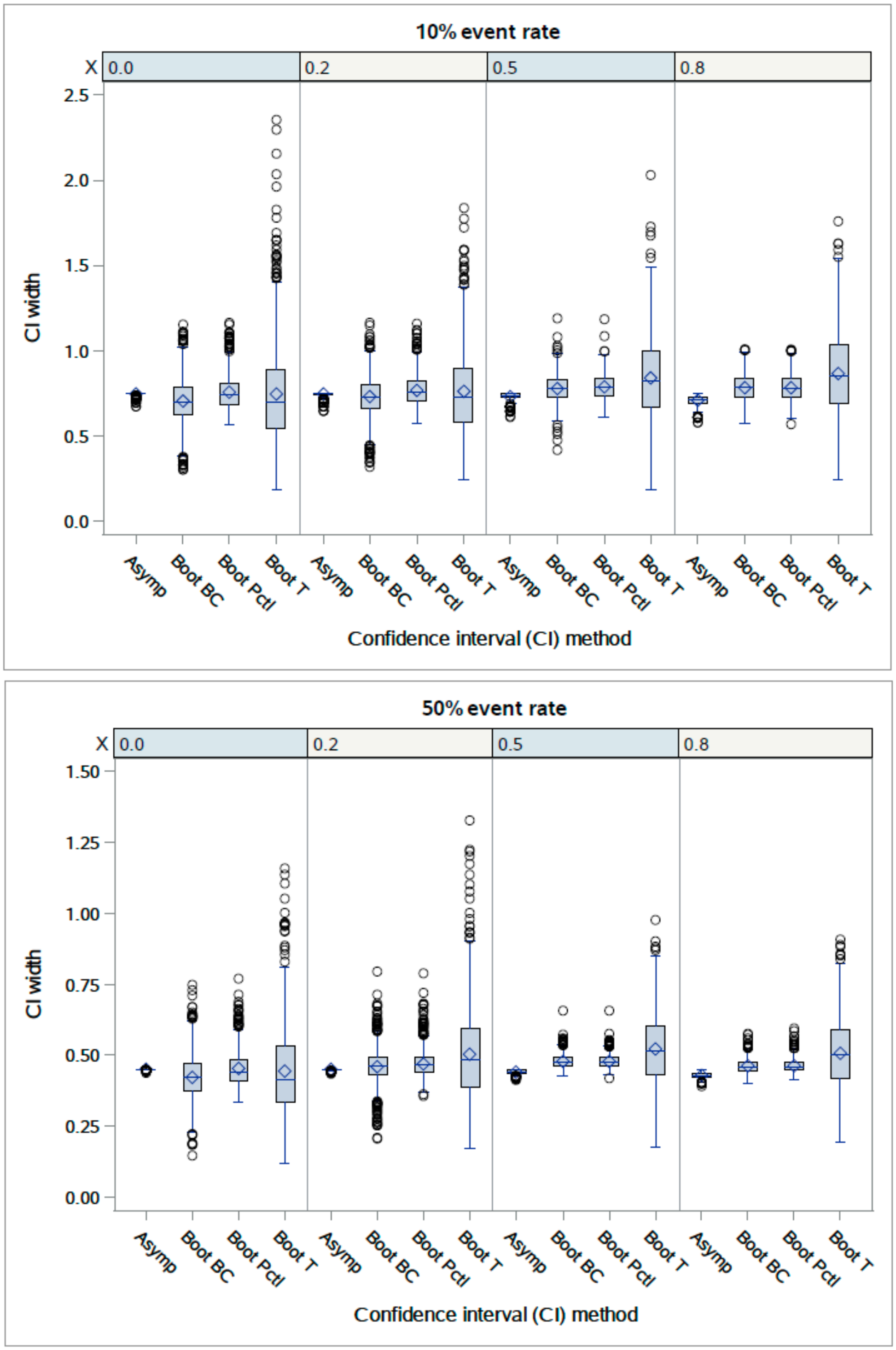

Supplementary Figure S28. CI widths for IDI with n=300

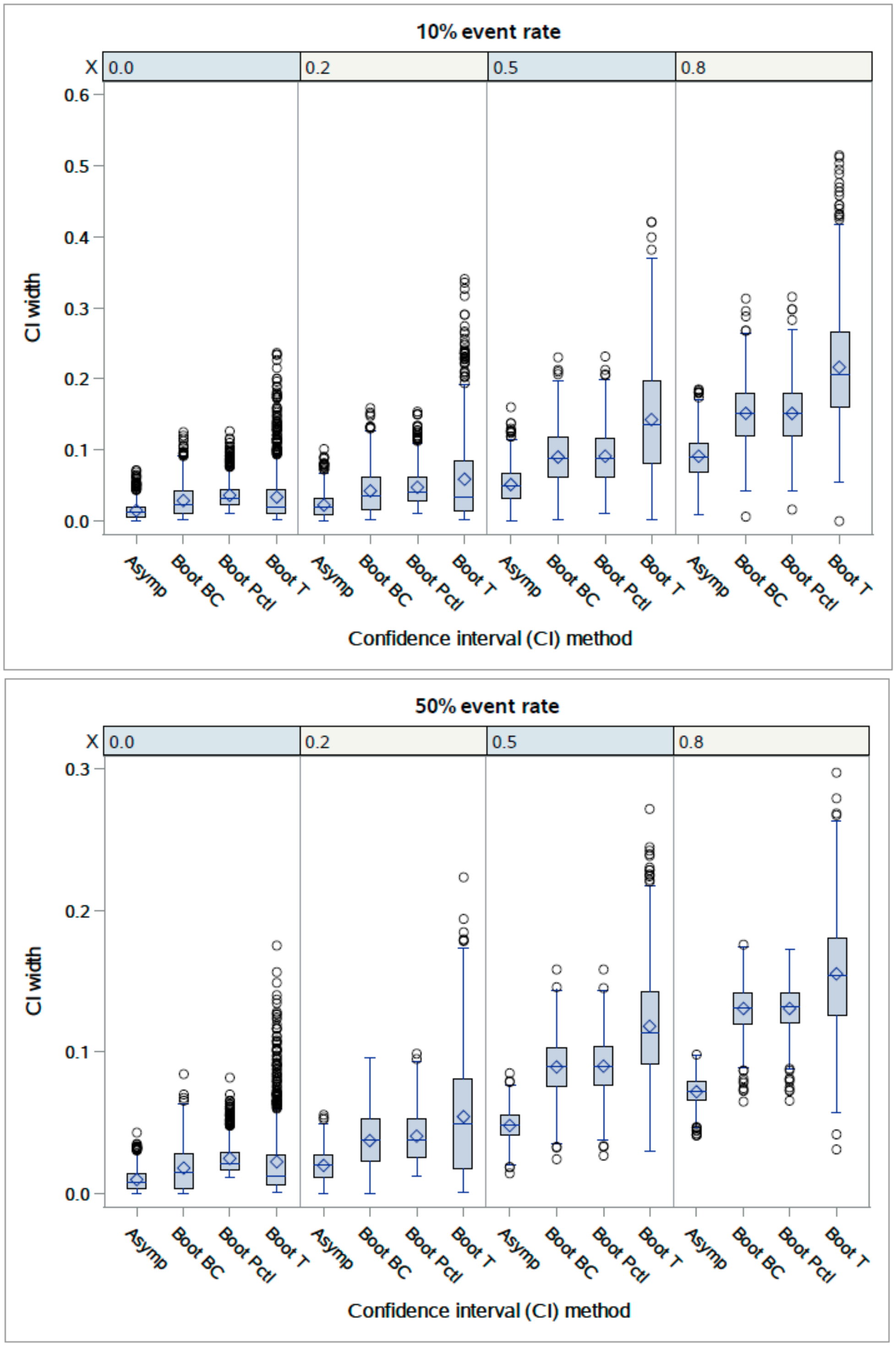

Supplementary Figure S29. CI widths for ΔC with type I censoring (n=300)

A. 10% incidence rate

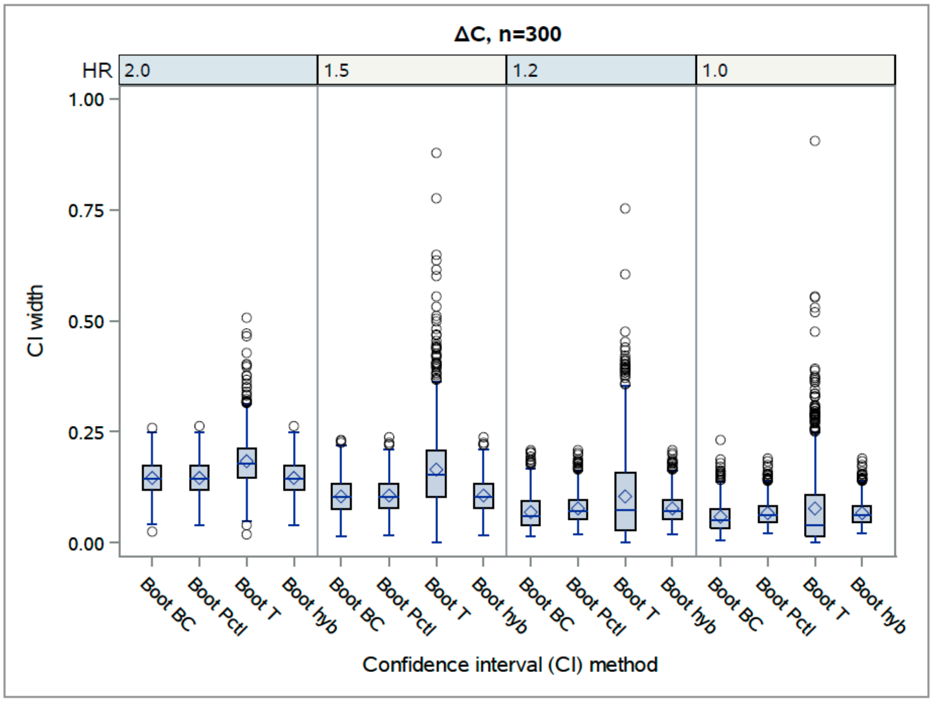

B. 50% incidence rate

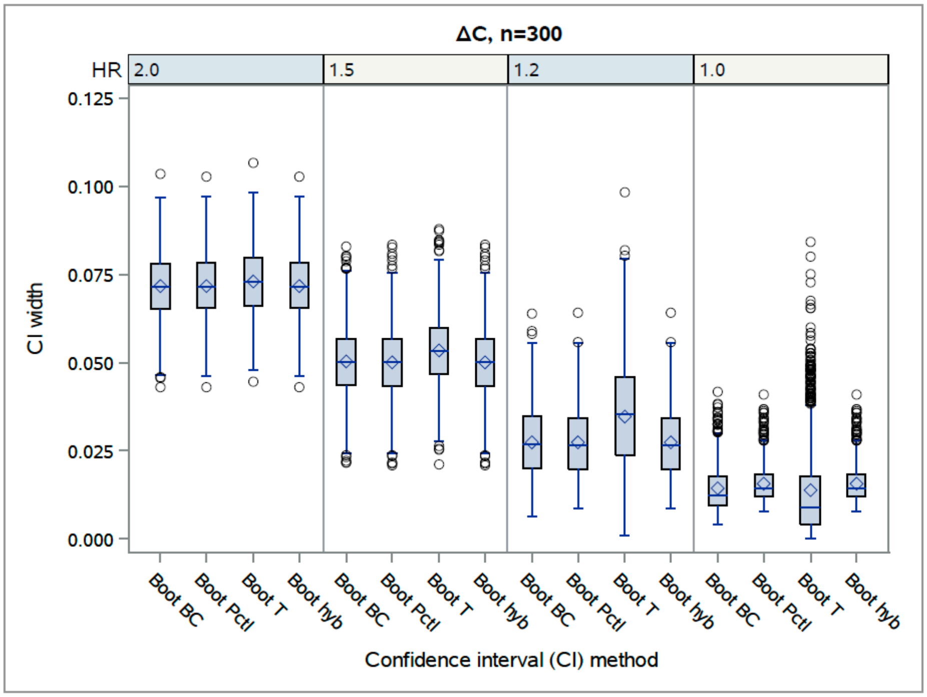

Supplementary Figure S30. CI widths for 3catNRI with type I censoring (n=300)

A. 10% incidence rate

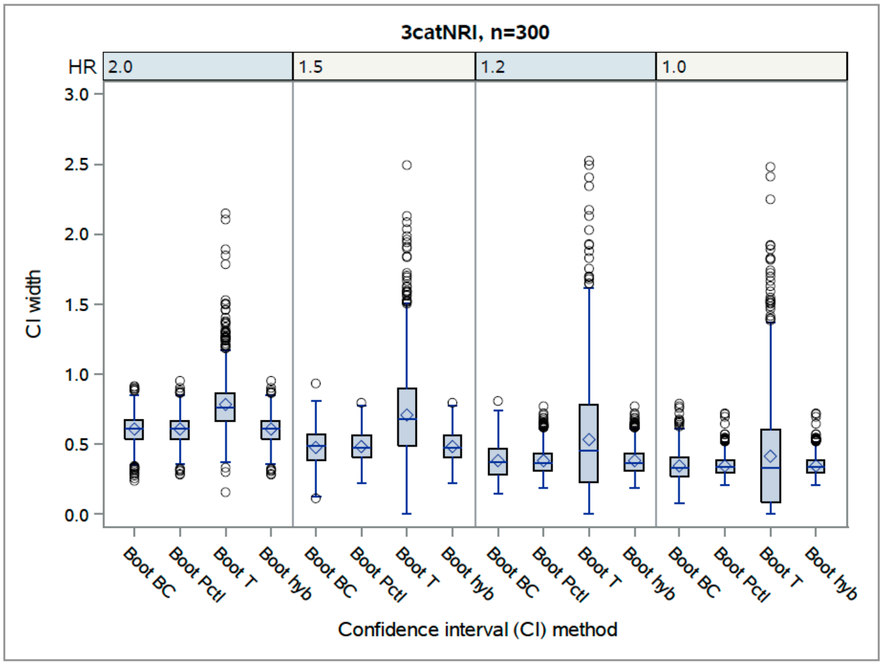

B. 50% incidence rate

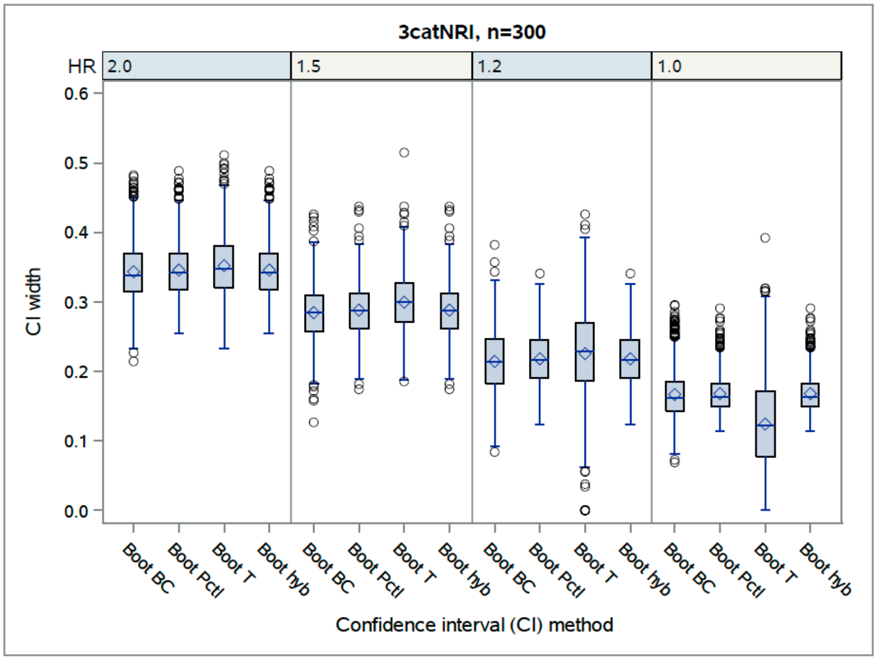

Supplementary Figure S31. CI widths for 2catNRI with type I censoring (n=300)

A. 10% incidence rate

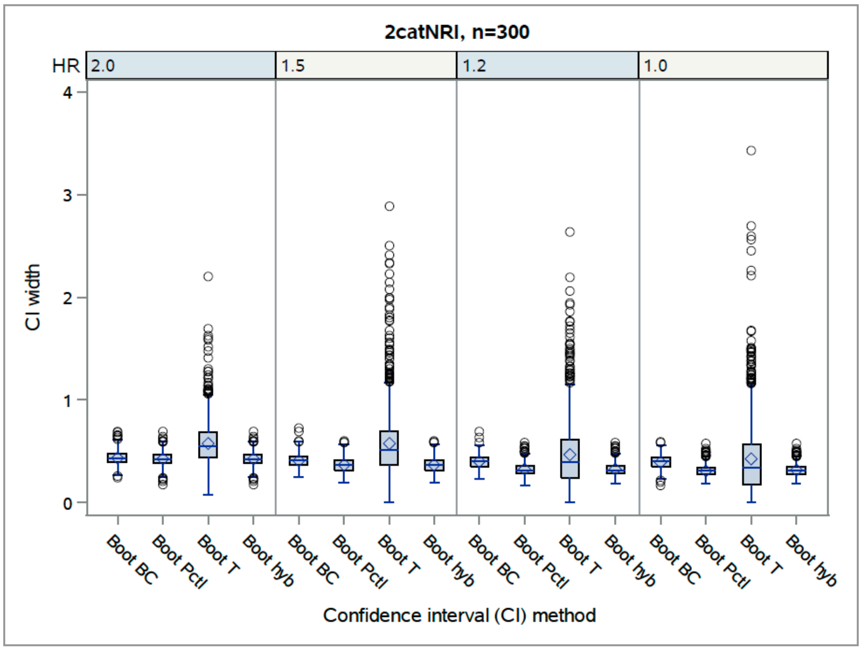

B. 50% incidence rate

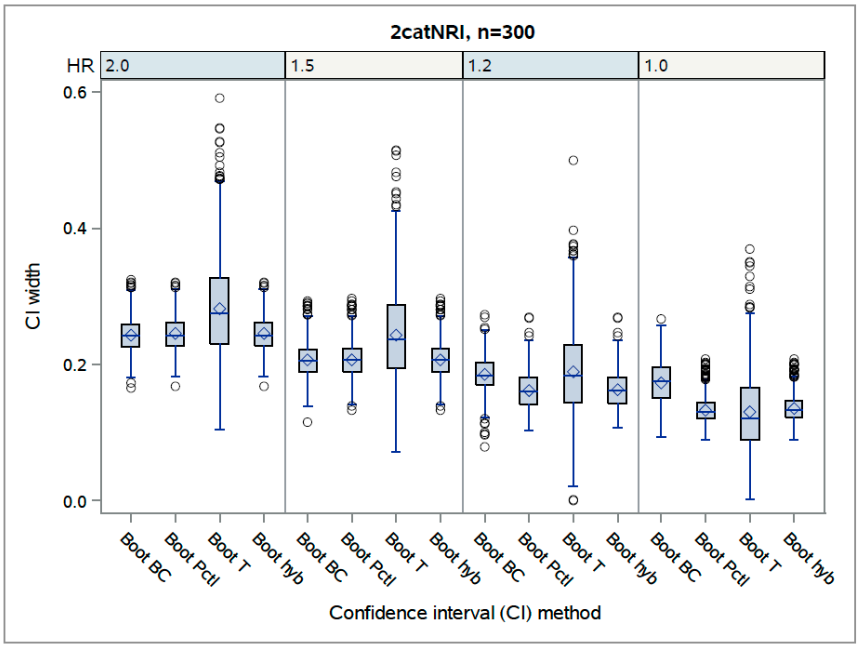

Supplementary Figure S32. CI widths for NRI>0 with type I censoring (n=300)

A. 10% incidence rate

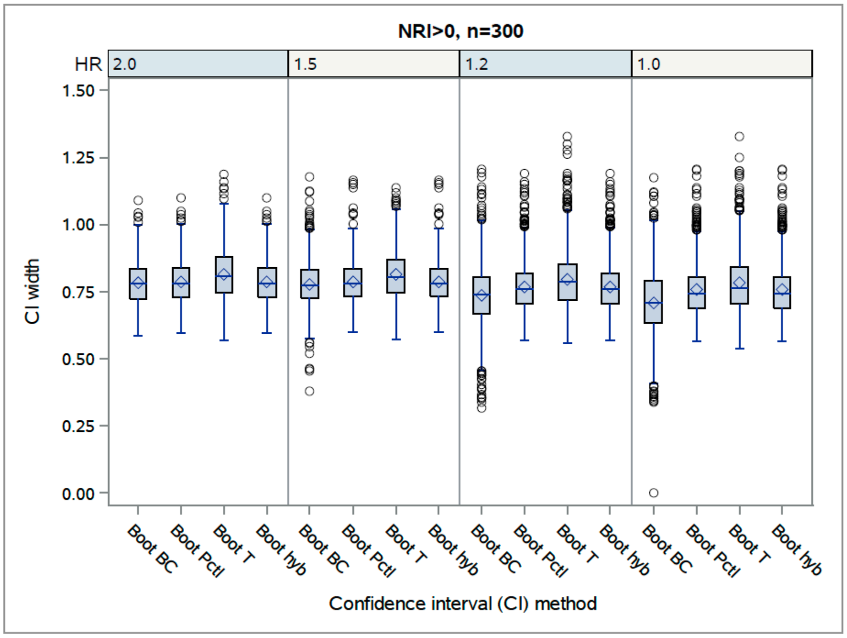

B. 50% incidence rate

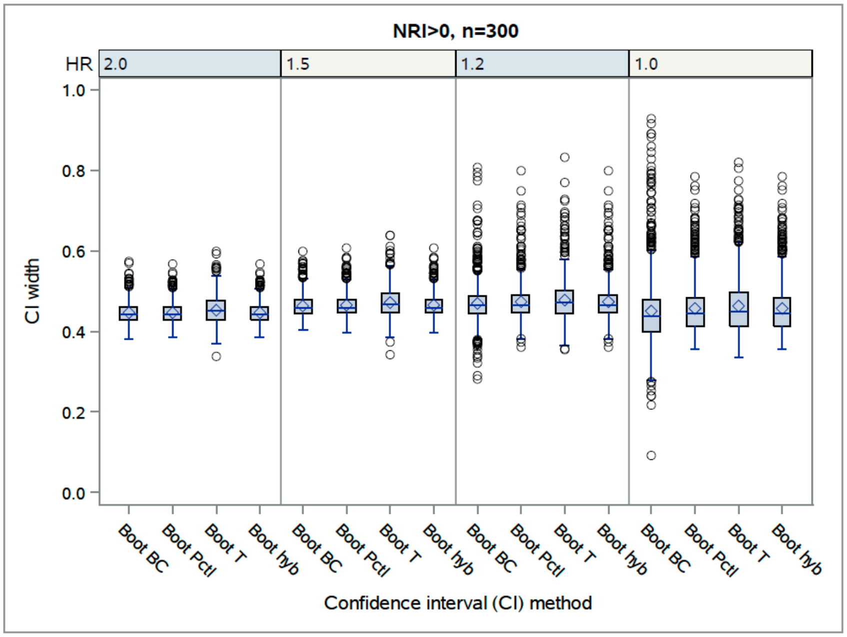

Supplementary Figure S33. CI widths for IDI with type I censoring (n=300)

A. 10% incidence rate

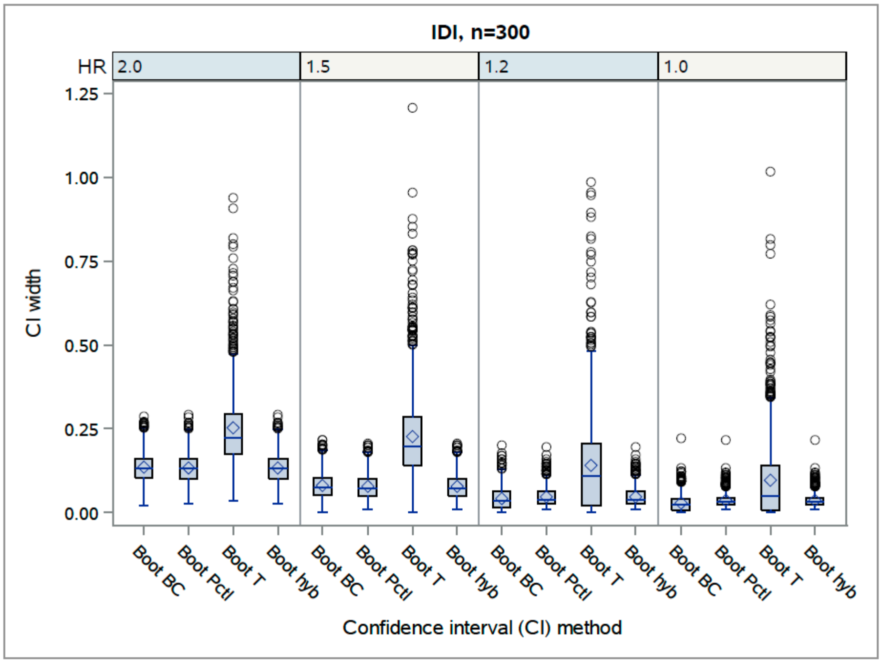

B. 50% incidence rate

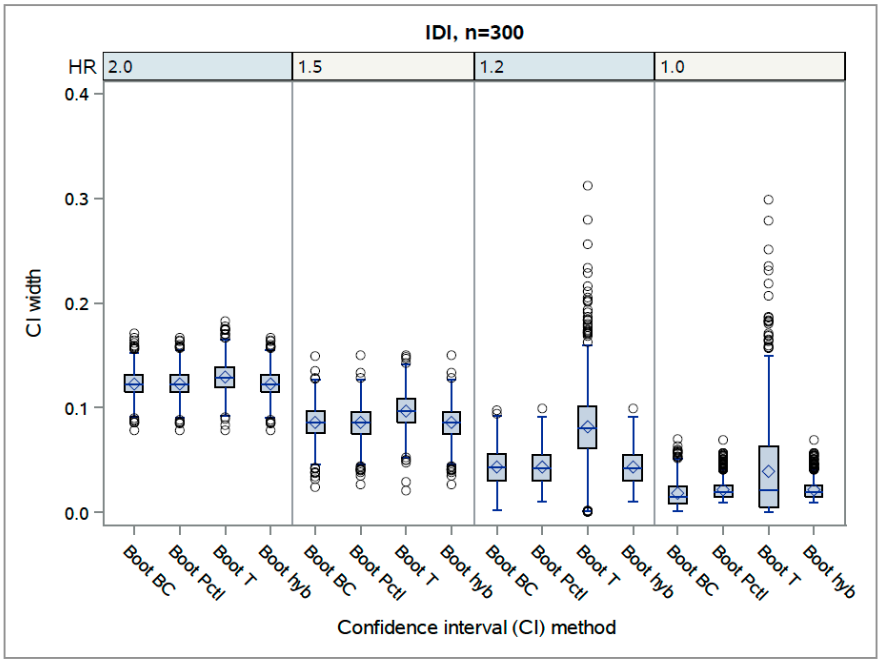

Supplementary Figure S34. CI widths for ΔC with random censoring (n=300)

A. 10% incidence rate

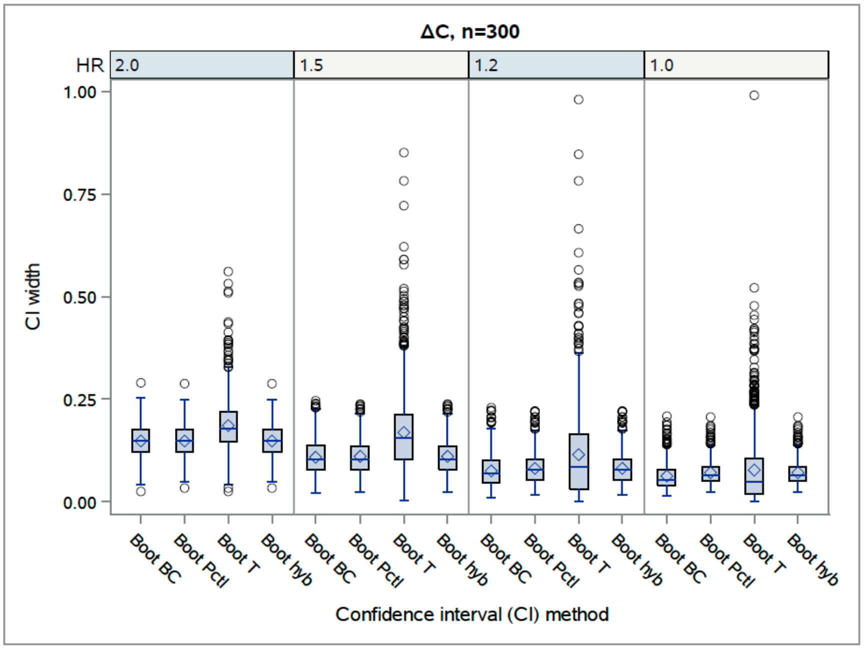

B. 50% incidence rate

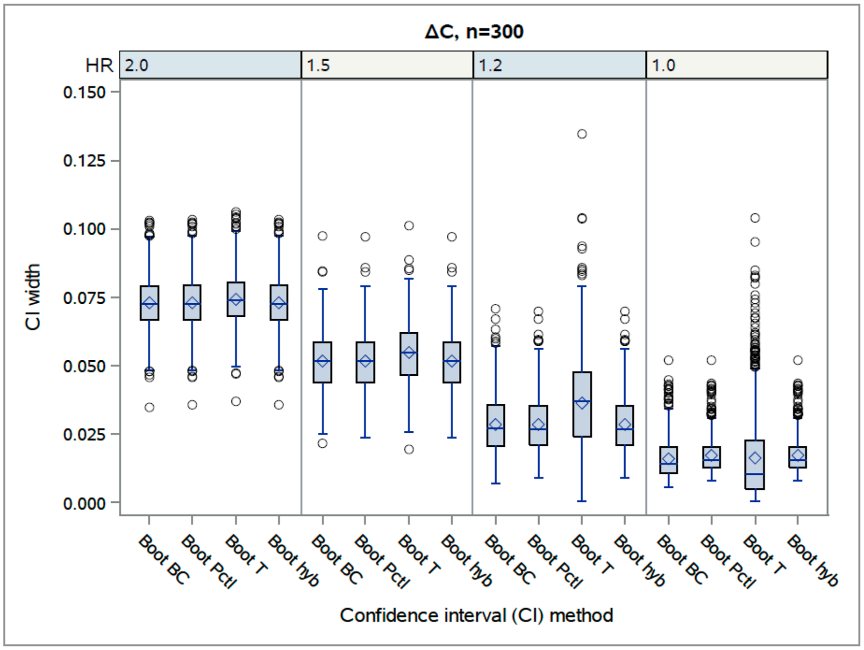

Supplementary Figure S35. CI widths for 3catNRI with random censoring (n=300)

A. 10% incidence rate

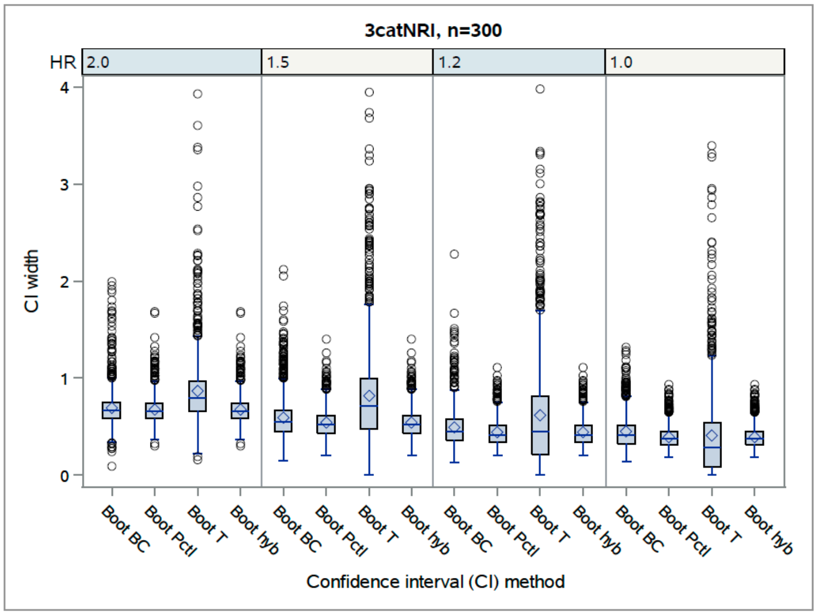

B. 50% incidence rate

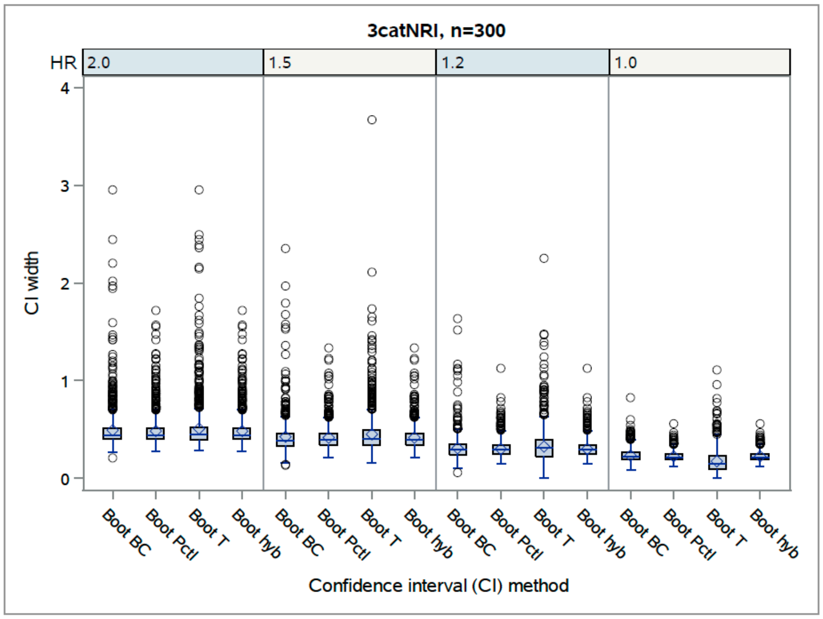

Supplementary Figure S36. CI widths for 2catNRI with random censoring (n=300)

A. 10% incidence rate

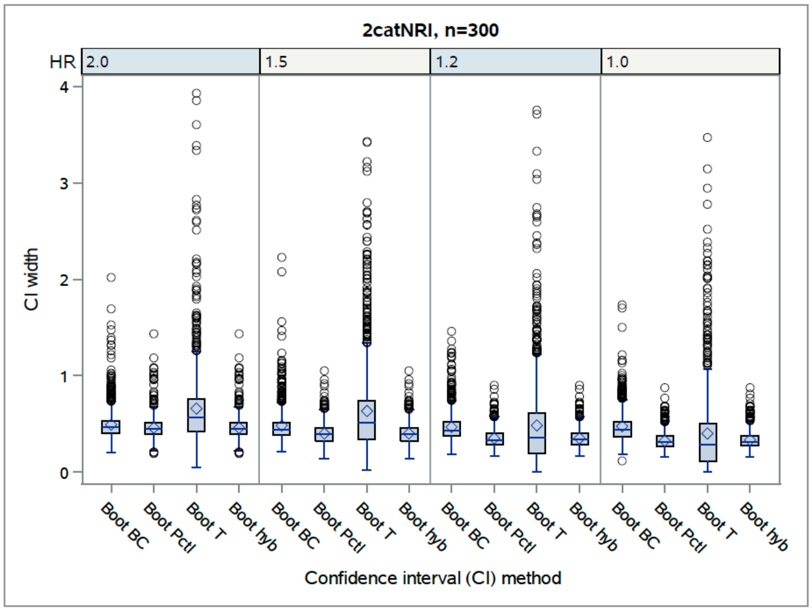

B. 50% incidence rate

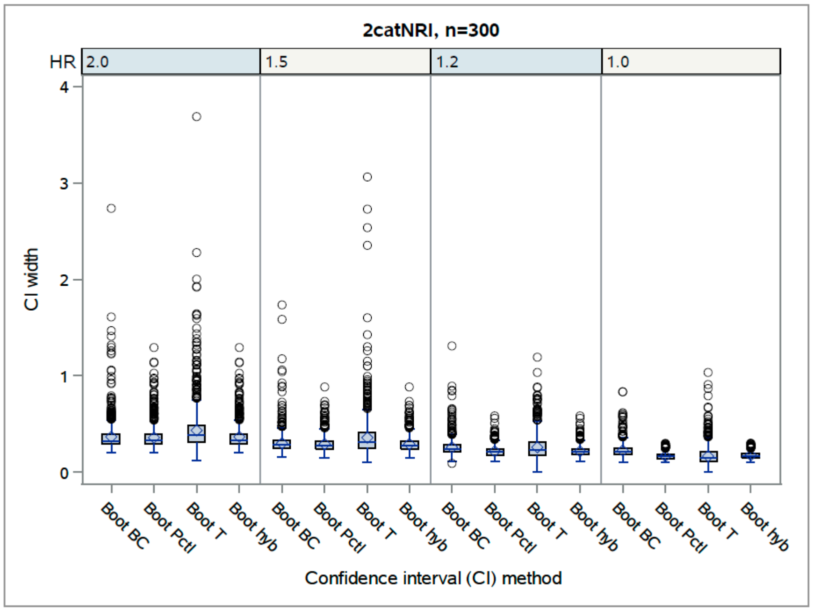

Supplementary Figure S37. CI widths for NRI>0 with random censoring (n=300)

A. 10% incidence rate

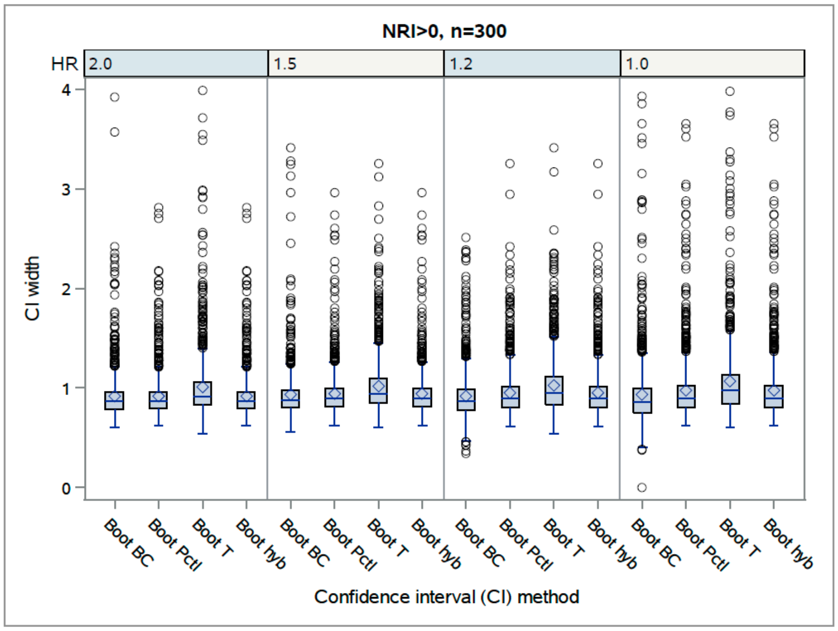

B. 50% incidence rate

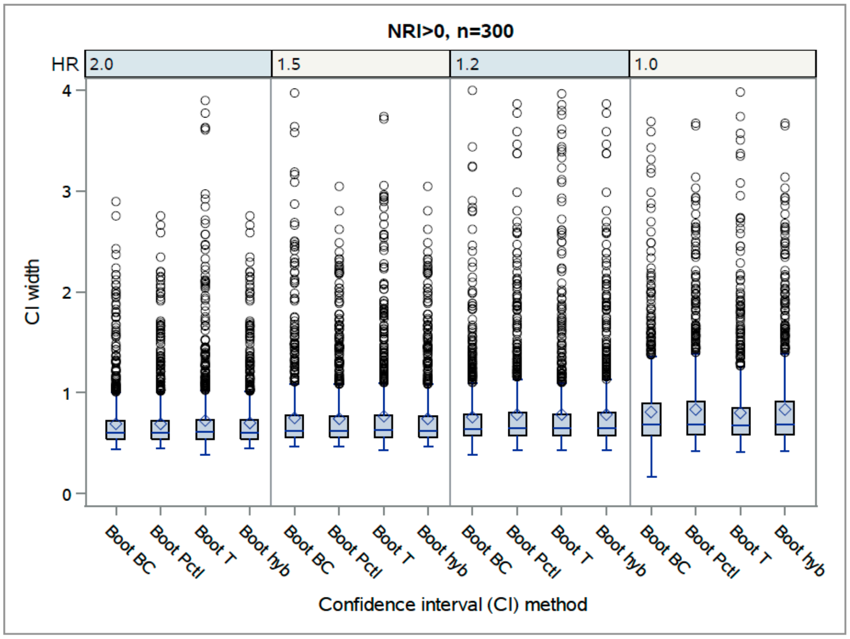

Supplementary Figure S38. CI widths for IDI with random censoring (n=300)

A. 10% incidence rate

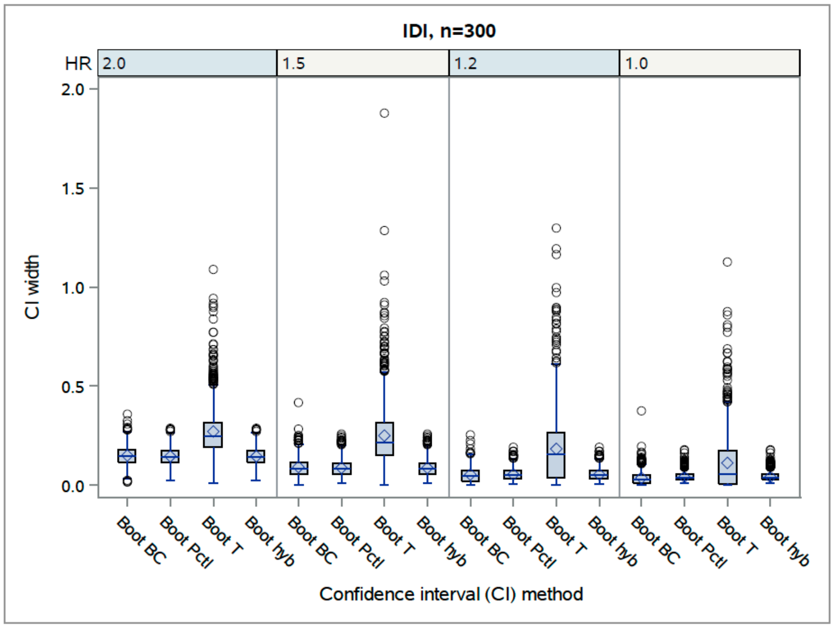

B. 50% incidence rate

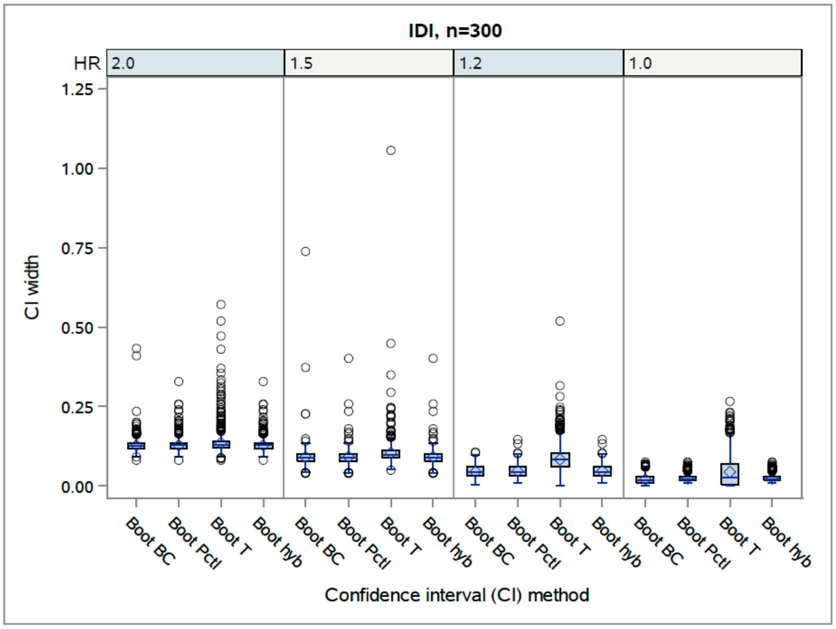

Supplement: Supplementary file 1 [file cancers-17-01259-s001.zip › cancers-3551996-supplementary.pdf]
